# Photoredox Heterobimetallic Dual Catalysis Using Engineered Covalent Organic Frameworks

**DOI:** 10.1021/acscatal.1c03634

**Published:** 2021-09-21

**Authors:** Alberto López-Magano, Borja Ortín-Rubio, Inhar Imaz, Daniel Maspoch, José Alemán, Rubén Mas-Ballesté

**Affiliations:** † Inorganic Chemistry Department, Módulo 7, Universidad Autónoma de Madrid, 28049 Madrid, Spain; ‡ Catalan Institute of Nanoscience and Nanotechnology (ICN2), CSIC and The Barcelona Institute of Science and Technology, 08193 Barcelona, Spain; § Institució Catalana de Recerca y Estudis Avançats (ICREA), 08010 Barcelona, Spain; ∥ Institute for Advanced Research in Chemical Sciences (IAdChem), Universidad Autónoma de Madrid, 28049 Madrid, Spain; ⊥ Organic Chemistry Department, Módulo 1, Universidad Autónoma de Madrid, 28049 Madrid, Spain

**Keywords:** covalent organic frameworks, photocatalysis, dual catalysis, C−C bond formation, heterogeneous catalysis

## Abstract

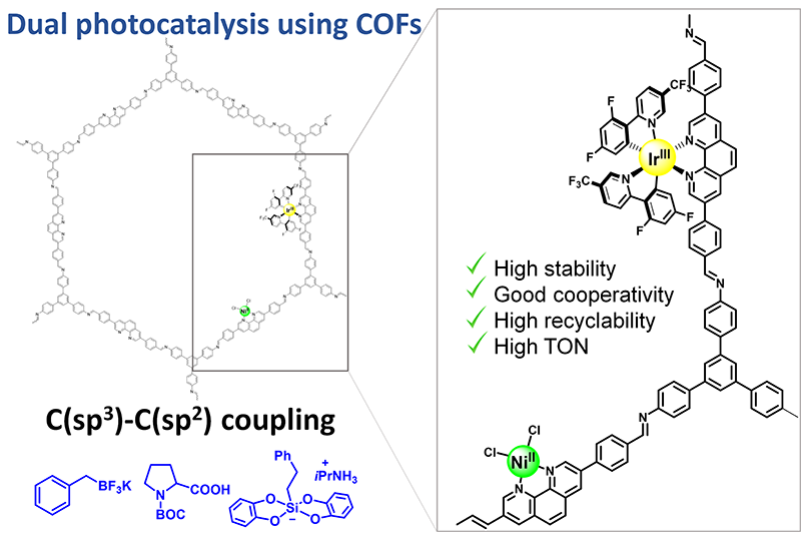

The functionalization of an imine-based layered covalent organic framework (COF), containing phenanthroline units as ligands, has allowed the obtention of a heterobimetallated material. Photoactive Ir and Ni fragments were immobilized within the porous structure of the COF, enabling heterogeneous light-mediated Csp^3^–Csp^2^ cross-couplings. As radical precursors, potassium benzyl- and alkoxy-trifluoroborates, organic silicates, and proline derivatives were employed, which brings out the good versatility of **Ir,Ni@Phen-COF**. Moreover, in all the studied cases, an enhanced activity and stability have been observed in comparison with analogous homogenous systems.

## 1 Introduction

**Figure 1:**
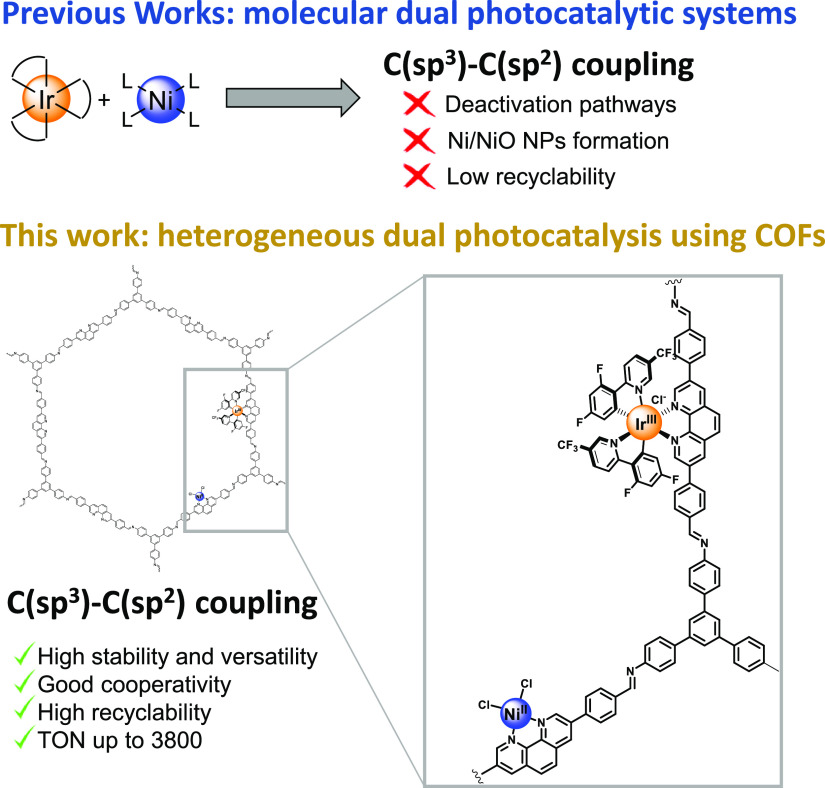
Previous works and the conceptual strategy of this work.

Carbon–carbon couplings are one of the most useful and important transformations in modern chemistry.^1^ They include classical cross-coupling reactions,^2^ such as Suzuki, Sonogashira, and olefin metathesis;^3^ some of them recognized with the Nobel prize.^4^ The importance of these reactions relies on their key role in the synthesis of pharmaceutical drugs, construction of complex molecules, and late stage functionalizations. However, there is still a need of searching new synthetic methodologies with high functional group tolerance, in which are included new Csp^3^–Csp^2^ bonds. In this sense, photocatalytic reactions have appeared as a new tool in the last decade, solving some of the problems associated with the formation of C–C bonds under mild reaction conditions.^5^ Among them, photoredox Ir–Ni dual catalysis has become an outstanding solution (top, Figure 1).^5^ In this reaction, two well-distinguished and connected catalytic cycles are combined: first, the Ir photocatalyst generates a radical intermediate under light irradiation; and second, the Ni species mediates in the formation of the new C–C bond.^6,7^ Although this is a very powerful methodology, Ir–Ni dual photocatalysis also suffers of some general drawbacks. First, iridium is an expensive metal center and its recyclability in the homogeneous system is difficult,^8^ increasing the costs of these processes. Furthermore, Ni sources commonly undergo deactivation in the presence of light, oxygen, or organic reactive species, giving rise to the formation of unreactive agglomerated Ni/NiO nanoparticles. This hampers the furtherance of the reaction, leading to lower yields and turnover numbers (TONs).^9^ Therefore, a plausible strategy to overcome the limitations of homogenous Ir–Ni dual catalysis would consist of the design of heterogeneous systems.

The use of catalytic heterogeneous materials allows their easy separation from the reaction medium. In addition, the immobilization and isolation of catalytically active sites may enhance their stability and reactivity, due to the hampering of unproductive encounters or deactivation pathways,^10^ such as the formation of inactive agglomerated Ni/NiO nanoparticles.^9^ Besides, cooperative phenomena between distinct isolated catalytic centers can be enhanced by the control of proximity in predetermined sites on the material. To this end, it would be a feasible strategy to immobilize molecular metallic catalytic sites into porous materials, such as covalent organic frameworks (COFs).^11,12^ This family of materials is in its heyday on their catalytic applications.^13^ They constitute a class of crystalline and porous reticular materials formed by the assembly of covalent bonds of different nature.^14^ Among them, imine-based COFs have attracted a good deal of attention, due to their stability, availability of the constituting building blocks, and the easy isolation of crystalline and porous structures under mild reaction conditions.^15,16^ The accessibility to a variety of building blocks allows the design of COFs containing different functional units in their framework.^17^ In particular, some COFs have been isolated containing donor groups (e.g., bipyridine,^18^ salen,^19^ porphyrin,^20^ or catechol,^21^ among others)^19,22−24^ that can act as ligands toward a variety of metal centers. As a result, different organic transformations have been achieved with a variety of metals.^17,22,23,25^ However, dual bimetallic photocatalytic processes are a challenging goal that never have been tackled with this family of materials. In particular, dual catalytic systems require a high level of synchronicity between the two catalytic sites, because the intermediates involved in such processes are very reactive and not isolable. The proximity and cooperativity between the distinct catalytic sites are a key issue in the design of a dual catalytic process, and therefore catalytic sites must work in close proximity.^26^

Regarding this issue, two pioneering works using metal organic frameworks (MOFs) have reported the isolation of Ni and Ir centers through coordination to bipyridine fragments, achieving dual photocatalytic processes.^27,28^ In addition, non-porous amorphous linear polymers designed with pending arms containing bipyridine fragments have also been used for these purposes.^29^ Inspired by these precedents, this work is intended to design a bimetallated COF that requires simple synthetic procedures and to obtain a very stable and active material. In particular, we immobilize Ir and Ni fragments into phenanthroline units in a new imine-based COF (hereafter called **Phen-COF**), allowing the interplay of both catalytic metal centers for the formation of Csp^3^–Csp^2^ bonds (bottom, Figure 1). This heterogeneous strategy permits the improvement of the catalytic performance compared with the homogeneous version of the coupling reaction.

## 2 Results and Discussion

### 2.1 Synthesis and Characterization

**Figure 2:**
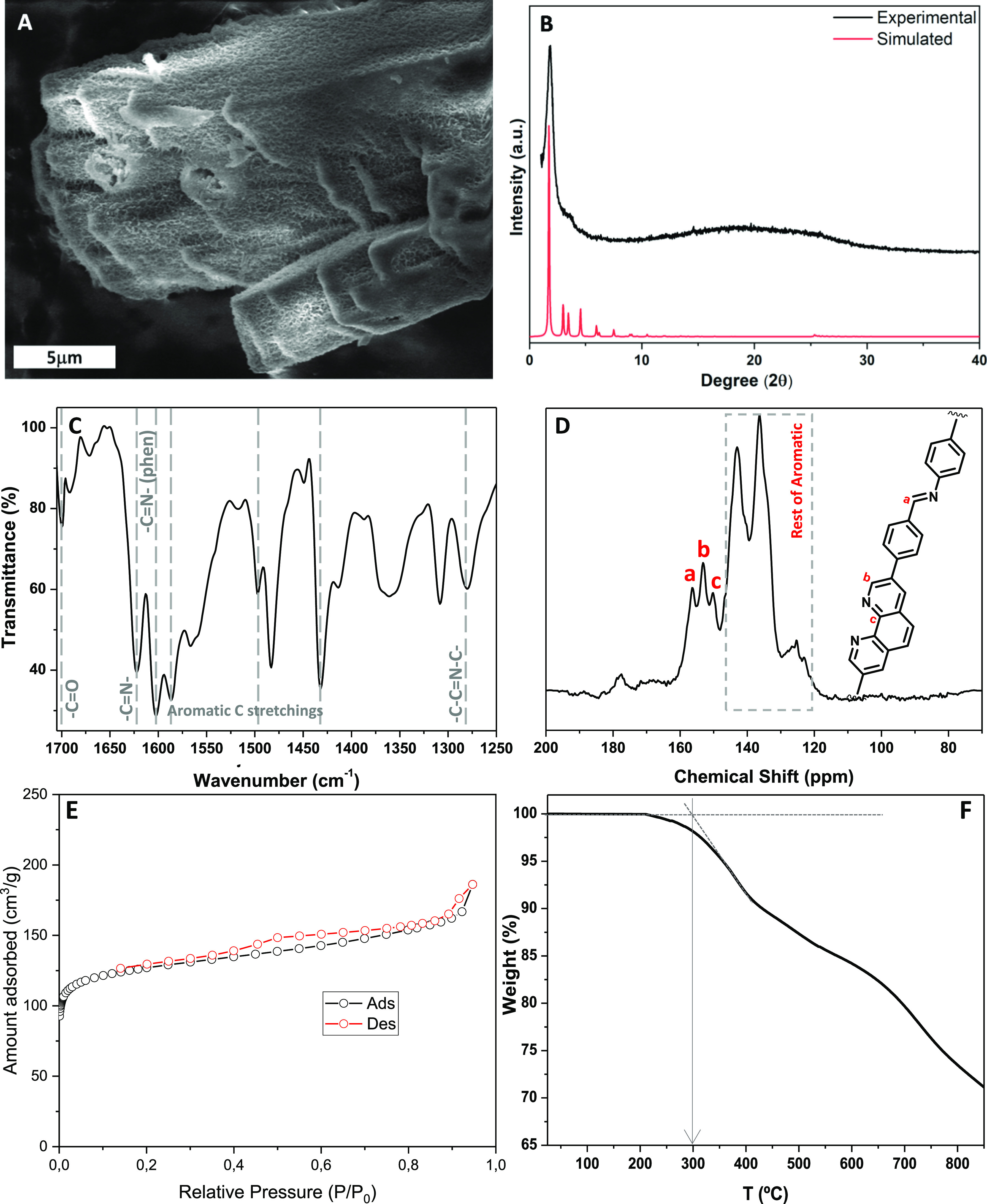
Characterization of **Phen-COF**. (A) SEM image; (B) PXRD simulated and experimental pattern; (C) FT-IR spectra; (D) ^13^C NMR-CPMAS spectrum; (E) adsorption–desorption N_2_ isotherm; and (F) thermogravimetric analysis.

**Scheme 1:**
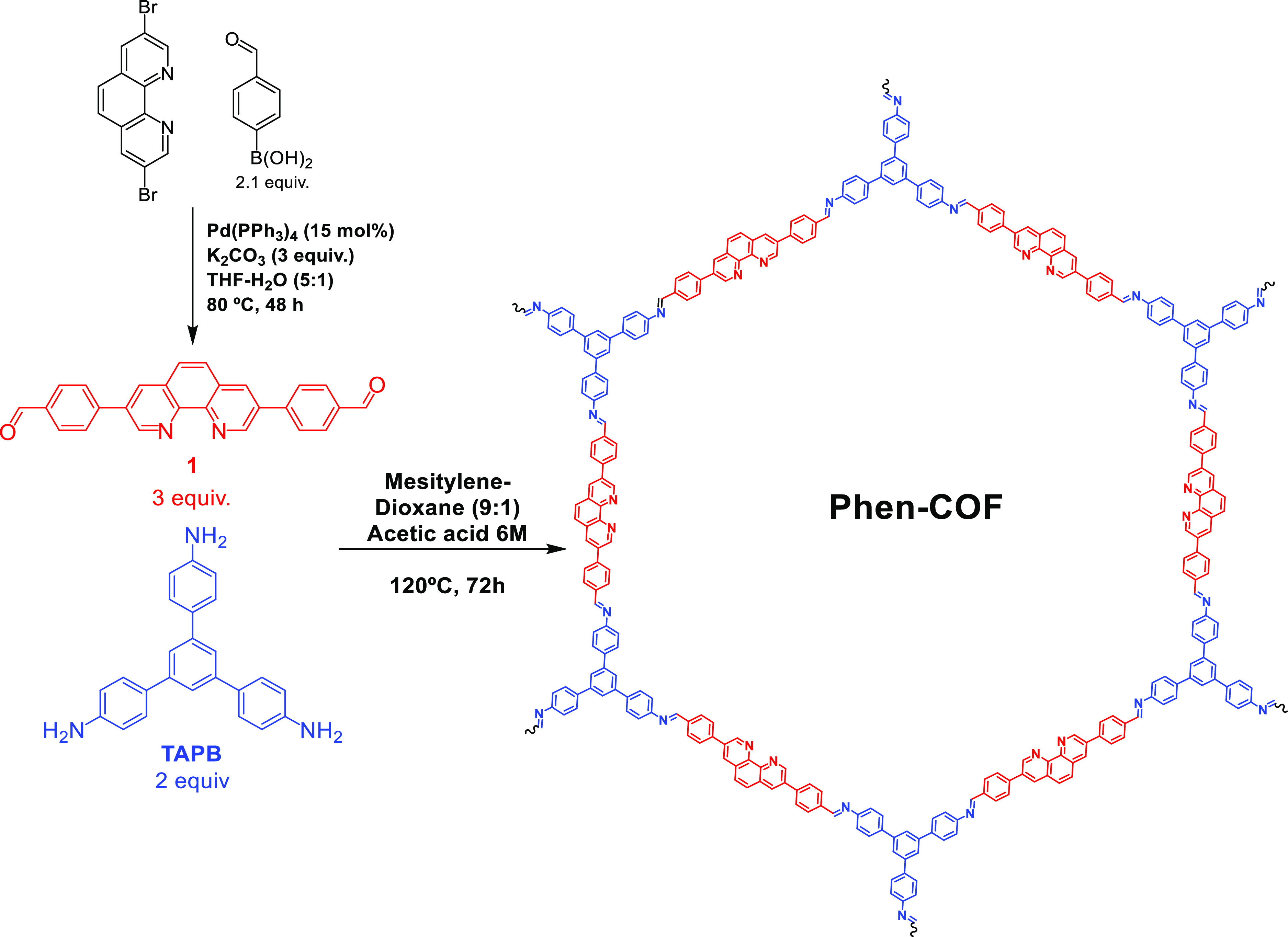
Synthesis of **Phen-COF**

**Phen-COF** was prepared by combining the symmetric building block **1**, which contains a centered phenanthroline unit and two aldehyde groups located at its ends, and the well-known triamine 1,3,5-tris(4-aminophenyl)benzene (TAPB) (see Scheme 1).^12^ First, the dialdehyde **1** was made by a double Suzuki–Miyaura cross-coupling between 3,8-dibromo-1,10-phenanthroline and 4-formylphenylboronic acid. Then, a screening of typical conditions for the synthesis of **Phen-COF** was performed. In most of the cases, a yellow amorphous material was obtained (see Table S1 of Supporting Information), probably because of the poor solubility of phenanthroline building block **1**. Interestingly, using a mixture of 9:1 (v/v) of mesitylene-dioxane and 6 M acetic acid on a sealed solvothermal reactor, we were able to isolate a material with moderate crystallinity after 3 days at 120 °C (entry 15, Table S1). Longer reaction times, higher temperatures, or the use of acid catalysts of different nature did not improve the crystallinity of the material, not even post-thermal annealing treatments or activation with supercritical CO_2_. Field-emission scanning electron microscopy (FE-SEM) of the resulting yellow powder revealed the formation of a layered-type COF (Figure 2A). The formation of the expected layered polyimine structure of **Phen-COF** (Scheme 1) was first confirmed by powder X-ray diffraction (PXRD) (Figure 2B). Using the Materials Studio 8.0 Program, we compared this experimental PXRD data with the pattern simulated from the analogous layered, eclipsed structure of PI-COF-3 previously reported by Yan et al.^30^ Based on this structure, we applied a geometrical energy minimization using the universal force field. The unit cell parameters found for the layered eclipsed structure were *a* = 3.52 Å, *b* = 102.59 Å, and *c* = 59.23 Å in an *Amm*2 (no. 38) symmetry group. Consistently, the most intense peak at 1.73° of our experimental PXRD matched with the peak corresponding to the (100) plane in the eclipsed pattern simulated from the COF model (Figure 2B).

Moreover, Fourier-transform infrared spectroscopy (FT-IR) (Figures 2C and S1 of Supporting Information) showed the most important vibration peaks at 1621 and 1282 cm^–1^ (attributed to the C=N and C–C=N–C stretching of imine moieties), indicating the formation of the polyimine network.^10^ In addition, the presence of phenanthroline fragments in this network was corroborated by the vibration found at 1603 cm^–1^, also observed in **1**, which was attributed to the C=N stretching. The vibration peak at 1697 cm^–1^ was assigned to the C=O stretching of unreacted aldehydes, which agrees with the appearance of Fermi resonance peaks at 2919 and 2850 cm^–1^. Moreover, the two peaks at 3349 and 3418 cm^–1^ are related to the symmetric and asymmetric N–H stretchings of the free amino groups. The corresponding C–C aromatic ring stretchings at 1588, 1497, and 1432 cm^–1^ and C_aromatic_–H stretchings centered at 3027 cm^–1^ are also important in typical polyaromatic networks that contain **TAPB** as the building block.^10,12^ In addition, solid-^13^C NMR experiments using Cross Polarization combined to Magic Angle Spinning (^13^C-CP-MAS) showed the characteristic iminic carbon peak at 156.4 ppm as well as the peaks centered at 153.2 and 150.1 ppm, which were assigned to the tertiary and quaternary carbons in the alpha position to the phenanthroline-nitrogen atom (Figure 2D. For the comparison with the ^13^C-CP-MAS spectrum of **1**, see Figure S4 of Supporting Information). The rest of the signals correspond to several aromatic carbons present in the covalent structure. These results match with those observed in comparable pristine layered-COFs.^10^ As indicated in Figure 2E, N_2_ sorption analysis at 77 K showed that **Phen-COF** is porous with a Brunauer–Emmett–Teller surface area (*S*_BET_) of 482 m^2^/g. The pore size distribution was calculated, finding pores of 13.4 and 15.6 Å (Figure S7 of Supporting Information). Finally, thermogravimetric analysis (TGA) showed that **Phen-COF** possesses good thermal stability, up to 300 °C even under an oxidant atmosphere (Figure 2F).

**Figure 3:**
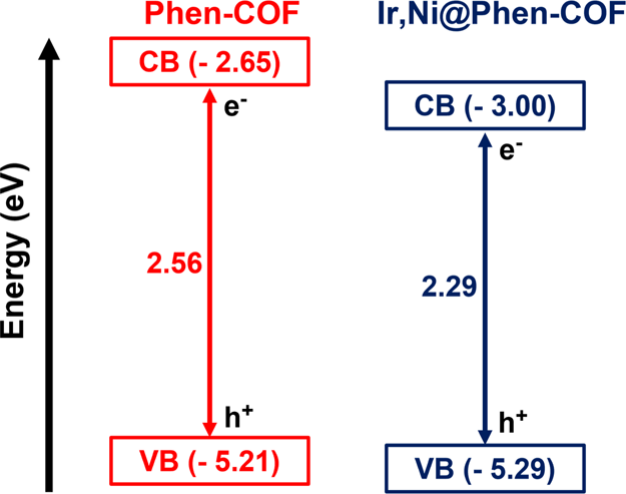
Energy levels of **Phen-COF** and **Ir.Ni@Phen-COF**. The values of the VB were calculated from the first oxidation signal of the cyclic voltammetry curve. Band gap values were obtained from the DRS spectra and subsequent application of Kubelka–Munk theory.

In order to study the optical properties of **Phen-COF**, diffuse reflectance spectroscopy (DRS) was performed, revealing a continuous intense absorption of the material up to 450 nm (Figure S8 of Supporting Information). Applying Kubelka–Munk theory, the band gap of **Phen-COF** was found to be around 2.56 eV, which is comparable to that of other pristine layered imine-based COFs.^12^ Additionally, fluorescence emission spectroscopy of **Phen-COF** showed broad emission bands with maximums centered at 470 and 537 nm, respectively (Figure S9 of Supporting Information). Electrochemical properties were determined by cyclic voltammetry measurements (Figure S10 of Supporting Information). One reduction and one oxidation process were observed at −0.10 and +0.82 V (vs Ag/AgCl), respectively. Reduction processes are expected due to the presence of phenanthroline, which is known as redox active at negative potentials (Figure S10, Supporting Information). The oxidation signal is attributed to the electron transfer from the valence band (VB) of the material, which leads to an energy value determined as 5.21 eV (see Figure 3 for determination of energy levels).

### 2.2 Postsynthetic Metalation

Postsynthetic metalation of **Phen-COF** consisted of dispersing it in acetonitrile and 6 M aqueous acetic acid in the presence of [(dF(CF_3_)ppy)_2_-Ir-μ-Cl]_2_ and NiCl_2_·glyme (see Supporting Information for further details). For this metalation, [(dF(CF_3_)ppy)_2_-Ir-μ-Cl]_2_ was chosen as the iridium source^31^ because it would enable the incorporation of a powerful photoredox complex through coordination with the phenanthroline ligands contained in **Phen-COF**.^6^ NiCl_2_·glyme was selected as the Ni source due to the ability of glyme ligands to undergo exchange with phenanthroline moieties. Note that acetic acid was used in the metalation process because it was essential to preserve the crystallinity of **Phen-COF**,^32^ as its absence always led to amorphization of the structure.

The content of Ir and Ni incorporated into the metallated **Phen-COF** (hereafter called **Ir,Ni@Phen-COF**) was analyzed by total X-ray fluorescence (TXRF, see Supporting Information, Figure S17).^33^ We found that the Ir and Ni contents in **Ir,Ni@Phen-COF** are 4.0 and 2.8% in weight, respectively. These values mean that 45% of the phenanthroline ligands present in **Phen-COF** coordinates to metals: 15% to Ir and 30% to Ni. Interestingly, the metal uptake from the solution is quite efficient. In fact, the 71% of the Ir species in solution is incorporated into the material, while the 82% of the Ni precursor is bound to the framework. Thus, the Ir/Ni ratio in the precursor’s solution is kept in the postfunctionalized material. Moreover, FT-IR, ^13^C NMR-CPMAS, PXRD, TGA, and FE-SEM performed on **Ir,Ni@Phen-COF** did not show any significant differences between the pristine and metallated **Phen-COF** (see Supporting Information).

The energy-dispersive X-ray (SEM–EDX) mapping images showed the presence of homogeneously distributed C, N, Ir, Ni, Cl, and F atoms in the framework (see Figure S16 in Supporting Information). This result is in accordance with the X-ray photoelectron spectroscopy (XPS) analysis results of the bimetallated material (Figures S24 and S25 of Supporting Information). The binding energy of the Ni 2p band at 855.9 eV corresponds to Ni(II) species, which is close to that of the NiCl_2_(bpy) complex,^26,34^ suggesting the coordination of Ni to the phenanthroline ligands present in the framework. The two peaks located at 62.2 (Ir 4f_7/2_) and 65.3 (Ir 4f_5/2_) eV are indicative of octahedral Ir(III) polypyridyl species coordinated to both C,N and N,N bidentate ligands,^35,36^ which is consistent with the proposed expected coordination of Ir complexes to the phenanthroline ligands of the framework, as can be seen in Figure 1. The peak at 688 eV is assigned to fluorine (F 1s) present in the ligands of the Ir complex.^35^ Furthermore, the N 1s signal centered at 399.1 eV is similar to the observed features for related materials containing pyridyl and iminic nitrogen atoms reported in the literature.^37^ Interestingly, the presence of Ir and Ni centers has a significant impact on the N 1s XPS features. While the signals observed in the pristine material are conserved, also electrons at lower energies are detected (Figure S26 of Supporting Information). According to previous reports,^37^ pyridinic N atoms are observed at lower energies than the iminic ones. Therefore, this observation indicates that N-phenanthroline atoms are affected by the presence of metal centers. Thus, these data are consistent with the coordination of phenanthroline units to Ni and Ir fragments.

Transmission electron microscopy (TEM) of **Ir,Ni@Phen-COF** confirmed the absence of inorganic nanoparticles (see Supporting Information, Figure S44, left). **Ir,Ni@Phen-COF** conserved the porosity with a *S*_BET_ of 432 m^2^/g (see Supporting Information, Figure S17). The pore size distribution analysis showed values of 13.4 and 15.6 Å (Figure S18).

With respect to the optical properties of **Ir,Ni@Phen-COF**, DRS unveiled a slight increase of the visible absorption of this material in comparison with the pristine one (Figure S20, Supporting Information). This observation is translated into a decrease in the band gap of the material, which changes from 2.56 eV in the pristine material to 2.29 eV in the metallated one. Moreover, the fluorescence emission spectra showed again a broad emission band at the same wavelengths than that observed for the pristine material. However, also a sharp emission at 442 nm becomes the maximum intensity signal in the spectrum (Figure S21). Furthermore, electrochemical features of the organic material mask the expected signals for the metal centers (Figure S22). As a consequence, no significant differences between pristine and metallated materials are observed by cyclic voltammetry, and the valence band value was determined to be −5.29 eV (see Figure 3).^38^ Considering that only the bimetallated material is active in the catalytic process (see below), it is worth noting that properties determined by electrochemical measurements are not relevant for such activity.

The role of phenanthroline was furtherly proved by comparing the metal uptake of **Phen-COF** with that of a well-known layered imine-based COF without coordinating units. This imine-based COF has been previously studied in the incorporation of metal centers for catalysis, such as Pd(II).^12^ These materials (**Phen-COF** and **Crystalline Laminar**-**COF**) were incubated under the same specific reaction conditions in the presence of Ir and Ni metal precursors. Remarkably, very low quantities of both metal centers were found after TXRF analyses (<0.1%), using the COF without phenanthroline fragments (see Supporting Information, Figure S40). It is worth noting that the procedure used in these essays includes an exhaustive overnight washing by Soxhlet extraction. This treatment can wash out molecular metallic species that could interact weakly with the COF structure as a result of supramolecular interactions^39^ or through coordination to the iminic nitrogens.^40^ Therefore, strong metal–phenanthroline interactions resulting from the chelating effect stabilizes the metalation of the COF structure, even after exhaustive washing. As a consequence, a robust heterogeneous catalytic system is achieved by using **Phen-COF** as a porous platform, in which the metallic sites will be permanently immobilized over the whole catalytic procedure.

Additionally, this metalation process was repeated forming the mono-metallated **Ni@Phen-COF** and **Ir@Phen-COF** with a Ir and Ni loading of 3.9 and 2.9% in weight, respectively (see Supporting Information, Figures S31 and S37). Remarkably, these values are similar to those found for **Ir,Ni@Phen-COF**, making them good materials to be used as control in catalytic experiments for comparison purposes (*vide infra*).

### 2.3 Catalytic Activity

The catalytic performance of **Ir,Ni@Phen-COF** was initially evaluated studying as a model reaction the light-mediated cross-coupling between potassium benzyltrifluoroborate and 4-bromotoluene, which was reported using an homogeneous catalytic system by Molander and co-workers.^7^ To our delight, bimetallated **Ir,Ni@Phen-COF** effectively catalyzed this reaction, reaching 90% conversion under blue light irradiation (Table 1, entry 1; see optimization in Table S2 of Supporting Information). As control experiments, we evaluated the performance of the pristine **Phen-COF** (Table 1, entry 2). In addition, we also carried out the reaction in the absence of any catalyst or under dark conditions (Table 1, entries 3 and 4). In the three cases, we observed negligible conversion. Moreover, the use of the **Post-Functionalized-Crystalline Laminar-COF** with an extremely low content of Ni showed no conversion (Table 1, entry 5). In order to disclose the effect of having a heterogeneous system that included both Ni and Ir centers in the same material, we tried the reaction using the mono-metallated **Ni@Phen-COF** or **Ir@Phen-COF**, observing zero conversion in both cases (Table 1, entries 6 and 7). In the case of **Ni@Phen-COF**, it is worth mentioning that no product formation was observed, due to the lack of the photocatalytic unit. Therefore, the Ir center is required to generate the desired product. Nevertheless, when **Ni@Phen-COF** and **Ir@Phen-COF** were mixed in the same reaction medium, the photocatalytic process did not result in any product either (Table 1, entry 8). These observations are indicative of the requirements for the appropriate cooperativity in dual heterogeneous photocatalysis. In fact, these results account for a mechanism implying very reactive radical intermediates, which is characteristic of dual catalysis. Otherwise, in the reported molecular Ir/Ni dual catalysis in solution, their interplay depends on concentration and diffusion of active intermediates and catalysts. Thus, despite interesting activities have been reported in homogenous systems, the variable proximity in solution of catalytic sites can limits their efficiency. Design of heterobimetallic materials fixes the proximity between Ir and Ni centers, optimizing their cooperativity, which represents an advantage of the heterogeneous system with respect to the homogeneous one.

**Table 1:**
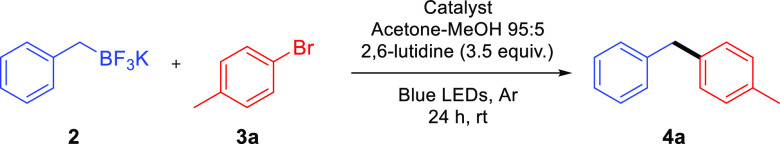
Experiments for the Light-Mediated Cross-Coupling between Potassium Benzyltrifluoroborates and Aryl Bromides^a^

entry	catalyst	yield (%)^b^	TON (Ir/Ni)
1	Ir**,Ni@Phen-COF** (1.2 mg, 4.0% Ir, 2.8% Ni)	90	270/118
2	**Phen-COF** (1.2 mg)	0	
3	No Catalyst	0	
4	Ir**,Ni@Phen-COF** (1.2 mg) (dark)	0	
5	**PF–CrystallineLaminar COF** (1.2 mg)	0	
6	Ni@**Phen-COF** (1.2 mg)	0	
7	Ir@**Phen-COF** (1.2 mg)	0	
8	Ni@**Phen-COF** (0.6 mg)		
	Ir@**Phen-COF** (0.6 mg)	0	
9	[Ir(dF(CF_3_)ppy)_2_(bpy)]PF_6_ (0.3 mol %) [NiCl_2_(phen)] (0.7 mol %)	23	69/30
10	**Ir,Ni@Phen-COF** (1.2 mg, 7.0% Ir, 4.2% Ni)	87	149/76
11	**Ir,Ni@Phen-COF** (1.2 mg, 2.0% Ir, 1.4% Ni)	25	150/66
12	**Ir,Ni@Phen-COF** (1.2 mg, 2.2% Ir, 2.8% Ni)	42	229/55
13	**Ir,Ni@Phen-COF** (1.2 mg, 9.0% Ir, 2.8% Ni)	46	61/60

a The reaction was carried out using **2a** (0.15 mmol), **3a** (0.075 mmol), 2,6-lutidine (0.26 mmol), the corresponding catalyst, and 1 mL of solvent under an Ar atmosphere and blue light irradiation (blue LEDs, 23 W, 450 nm).

b The yield was determined by ^1^H NMR using nitromethane as the internal standard.

To assess the advantage of the bimetallated COF *versus* a mixture of comparable homogeneous catalysts, we tested the performance of the molecular version of our catalytic system. To this end, we studied the reaction using as catalysts [Ir(dF(CF_3_)ppy)_2_(bpy)]PF_6_ and [NiCl_2_(phen)] in the catalytic loadings analogous to the Ni and Ir contents in **Ir,Ni@Phen-COF** (0.7 and 0.3 mol %, respectively). Remarkably, when the homogeneously catalyzed reaction was performed, the reaction reached 23% conversion (Table 1, entry 9), while almost full conversion was achieved using the bimetallated material under comparable conditions. This observation points out the limitation of the homogeneous system, which is probably due to the formation of unreactive aggregated Ni/NiO particles.^9^ Accordingly, after one catalytic run, it was observed that a dark solid is formed. Finally, we also studied the influence of metal-loading on the catalytic outcome. For this purpose, maintaining the 2:1 molar ratio between Ni and Ir centers, we obtained two other different materials: one by doubling and the other by halving the amount of metal precursors employed in the postfunctionalization stage. Under the same conditions, both materials did not improve the yields observed for the initial **Ir,Ni@Phen-COF** (see Table 1, entries 10 and 11, and Table S3 of Supporting Information). The optimal Ni/Ir ratio found was 2:1 because other proportions (such as 4:1 and 1:1) gave worse yields and TONs (see Table 1, entries 12 and 13, and Table S3 of Supporting Information).

**Scheme 2:**
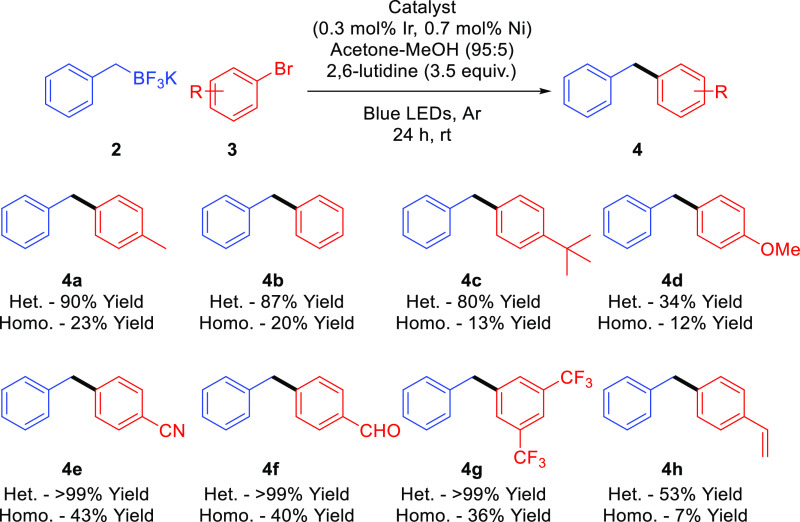
Scope of Light-Mediated Cross-Coupling between Potassium Benzyltrifluoroborates and Aryl Bromides The heterogeneously catalyzed reaction (Het.) was performed using **Ir,Ni@Phen-COF** as the catalyst. The homogeneously catalyzed reaction (Homo.) was performed using [Ir(dF(CF3)ppy)_2_(bpy)]PF_6_ and [NiCl_2_(phen)] as catalysts. The yields were determined by ^1^H NMR using nitromethane as the internal standard

The applicability of our heterogeneous system has been also evaluated for a variety of aryl bromides, allowing not only the introduction of several electron withdrawing or electron donating groups, but also more hindered or even with olefinic substituents. Thus, eight different unsymmetrical diarylmethanes were isolated from moderate to good yields applying this heterogeneous system. In all the studied cases, the observed catalytic activity for **Ir,Ni@Phen-COF** was, at least, double that the observed for the homogenously catalyzed version (Scheme 2).

In order to further evaluate the scope of this dual heterogeneous catalytic system, we explored the use of other radical precursors of different nature. Therefore, we decided to substitute potassium benzyltrifluoroborate by *tert*-butoxytrifluoroborate, from which radical formation is thermodynamically slightly less favored.^41^ Under the same reaction conditions, the photocatalytic cross-coupling between this substrate and 4-bromobenzonitrile was effectively performed, obtaining 76% of yield of product 6 (Table 2, entry 1). However, the homogeneously catalyzed reaction only achieved 12% of yield under the same reaction conditions (Table 2, entry 8). For this reaction, it was impossible to reach any conversion when the reaction was carried out in absence of a catalyst, or by the monometallic functionalized COFs, or when both monometallic materials were mixed in the same reaction medium (Table 2, entries 3 to 7).

**Table 2:**
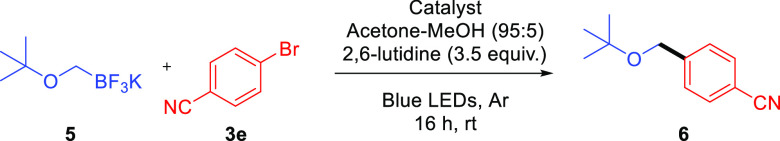
Experiments for the Light-Mediated Cross-Coupling between Potassium *tert*-Butyltrifluoroborate and Aryl Bromides^a^

entry	catalyst	NMR yield (%)^b^
1	**Ir,Ni@Phen-COF** (1.2 mg)	76
2	**Phen-COF** (1.2 mg)	0
3	No Catalyst	0
4	**Ir,Ni@Phen-COF** (1.2 mg) (dark)	0
5	**Ni@Phen-COF** (1.2 mg)	0
6	**Ir@Phen-COF** (1.2 mg)	0
7	**Ni@Phen-COF** (0.6 mg)	
	**Ir@Phen-COF** (0.6 mg)	0
8	[Ir(dF(CF_3_)ppy)_2_(bpy)]PF_6_ (0.3 mol %) [NiCl_2_(phen)] (0.7 mol %)	12

a The reaction was carried out using **5** (0.15 mmol), **3e** (0.075 mmol), 2,6-lutidine (0.26 mmol), the corresponding catalyst, and 1 mL of solvent under an Ar atmosphere and blue light irradiation (blue LEDs, 450 nm).

b The yield was determined by ^1^H NMR using nitromethane as the internal standard.

Another interesting type of reagents that can trigger photo-mediated cross-couplings are organic silicates. These substrates can offer some advantages in comparison with organic potassium trifluoroborates, such as avoiding byproducts, better solubility, and easier generation of unstabilized primary radicals because their redox potential is lower.^42^ Therefore, we performed the light-mediated cross-coupling between organic silicate **7** and 4-bromobenzonitrile under typical reaction conditions and using **Ir,Ni@Phen-COF** as a heterogeneous photocatalyst. As a result, the reaction reached 92% of yield after 14 h of irradiation at room temperature (Table 3, entry 1). Control experiments showed again that proximity and cooperativity between immobilized Ir and Ni complexes into **Phen-COF** were indispensable for triggering this organic transformation (Table 3, entries 2 to 7). In addition, the homogeneously catalyzed reaction only gave 26% under analogous conditions (Table 3, entry 8).

**Table 3:**
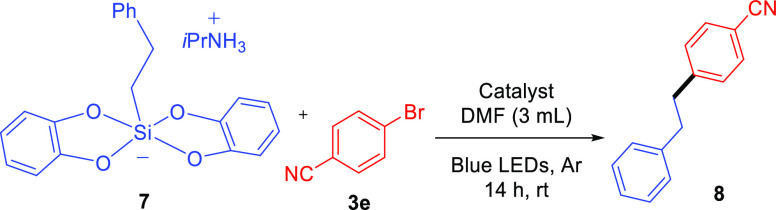
Control Experiments for the Light-Mediated Cross-Coupling between Organic Silicates and Aryl Bromides^a^

entry	catalyst	NMR yield (%)^b^
1	**Ir,Ni@Phen-COF** (1.2 mg)	92
2	**Phen-COF** (1.2 mg)	0
3	No Catalyst	0
4	**Ir,Ni@Phen-COF** (1.2 mg) (dark)	0
5	**Ni@Phen-COF** (1.2 mg)	0
6	**Ir@Phen-COF** (1.2 mg)	0
7	**Ni@Phen-COF** (0.6 mg)	
	**Ir@Phen-COF** (0.6 mg)	0
8	[Ir(dF(CF_3_)ppy)_2_(bpy)]PF_6_ (0.3 mol %)	
	[NiCl_2_(phen)] (0.7 mol %)	26

a The reaction was carried out using **7** (0.225 mmol), **3e** (0.15 mmol), the corresponding catalyst, and 3 mL of solvent under an Ar atmosphere and blue light irradiation (blue LEDs, 450 nm).

b The yield was determined by ^1^H NMR using nitromethane as the internal standard.

Alternatively, proline derivatives can also be used as radical precursors. The photocatalytic decarboxylative arylation of alkyl carboxylic acids is interesting from the chemical point of view, because of the easy accessibility to these radical precursors and the potential interest of heterocyclic chemistry.^43^ Thus, we performed the photocatalytic cross-coupling between *N*-protected proline **9** and 4-bromobenzonitrile under reaction conditions previously reported for a homogeneous system (see Supporting Information for further details).^43^ In this case, acetonitrile was employed as a solvent and cesium carbonate as a base to trigger the deprotonation of the corresponding α-amino acid derivative. In this case, the reaction catalyzed by **Ir,Ni@Phen-COF** reached 86% of isolated yield of the corresponding cross-coupled product **10** (Table 4, entry 1), while the homogeneous version was stalled at 58% (Table 4, entry 8). No background (Table 4, entries 2 and 3) or monometallic-catalyzed reactions (Table 4, entries 5 and 6) gave rise to the desired product, neither when both monometallic-functionalized COFs were reunited in the same reaction container (Table 4, entry 7).

**Table 4:**
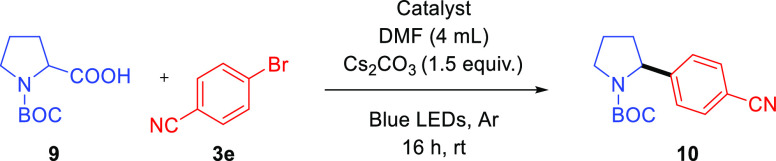
Control Experiments for the Light-Mediated Decarboxylative Arylation of Alkyl Carboxylic Acids.^a^

entry	catalyst	NMR yield (%)^b^
1	**Ir,Ni@Phen-COF** (1.2 mg)	86
2	**Phen-COF** (1.2 mg)	0
3	No Catalyst	0
4	**Ir,Ni@Phen-COF** (1.2 mg) (dark)	0
5	**Ni@Phen-COF** (1.2 mg)	0
6	**Ir@Phen-COF** (1.2 mg)	0
7	**Ni@Phen-COF** (0.6 mg)	
	**Ir@Phen-COF** (0.6 mg)	0
8	[Ir(dF(CF_3_)ppy)_2_(bpy)]PF_6_ (0.3 mol %) [NiCl_2_(phen)] (0.7 mol %)	58

a The reaction was carried out using **6** (0.225 mmol), **3e** (0.15 mmol), caesium carbonate (0.225 mmol), the corresponding catalyst, and 4 mL of solvent under an Ar atmosphere and blue light irradiation (blue LEDs, 450 nm).

b The yield was determined by ^1^H NMR using nitromethane as the internal standard.

The main observation of this work is that a heterogeneous system has a significantly higher catalytic performance than the analogous homogeneous counterparts. From the data found in the literature,^7,41−43^ it can be concluded that the homogeneous system used in this work shows comparable activities to the previously published systems (see Table S3, Supporting Information). Furthermore, our heterogeneous **Ir,Ni@Phen-COF** catalyst allowed achieving significantly higher TONs than any of the homogeneous catalytic versions.

### 2.4 Leaching Tests and Recyclability

An important requirement of heterogeneous functionalized materials consists of avoiding the leaching processes of molecular active fragments into the reaction solution.^44^ This is important because one of the most interesting advantages of heterogeneous catalysis is the easy separation of the catalyst (by a simple filtration) preventing the release of any spurious species into the pure fractions of products. In our case, under the experimental conditions of the four studied reactions, we discarded leaching processes through filtration of the catalyst after one catalytic run and analysis of the supernatant by ICP measurements. Analytical data revealed that no metal species were found in the solution after simple filtration of the catalyst. In addition, when the filtered solution was used in the presence of additional amounts of the different starting materials, we found no reactivity at all, indicating that no catalytic species were leached into the reaction media.

**Figure 4:**
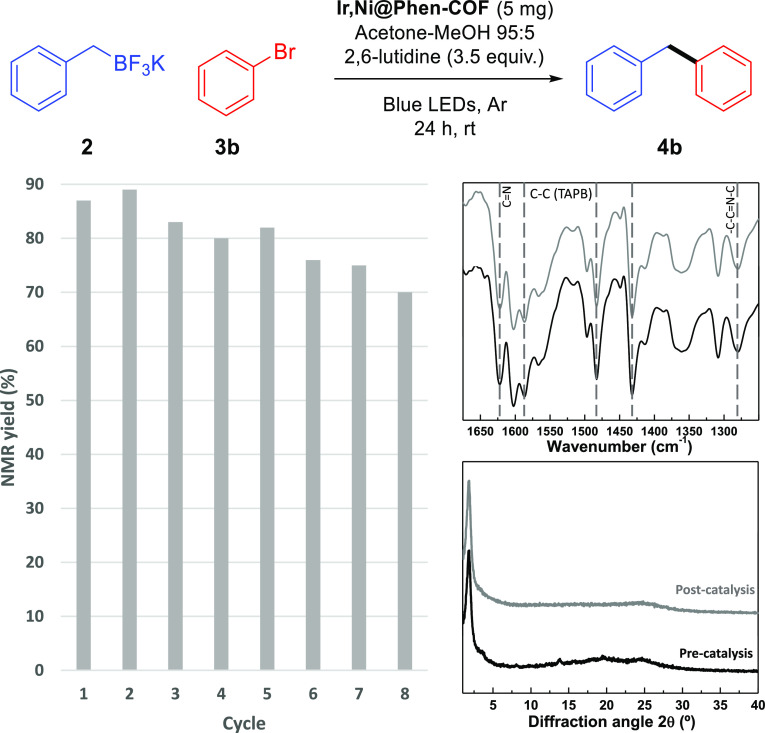
Recycling of **Ir,Ni@Phen-COF** as a heterogeneous photocatalyst for light-mediated cross-coupling of potassium benzyltrifluoroborates and bromobenzene.

Moreover, to prove the recyclability of our catalyst, **Ir,Ni@Phen-COF** was recovered by centrifugation, confirming the preservation of its chemical identity by FT-IR (Figure 4). Crystallinity was retrieved after simple solvothermal treatment of the material (Figure 4). In addition, TEM reveals that nanoparticles were not formed during the catalytic process (see Supporting Information, Figure S42). Moreover, this material can be used for eight consecutive catalytic cycles with minor loss of activity, generating 1 mmol (168 mg) of diphenylmethane **4b** using 5 mg of **Ir,Ni@Phen-COF** overall (Figure 4). In terms of TON, this catalyst showed after the eight runs 925 TON for Ir and 424 TON for Ni, confirming its sturdiness and recyclability. Finally, as a proof for the scalability of this system, we performed this reaction at 5 mmol scale, maintaining the catalyst loading at 5 mg. A yield of 79% was reached after 96 h of irradiation, which implies 3800 TON for Ir and 1650 TON for Ni.

### 2.5 Mechanistic Considerations

**Figure 5:**
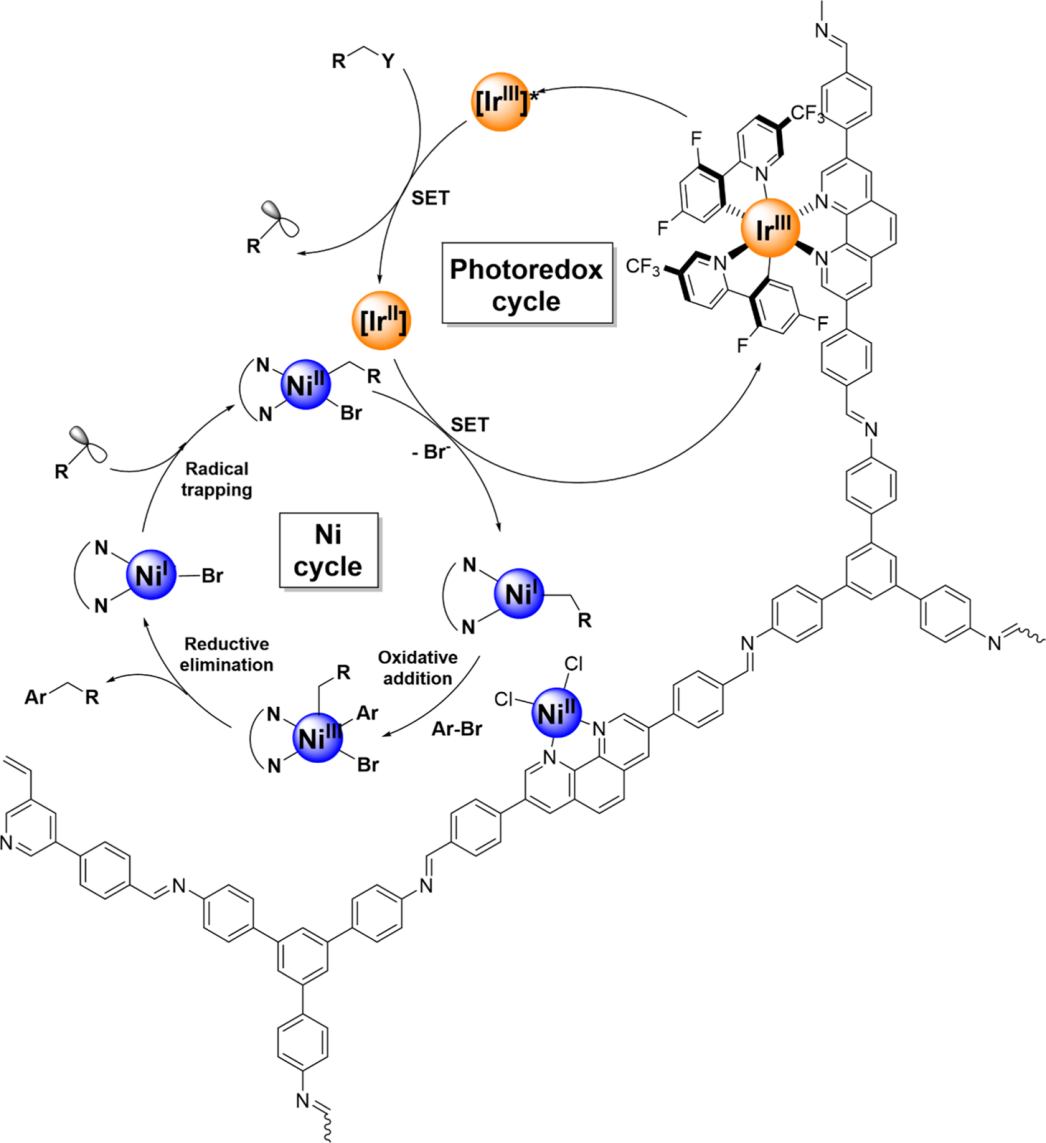
Mechanism of the transformations carried out in this work using **Ir,Ni@Phen-COF** as a heterogeneous dual catalytic system.

The mechanism for dual catalytic systems closely related to this work has been previously considered using homogeneous species^7^ and MOFs with similar coordinating units (Figure 5).^27^ According to the available data, it is well established that Ir(III) species in the excited state is able to oxidize the radical precursor, which generates paramagnetic species that binds to a Ni(I) center through radical trapping. The corresponding Ni(II) center is reduced by the transient Ir(II) intermediate, regenerating the Ir photocatalyst and producing a Ni(I) species that can furtherly evolve through an oxidative addition by reaction with an aryl bromide. The final product is generated by a reductive elimination process. Combination of emission and cyclic voltammetry measurements performed for [Ir(dF(CF_3_)ppy)_2_(bpy)]PF_6_ indicates that this species has a reduction potential at an excited state of +1.32 V versus SCE (see Supporting Information, Figure S1). This potential demonstrates that oxidation of the radical precursors employed in this work (*E*_ox_ from −0.87 to −1.11 V vs SCE)^7,41−43,45^ is plausible. Furthermore, comparison of measured reduction potential of the [NiCl_2_(phen)] complex with the data obtained using [Ir(dF(CF_3_)ppy)_2_(bpy)]PF_6_ indicates that Ir(II) + Ni(II) → Ir(III) + Ni(I) is thermodynamically feasible (see Supporting Information, Figure S1). Therefore, our data are fully consistent with the mechanistic scenario previously proposed for Ir/Ni dual catalytic systems.

## 3 Conclusions

The predesign, synthesis, and characterization of a new phenanthroline-containing imine-based layered COF have been performed in this work. This new material was postfunctionalized with Ir and Ni metal complexes in order to obtain a heterogeneous photoredox nickel dual catalytic system. In this unprecedented COF, the phenanthroline group plays a key role in stabilizing the coordination of the metal centers. The obtained **Ir,Ni@Phen-COF** presents high catalytic activity toward photocatalytic cross-couplings between aryl bromides and four different radical precursors (*N*-protected proline, organic alkyl silicates, and potassium benzyl- and alkoxy-trifluoroborates). The comparison between our heterogeneous system and its homogeneous analogues and those reported in the literature makes clear the superiority of the newly obtained catalytic material in terms of activity and stability for a variety of substrates. The different nature of the catalytic systems essayed brings out the good versatility of the new material synthesized. In addition, the sturdiness and the stability of the COF was studied by discarding leaching processes. Recyclability experiments showed that approximatively 1000 TONs for the Ir and more than 400 TONs for the Ni can be reached.

## Supporting Information Available

Materials and methods information; characterization of the materials and compounds; and additional experimental data (PDF)
