# Supplementary material for: Photoredox Heterobimetallic Dual Catalysis Using Engineered Covalent Organic Frameworks
Source: ACS Catal. 2021 Sep 21;11(19):12344–54. doi: 10.1021/acscatal.1c03634 (PMC8650013; doi:10.1021/acscatal.1c03634)
Supplement: Supplementary file 1 — cs1c03634_si_001.pdf [file cs1c03634_si_001.pdf]

SUPPORTING INFORMATION

Photoredox Hetero-Bimetallic Dual Catalysis Using Engineered Covalent Organic Frameworks

Alberto López-Magano,[a] Borja Ortín-Rubio,[b] Inhar Imaz,[b] Daniel Maspoch,[b,c] José Alemán[d,e] and Rubén Mas-Ballesté[a,e*]*

a. Inorganic Chemistry Department, Módulo 7, Universidad Autónoma de Madrid, 28049 Madrid, Spain.

b. Catalan Institute of Nanoscience and Nanotechnology (ICN2), CSIC and The Barcelona Institute of Science and Technology, 08193 Barcelona, Spain.

c. Institució Catalana de Recerca y Estudis Avançats (ICREA), 08010 Barcelona, Spain.

d. Institute for Advanced Research in Chemical Sciences (IAdChem), Universidad Autónoma de Madrid, 28049 Madrid, Spain.

e. Organic Chemistry Department, Módulo 1, Universidad Autónoma de Madrid, 28049 Madrid, Spain.

Corresponding Author

Rubén Mas-Ballesté E-mail: ruben.mas@uam.es

José Alemán E-mail: jose.aleman@uam.es

Table of contents

Materials and general methods. 2

Synthesis of building blocks and metal complexes. 4

Synthesis and characterization of COFs 7

General procedure for the light-mediated cross-coupling between potassium benzyltrifluoroborate and aryl bromides 28

Leaching experiments 32

Procedure for the light-mediated cross-coupling between potassium *tert*-butoxymethyltrifluoroborate and aryl bromides. 33

Procedure for the light-mediated cross-coupling between organic silicates and aryl bromides. 33

Procedure for the light-mediated decarboxylative arylation of α-aminoacids with aryl bromides. 35

Benchmarking of catalytic results. 36

PXRD Simulation 37

References 57

Materials and general methods.

All reagents and solvents were purchased from commercial sources and used without further purification.

Light irradiation was carried out using a 15W blue LED photoreactor thermostated at 25 ˚C. A spectro-radiometer equipment Stellarnet model Blue-Wave UV-NB50 was employed to measure the emission of the Blue LED used (range 300-600 ʎ, integration time CR2-AP + 200 ms, intensity 21.7217 W/m^2).

Nuclear Magnetic Resonance (NMR) spectra were acquired on a *Bruker AV-300 spectrometer*, running at 300 MHz for ^1H. Chemical shifts (δ) are reported in ppm relative to residual solvent signals ($CDCl_3$: 7.26 ppm for ^1H-NMR. ^{13}C solid State Nuclear Magnetic Resonance were acquired on a *Bruker AV-400 spectrometer* coupled to a multinuclear probe (^{15}N-^{31}P) CPMAS with triple channel (BL4 X/Y/^1H) for a 4 mm rotor at room temperature, using 1k scans and 12 kHz of turning speed. The ^1H excitation pulse used is $\pi/2$ * 2.75 μs and the contact pulse is 3 ms.

Elemental Chemical Analyses were obtained by *Servicio Interdepartamental de Apoyo a la Investigación* in UAM, in an elemental analyzer *LECO CHNS-932* model number 601-800-500.

IR spectra were recorded in a *Perkin-Elmer 283* equipped with *ATR MIRacle Single Reflection Horizontal*.

Powder X-ray difraction was obtained in a *X'Pert PRO* difractometer θ/2θ geometry from *Panalytica*l equipped with a *Johansson* monochromator for ʎ K_α, a *X'Celerator* fast detector in an alumina holder. The θ/2θ swept was performed from 1 to 45 ˚ with an angular increase of 0.0167 ˚/100 s. Simulated model structures of PXRD were carried out using Materials Studio 8.0 Program. Manual editing from a similar reported structure was performed and adjusted by using the forcite module.

Scanning Electron Microscopy (SEM) images were carried out on a *Hitachi S-3000N electron microscope* with a coupled ESED detector and an analyzer from energy dispersive X-ray from *Oxford Instruments*, *INCAx-sight* model. The images were obtained in vacuum after being metallized in a *Sputter Quórum Q150T-S* with gold coating. **Transmission Electron Microscopy** (TEM) images were collected on a Tecnai G2 F20 microscope (FEI) at 200 KV.

Diffuse reflectance was measured in a UV/Vis spectrometer by UNICAM. Emission intensities were recorded using a JASCO Spectrofluorometer FP-8600 controlled by Spectra Manager Version 2.10.01. The **fluorescence emission** of the materials was measured irradiating a suspension of 1 mg of the sample dispersed in 2 mL of MeOH at 390 nm. A 10x10 mm precision cell made of quartz was used for all emission measurements.

Electrochemistry: Voltammetry experiments were performed under argon atmosphere at room temperature, using 0.1 M tetrabutylammonium hexafluorophosphate solution in CH_3CN as electrolyte. Measurements were carried out by using an Ivium CompaqStat potentiostat interfaced with a computer. A standard three-electrode electrochemical cell was used. Potentials were referred to an Ag/AgCl, Et_4NBr 0.4 M reference electrode in ethylene glycol, and measured potentials were calibrated using an internal Fc/Fc+ standard. The working electrode consists of a Pt electrode coated with Teflon. A carbon paste was fabricated by mixing 30 mg of high purity carbon (>99.9%) with 3 mg of the material and 1 drop of mineral oil (Nujol). This carbon paste was deposited in the surface of the working electrode to perform the experiments.

As control experiment, a blank carbon paste containing only high purity carbon and Nujol was obtained and measured under the same conditions.

Thermogravimetric analyses (TGA) were performed on a TGA Q500 Thermobalance from TA instruments, heating the sample from 25 ^{o}C to 900 ^{o}C at 10 ^{o}C/min under air atmosphere.

X Ray Photoelectron Spectroscopy (XPS) were performed with a Phoibos 150 analyzer (SPECS GmbH) in ultra-high vacuum conditions (base pressure 4×10^{-10} mbar) with a monochromatic aluminum K_α x-ray source (1486.74 eV) at 300 W. The energy resolution as measured by the FWHM of the Ag $3d_{5/2}$ peak for a sputtered silver foil was 0.62 eV. The binding energy was corrected by using the C 1s line at 284.5 eV as the reference. A flood gun was used during acquisition to compensate the surface charging of the samples.

Qualitative and quantitative **Total X-Ray Fluorescence** analyses (TXRF) were performed with a benchtop *S2 PicoFox* TXRF spectrometer from Bruker Nano (Germany) [1,2]. TXRF system was equipped with a Mo X-ray source working at 50 kV and 600 µA, a multilayer monochromator with 80% of reflectivity at 17.5 keV (Mo K_α), a XFlash SDD detector with an effective area of 30 mm^2 and an energy resolution better than 150 eV for 5.9 keV (Mn K_α). For deconvolution and integration commercial Spectra v. 7.5.3 software package from Bruker was used. Previously to the measurements, sample acid digestions were performed in a high pressure and temperature microwave. Acid digestion technology was used by mean of an UltraWAVE digestion system from Millestone (Italy) with a single reaction chamber able of operates up to 199 bar pressure and 270 °C.

Volumetric N_2 sorption isotherms were collected at 77 K (N_2) using an ASAP 2460 HD (Micromeritics). Temperature was controlled by using a liquid nitrogen bath.

Synthesis of building blocks and metal complexes.

Synthesis of 1: 3,8-dibromo-1,10-phenanthroline (1 g, 2.96 mmol), (4-formylphenyl)boronic acid (930 mg, 6.20 mmol) and K_2CO_3 (1.625 g, 8.87 mmol) were introduced into a 100 mL Schlenk equipped with a stir bar. Three vacuum-Ar cycles were performed. Then, 20 mL of purged THF and 4 mL of distilled water were added under Ar atmosphere. Finally, $Pd(PPh_3)_4$ (512 mg, 0.443 mmol) was added under Ar atmosphere. The reaction was heated at 70 ^{0}C during 48 hours in presence of Ar. A white solid was precipitated. Then, the reaction was cool down and filtered. The white solid was washed with water and acetone. Finally, it was recrystallized in a mixture of hot $CHCl_3$-acetone, in order to afford white crystals of **1**, that were isolated by filtration and dried under vacuum. 1118 mg obtained (97 % of yield).

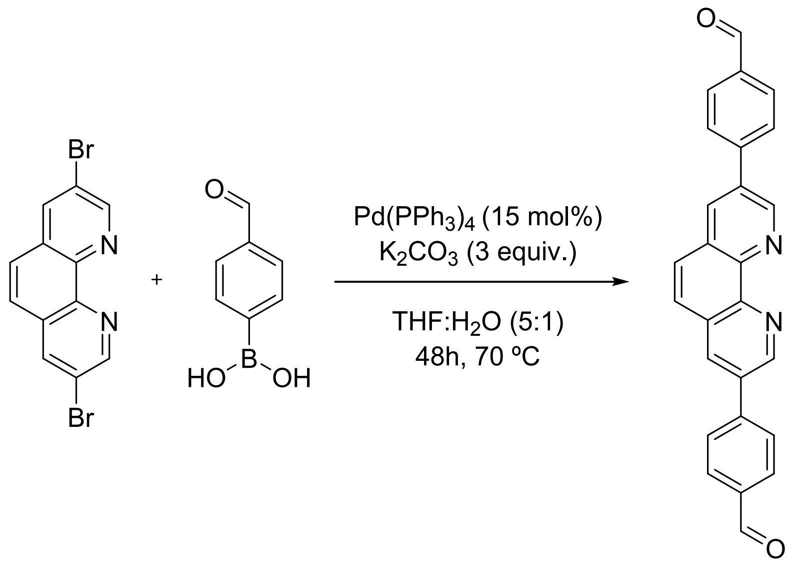

1

^{1}H-NMR (300 MHz, $CDCl_3$) δ ^{1}H NMR (300 MHz, $CDCl_3$) δ 10.14 (s, 2H), 9.50 (d, *J* = 2.3 Hz, 2H), 8.51 (d, *J* = 2.3 Hz, 2H), 8.09 (d, *J* = 8.3 Hz, 4H), 7.98 (d, *J* = 8.3 Hz, 4H), 7.96 (s, 2H) ppm.

Elemental analysis: calculated for $C_{26}H_{16}N_2O_2$: C: 80.40%; H: 4.15%; N: 7.21%. Found: C: 80.53%; H: 4.45%; N: 7.11%.

Synthesis of the Ir precursor.

Step 1 – Synthesis of 2-(2,4-difluorophenyl)-5-(trifluoromethyl)pyridine (L1)[3]

Potassium (2,4-difluorophenyl)trifluoroborate (1.1 g, 5 mmol), 2-bromo-5-(trifluoromethyl)pyridine (753 mg, 3.33 mmol), K_2CO_3 (2.3 g, 16.7 mmol) and $Pd(PPh_3)_4$ (390 mg, 0.33 mmol) were placed into a 100 mL Schlenk equipped with a stir bar. Three vacuum-Ar cycles were performed. Then, 12 mL of degassed THF and 6 mL of degassed distilled water were added. The reaction was stirred at 80 ^{o}C under Ar atmosphere for 24 hours. After cooling to room temperature, it was extracted with 3x20 mL of dichloromethane. The organic layers were combined and dried with Na_2SO_4, filtrated and evaporated under reduced pressure. After purification by silica gel chromatography (cyclohexane-AcOEt 20:1), **L1** was isolated as a white solid (847 mg, 97 %).

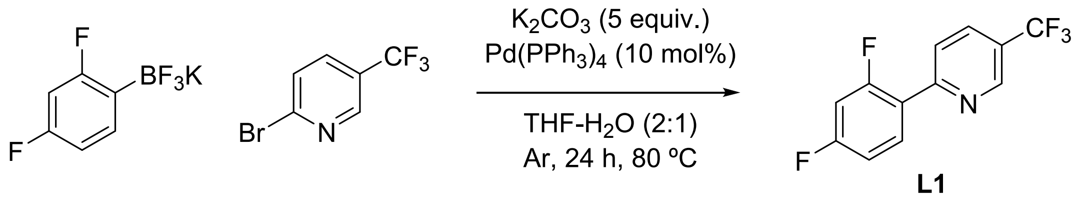

^{1}H NMR (300 MHz, $CDCl_3$): δ = 8.96 (s, 1H), 8.12-8.07 (m, 1H), 7.98 (dd, *J* = 8.5, 2.0 Hz, 1H), 7.90 (d, *J* = 8.5 Hz, 1H), 7.06-7.02 (m, 1H), 6.97-6.92 (m, 1H).

Step 2 – Synthesis of dimer [(dF(CF$_3$)ppy)$_2$-Ir-μ-Cl]$_2$ [4]

L1 (840 mg, 3.24 mmol) and $IrCl_3$ (440 mg, 1.47 mmol) were placed into a 100 mL Schlenk equipped with a stir bar. Three vacuum-Ar cycles were performed. Then, 18 mL of degassed 2-ethoxyethanol and 6 mL of degassed distilled water were added. The reaction was stirred at 120 ºC under Ar atmosphere for 24 hours. After cooling to room temperature, the yellow solid formed was filtrated and gently washed with deionized water and cyclohexane, and finally dried under vacuum. 1.639 g obtained, 75 % yield.

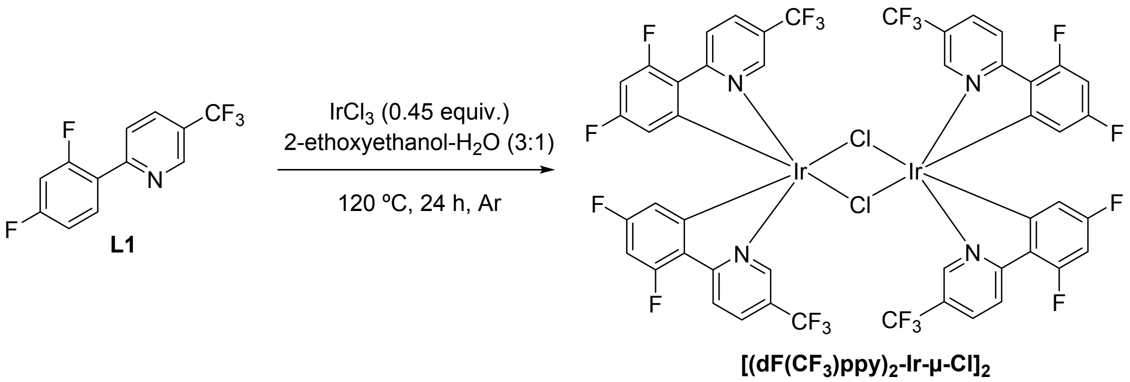

^{1}H NMR (300 MHz, $CDCl_3$): 9.50 (d, *J* = 1.0 Hz, 4H), 8.45 (dd, *J* = 9.0, 3.0 Hz, 4H), 8.04 (dd, *J* = 9.0, 2.0 Hz, 4H), 6.39-6.47 (m, 4H), 5.06 (dd, *J* = 9.0, 2.0 Hz) ppm.

Synthesis of [NiCl$_2$(phen)] complex [5]

A 50 mL Schlenk flask was charged with a stir bar, $NiCl_2$ (77.7 mg, 0.6 mmol), and 1,10-phenanthroline (108,1 mg, 0.6 mmol). Three vacuum/Ar cycles were performed. Then, 5 mL of degassed EtOH were added. The pink reaction mixture was stirred at room temperature overnight. A green solid appeared, indicating the completion of the reaction. The solvent was evaporated under reduced pressure, and the crude was suspended in 10 mL of THF. The green solid was isolated by filtration and dried overnight under vacuum.

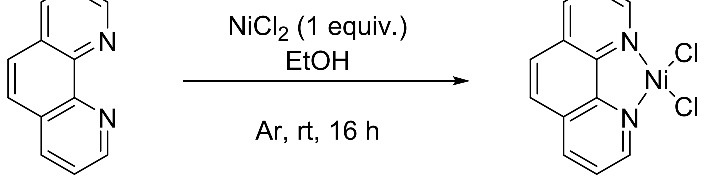

Elemental Analysis: calculated for $C_{12}H_8Cl_2N_2Ni$: C, 46.52; H, 2.60; N, 9.04. Found: C, 46.89; H, 2.61; N, 9.04.

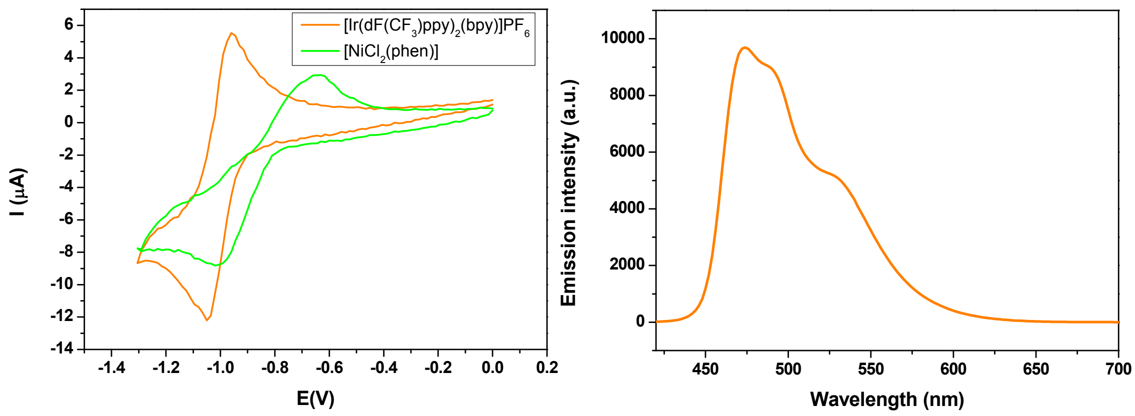

Figure S1: Left, Cyclic voltammetry of [$NiCl_2$(phen)] (black line) compared with [Ir(dF(CF_3)ppy)$_2$(bpy)]PF_6 (red line) measured in DMF (0.1 M $TBAPF_6$) at 100 mV/s using glassy carbon electrode as WE, Ag/AgCl as RE and Pt bar as CE. Right, emission spectra of [Ir(dF(CF_3)ppy)$_2$(bpy)]PF_6.

Synthesis and characterization of COFs

Table S1: Screening of reaction conditions for the synthesis of Phen-COF.

Entry	Solvent	Catalyst	T (ºC)	Time (h)	Result
1	Dioxane	AcOH 6M	120	72	Amorphous
2	Mesitylene	AcOH 6M	145	72	Amorphous
3	NMP	AcOH 6M	120	72	No material
4	o-DCB	AcOH 6M	120	72	No material
5	TCB, tBuOH (1:1)	AcOH 6M	80	72	Amorphous
6	NMP, o-DCB (1:1)	AcOH 3M	100	96	Amorphous
7	DMA	AcOH 9M	140	72	Amorphous
8	ACN, DMF (1:1)	AcOH 6M	100	72	Amorphous
9	DMA, DMSO (1:1)	AcOH 9M	150	96	Amorphous
10	Dioxane, Mesitylene (1:1)	AcOH 6M	120	72	Amorphous, one diffraction peak with low intensity
11	Dioxane, Mesitylene (1:2)	AcOH 6M	120	72	Amorphous, one diffraction peak with low intensity
12	Dioxane, Mesitylene (2:1)	AcOH 6M	120	72	Amorphous, one diffraction peak with low intensity
13	Dioxane, Mesitylene (1:4)	AcOH 6M	120	72	Amorphous, one diffraction peak with low intensity
14	Dioxane, Mesitylene (4:1)	AcOH 6M	120	72	Amorphous
15	Dioxane, Mesitylene (1:9)	AcOH 6M	120	72	Moderated Crystallinity
16	Dioxane, Mesitylene (9:1)	AcOH 6M	120	72	Amorphous
17	Dioxane, Mesitylene (1:9)	AcOH 3M	120	72	Amorphous
18	Dioxane, Mesitylene (1:9)	AcOH 9M	120	72	Amorphous
19	Dioxane, Mesitylene (1:9)	CF_3SO_3H	120	72	Black Powder
20	Dioxane, Mesitylene (1:9)	$Sc(OTf)_3$	120	72	Amorphous
21	Dioxane, Mesitylene (1:9)	AcOH 6M	120	144	Moderated Crystallinity

Synthesis of Phen-COF: A 45 mL solvothermal reactor was charged with **1** (100 mg, 0.257 mmol) and **TAPB** (60 mg, 0.170 mmol). 5.4 mL of mesitylene and 0.6 mL of anhydrous dioxane were added. Finally, 0.6 mL of aqueous acetic acid 6 M were added. The reactor was sealed and heated at 120 ˚C for 3 days, yielding a yellow-brown solid which was isolated by filtration and washed with DMF, MeOH and THF. The resulting powder was immersed in anhydrous THF for 24 h, filtered and dried first at room temperature under vacuum for 12 h, and then at 100 ˚C for 2 h to afford a yellow-brown powder, **Phen-COF**. 140 mg obtained.

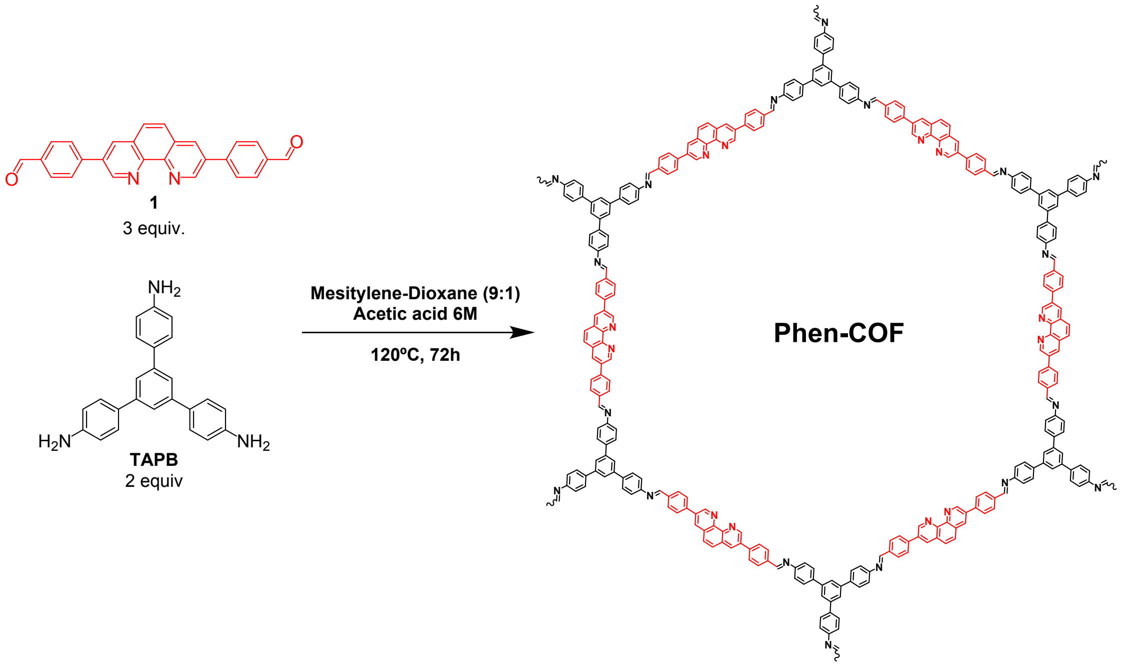

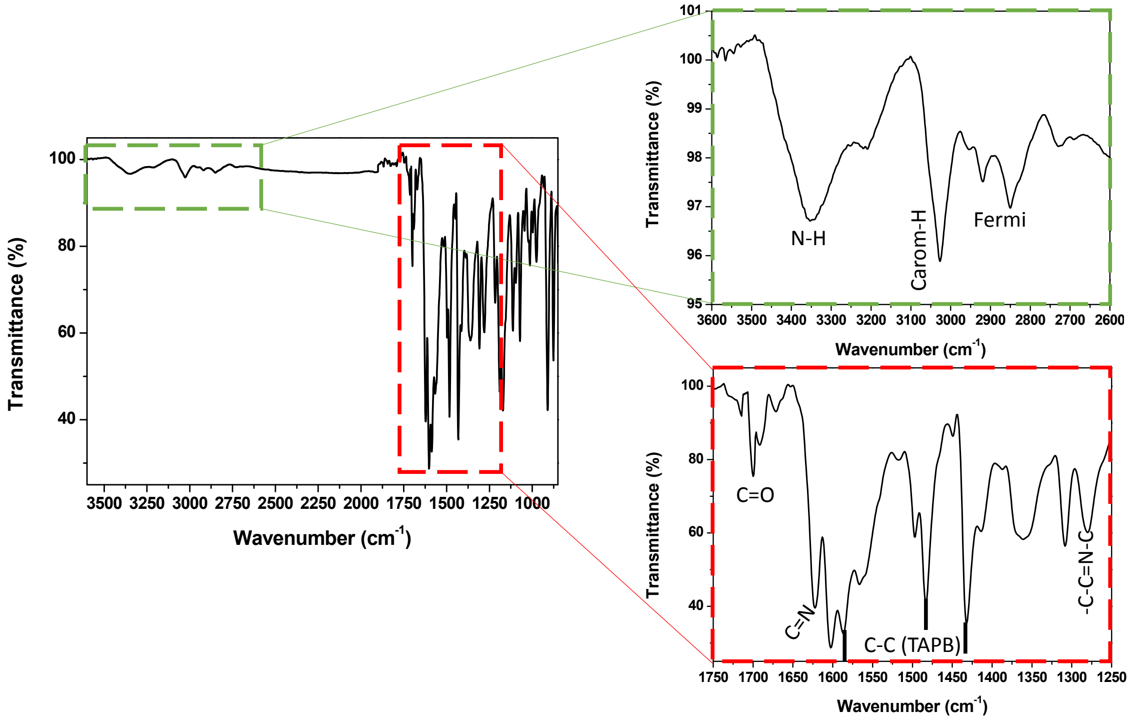

Figure S2: Fourier-Transformed Infrared Spectra of Phen-COF.

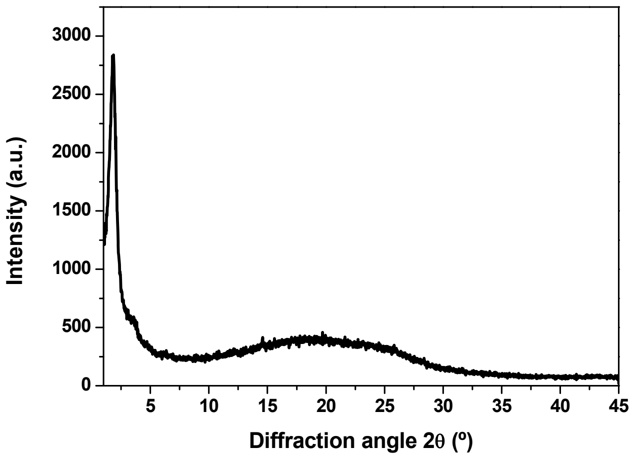

Figure S3: PXRD pattern of Phen-COF.

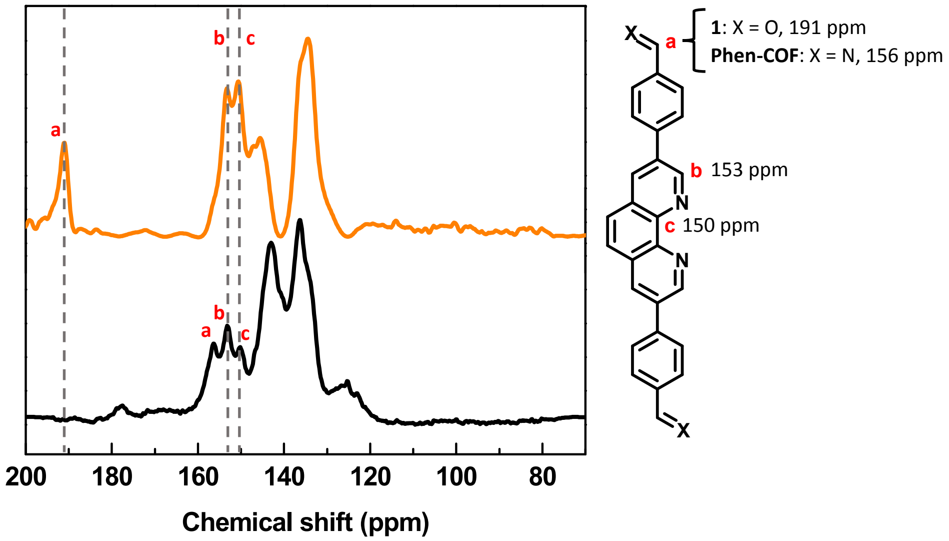

Figure S4: ^{13}C-NMR-CP-MAS spectra of 1 (orange line) and Phen-COF (black line)(for the assignment of a, b and c signals, see the drawing of the molecule).

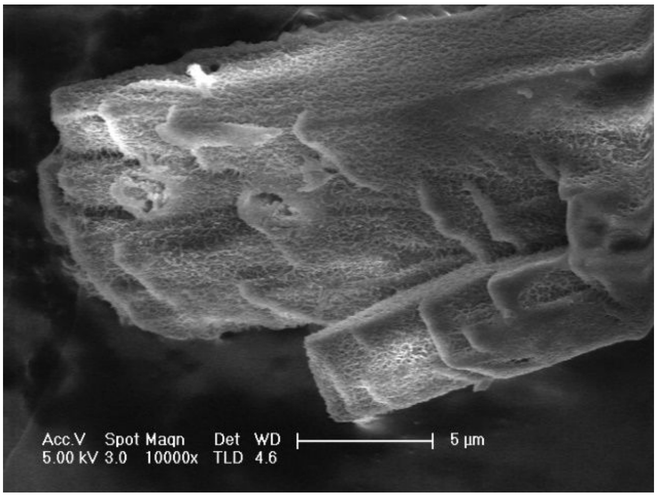

Figure S5: FE-SEM image of Phen-COF.

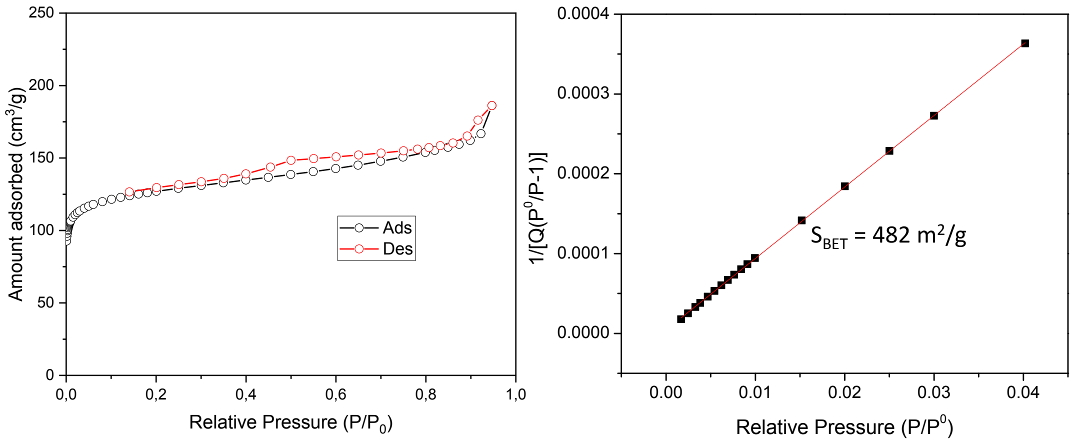

Figure S6: Adsorption-desorption N_2 isotherm at 77 K of Phen-COF and Brunauer-Emmett-Teller surface area plot of Phen-COF (S_{BET} = 482 m^2/g).

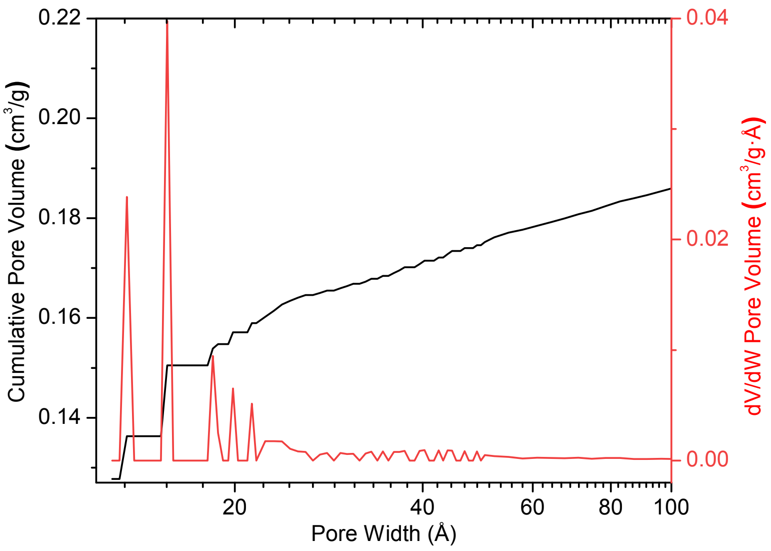

Figure S7: Pore size distribution of Phen-COF.

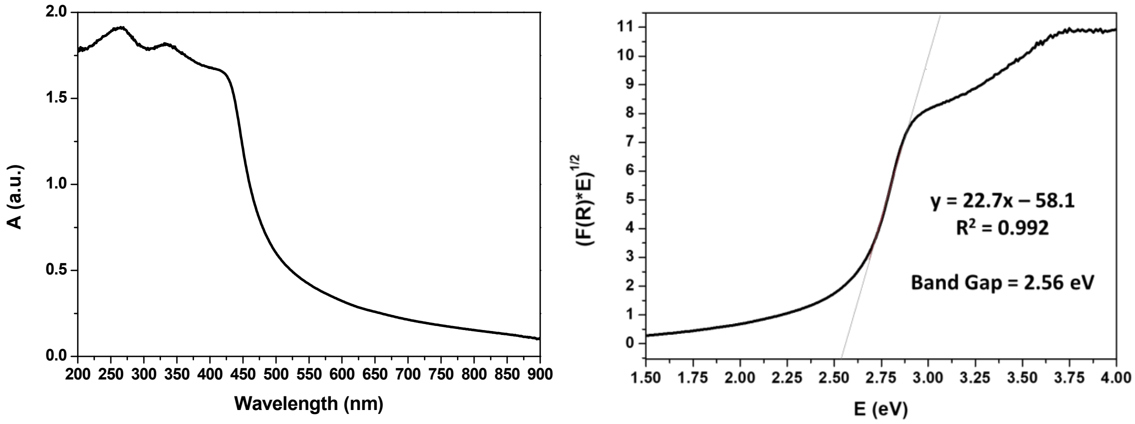

Figure S8: Diffuse Reflectance Spectrum and Kubelka-Munk plot of Phen-COF.

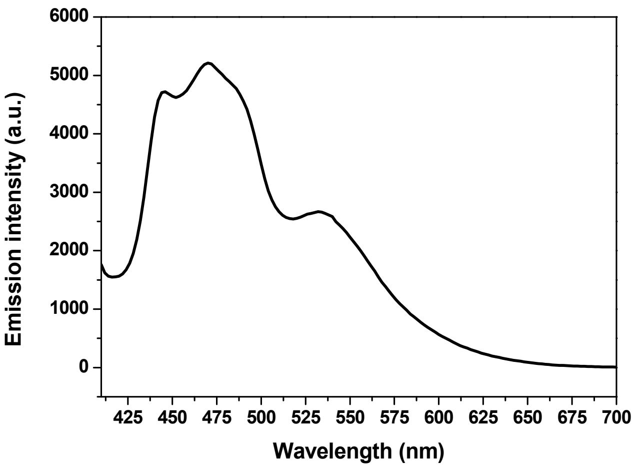

Figure S9: Emission Spectrum of Phen-COF.

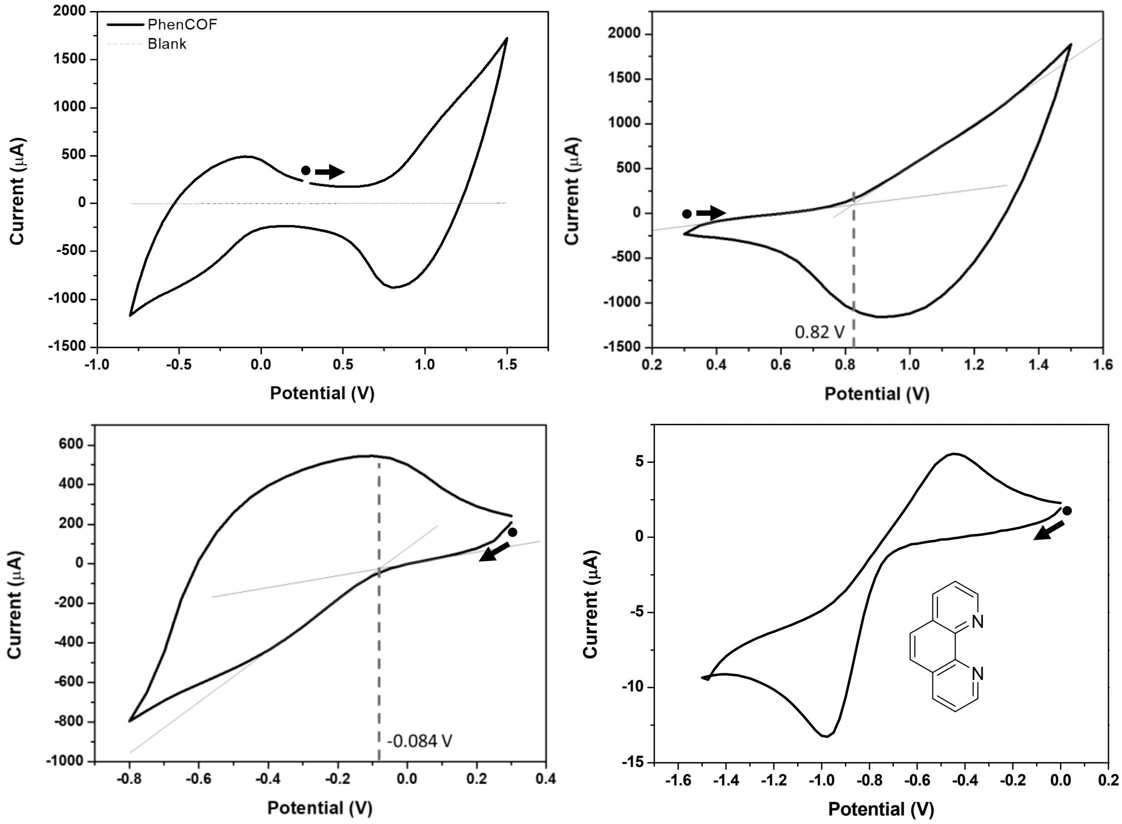

Figure S10: Cyclic voltammetries of Phen-COF and comparison with cyclic voltammetry of 1,10-phenanthroline (bottom, right).

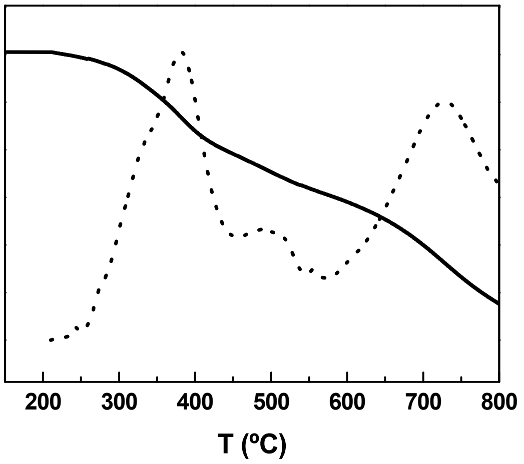

Figure S11: Thermogravimetric analysis of Phen-COF. The dotted line corresponds to the first derivative of Weight %.

Synthesis of Ir,Ni@Phen-COF: in a 100 mL round bottom-flask, 140 mg of **Phen-COF** were suspended on 50 mL of dry acetonitrile and 1 mL of aqueous acetic acid 6 M during 30 minutes with an ultrasound bath. Then, $[(dF(CF_3)ppy)_2\text{-Ir-}\mu\text{-Cl}]_2$ (33 mg, 0.022 mmol) and $NiCl_2$·glyme (19.6 mg, 0.089 mmol) were added. The mixture was stirred at 70 ºC for 16 hours, yielding a yellow powder that was isolated by filtration and washed with hot DMF, acetonitrile and THF. The resulting powder was washed with acetone using Soxhlet extraction, then filtered and dried first at room temperature under vacuum for 12 h, and then at 100 ˚C for 2 h to afford an orange powder, **Ir,Ni@Phen-COF**. 151 mg obtained.

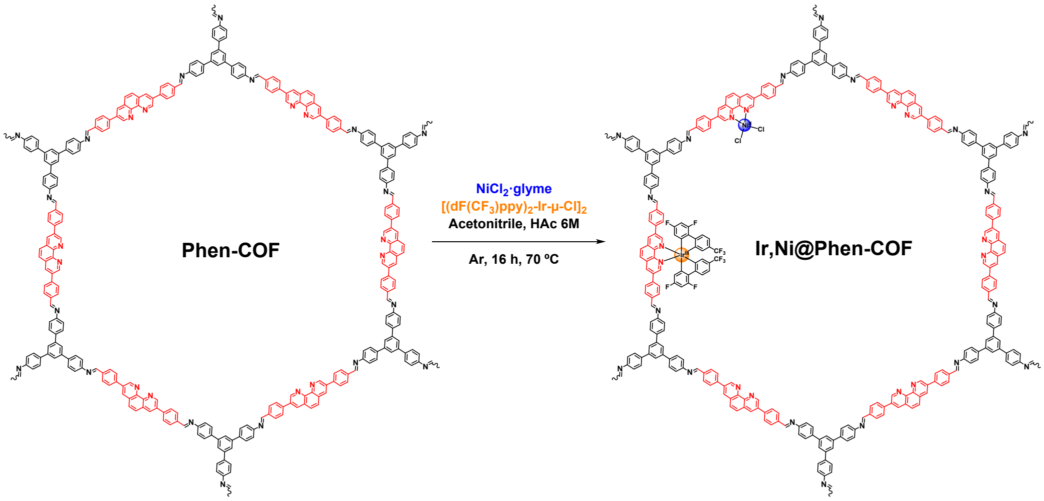

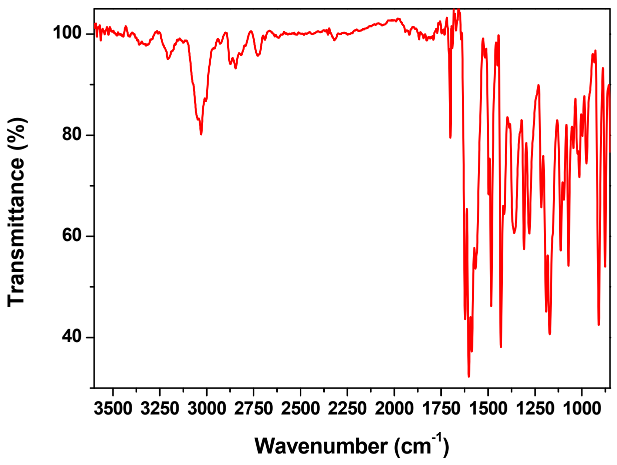

Figure S12: Fourier-Transformed Infrared Spectra of Ir,Ni@Phen-COF.

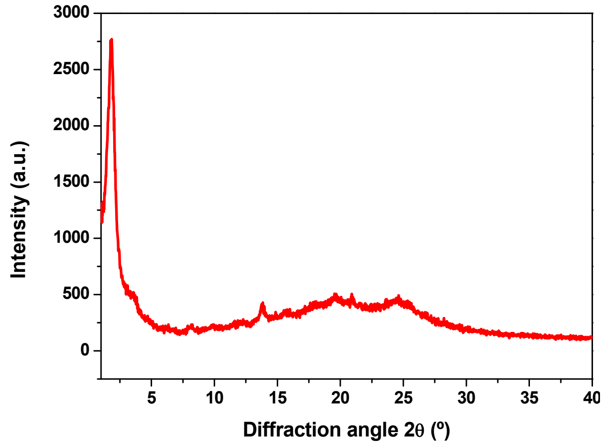

Figure S13: PXRD pattern of Ir,Ni@Phen-COF.

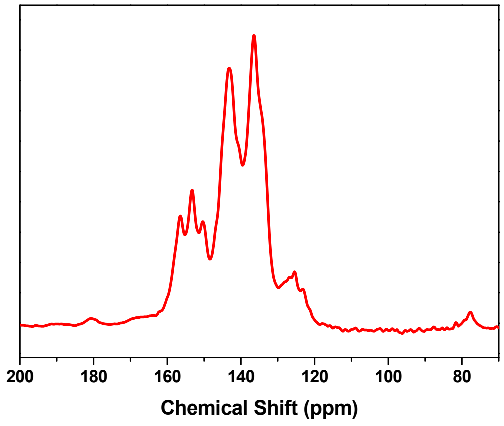

Figure S14: ^{13}C-NMR-CP-MAS spectra of Ir.Ni@Phen-COF.

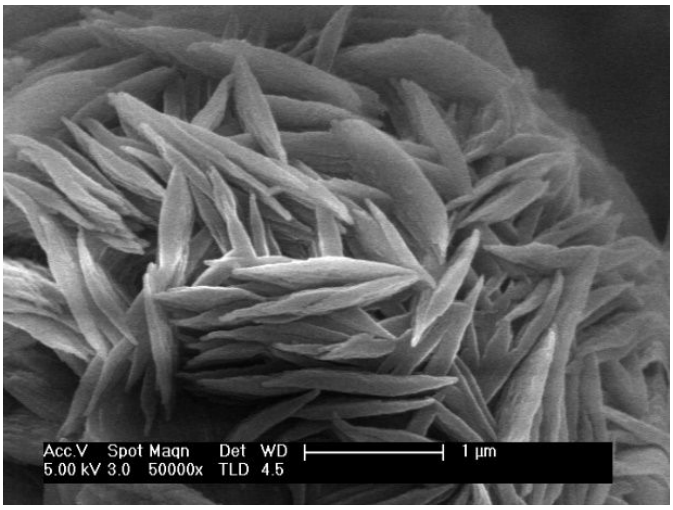

Figure S15: FE-SEM image of Ir,Ni@Phen-COF.

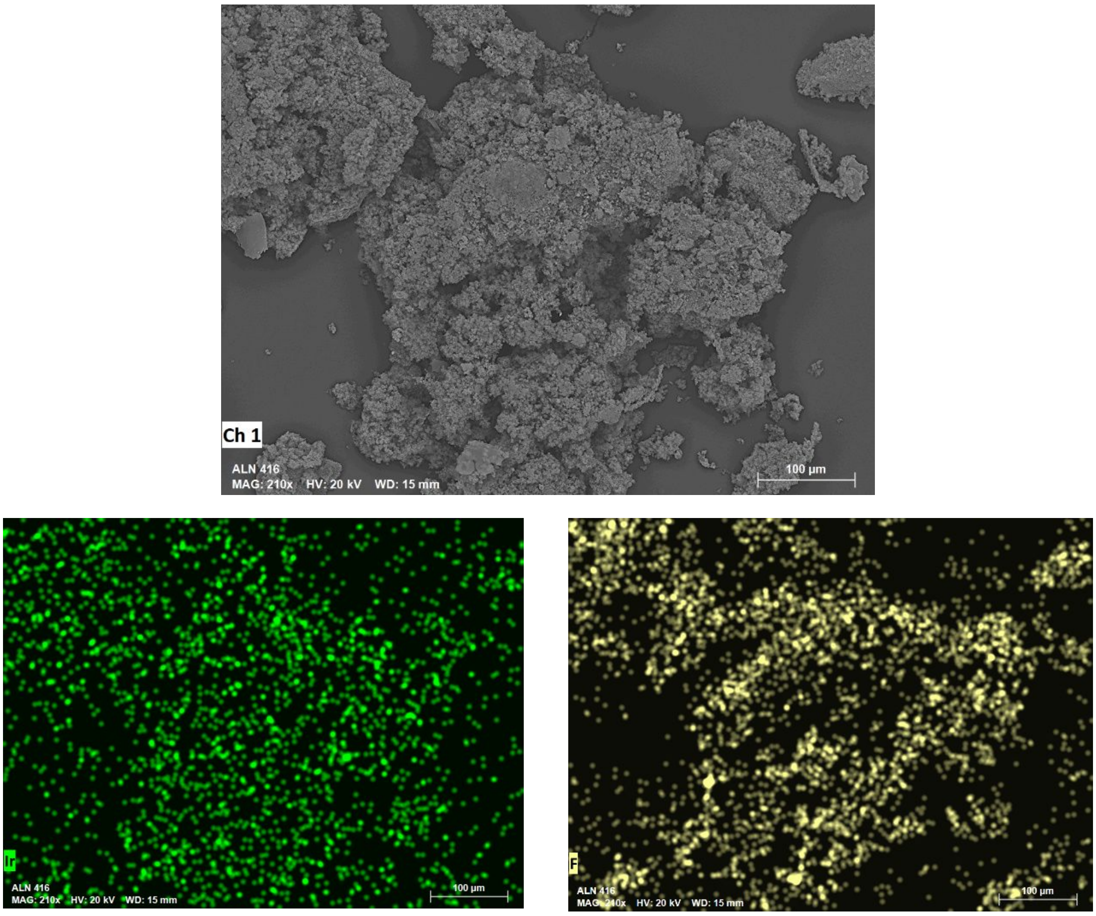

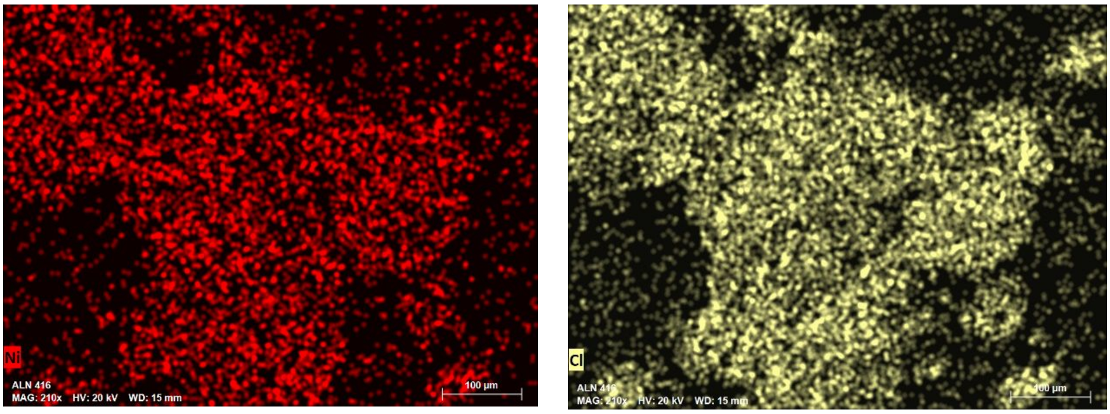

Figure S16: SEM-EDX images of Ir,Ni@Phen-COF (Ir-green, Ni-red, F and Cl-white).

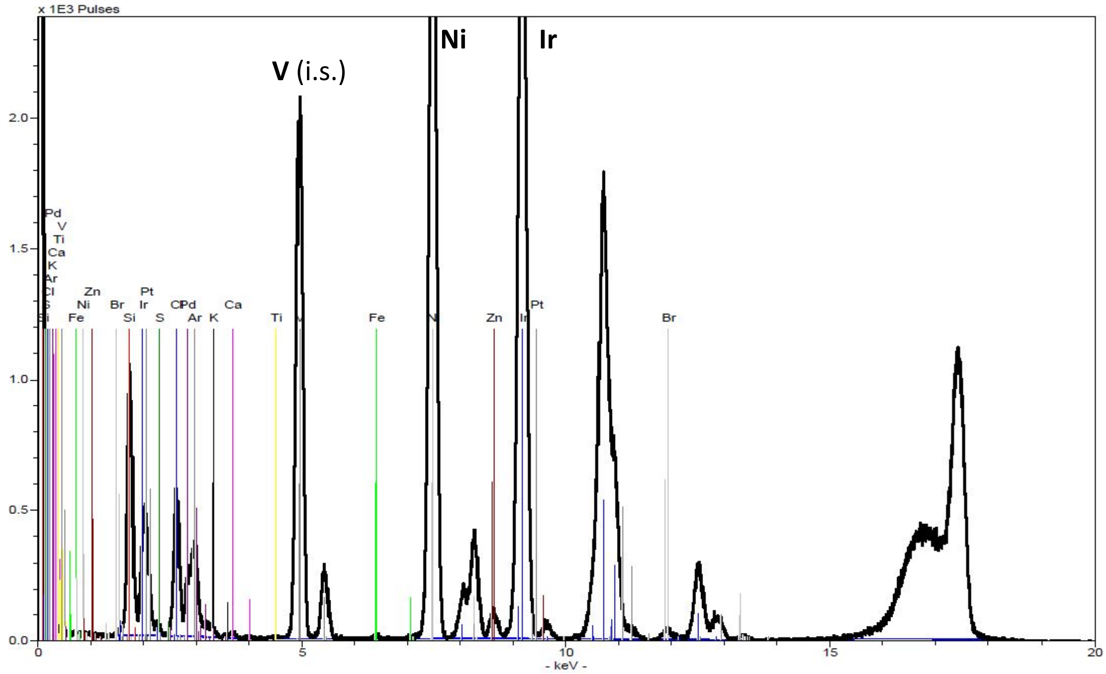

Figure S17: Quantitative TXRF spectra of Ir,Ni@Phen-COF (4.0 % of Ir and 2.8 % of Ni).

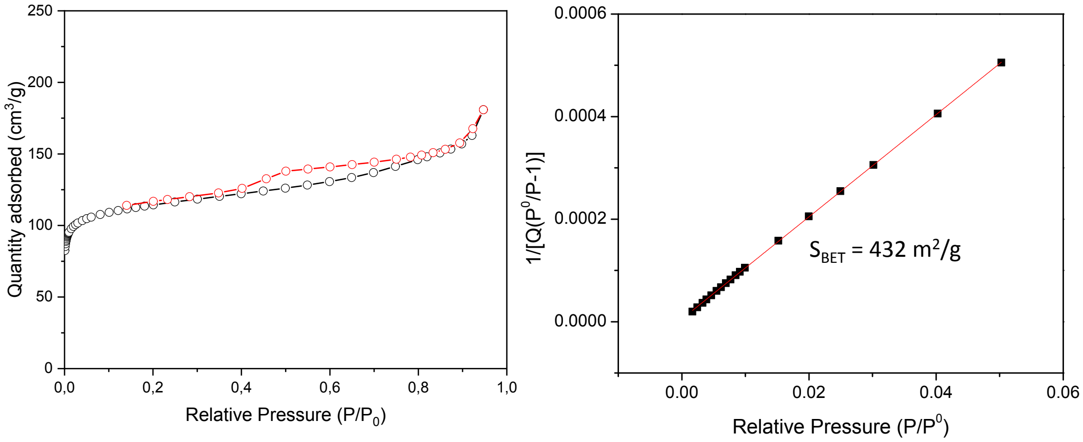

Figure S18: Adsorption-desorption N_2 isotherm at 77 K of Ir,Ni@Phen-COF and Brunauer-Emmett-Teller surface area plot of Ir,Ni@Phen-COF (S_{BET} = 432 m^2/g).

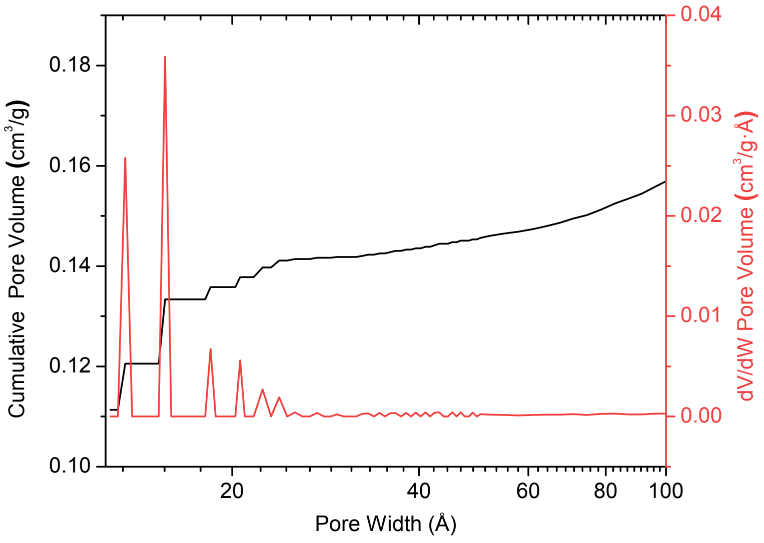

Figure S19: Pore size distribution of Ir,Ni@Phen-COF.

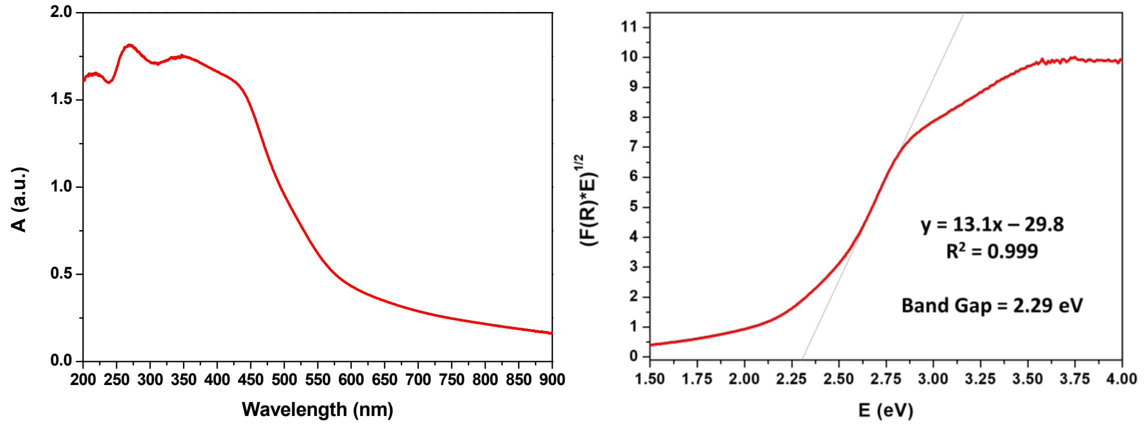

Figure S20: Diffuse Reflectance Spectrum and Kubelka-Munk plot of Ir,Ni@Phen-COF.

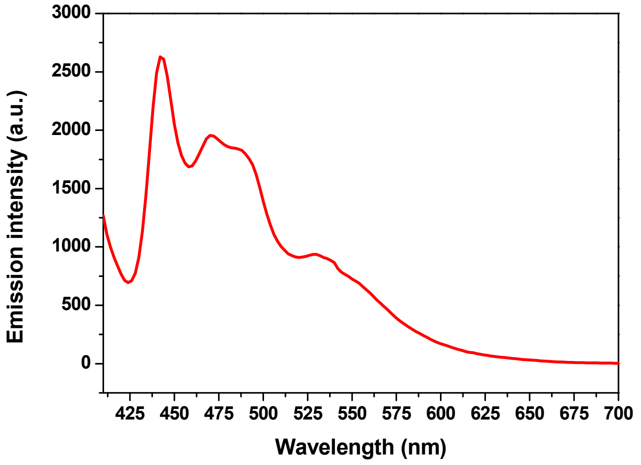

Figure S21: Emission Spectrum of Ir,Ni@Phen-COF.

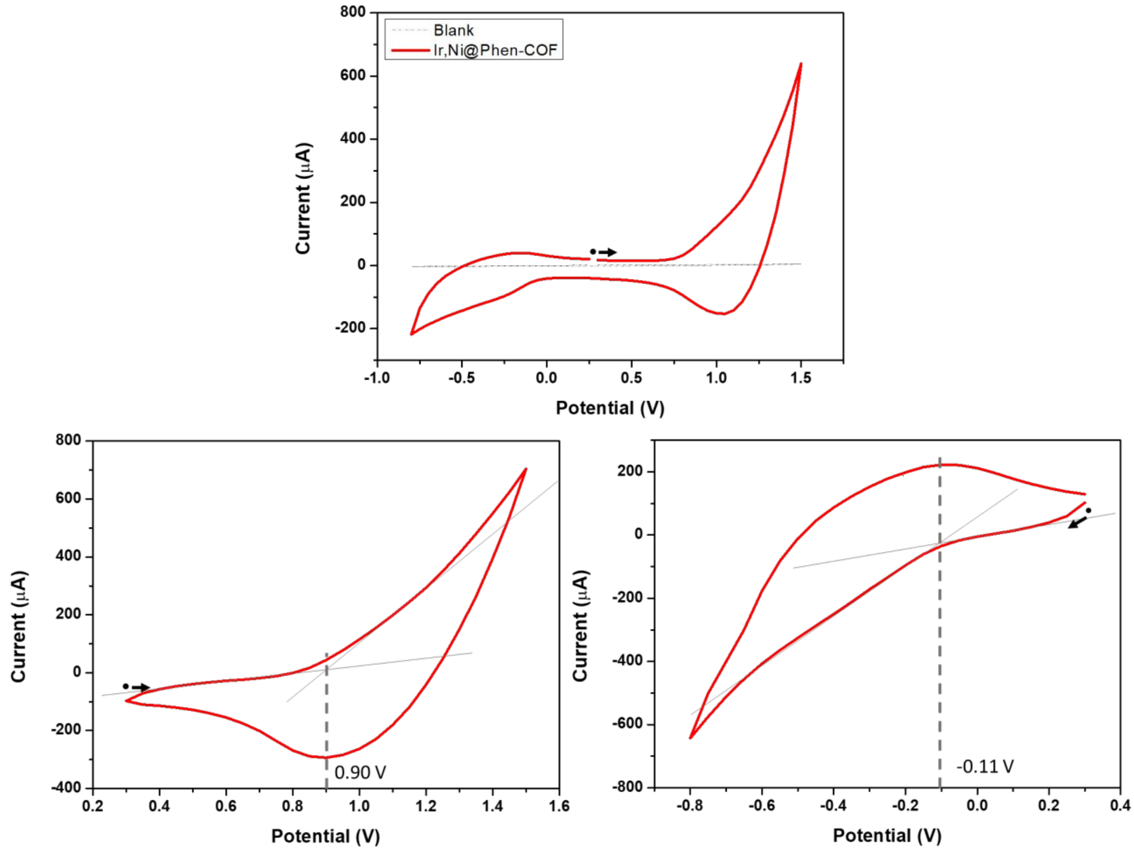

Figure S22: Cyclic voltammetries of Ir,Ni@Phen-COF.

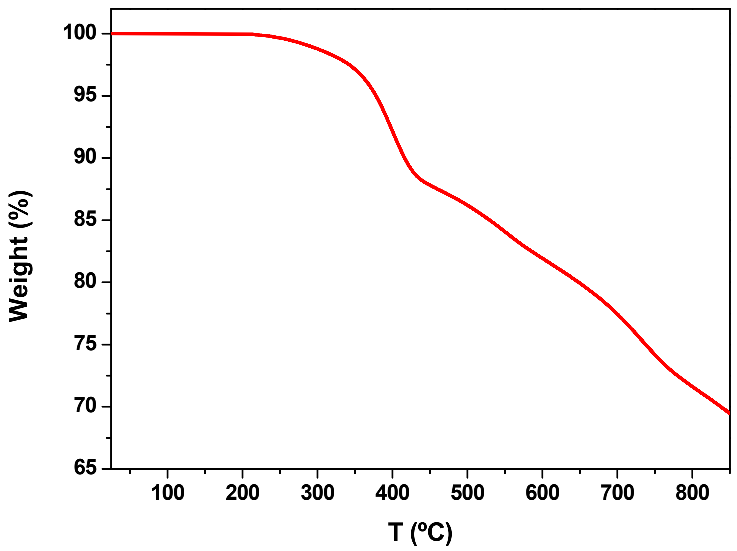

Figure S23: Thermogravimetric analysis of Ir,Ni@Phen-COF.

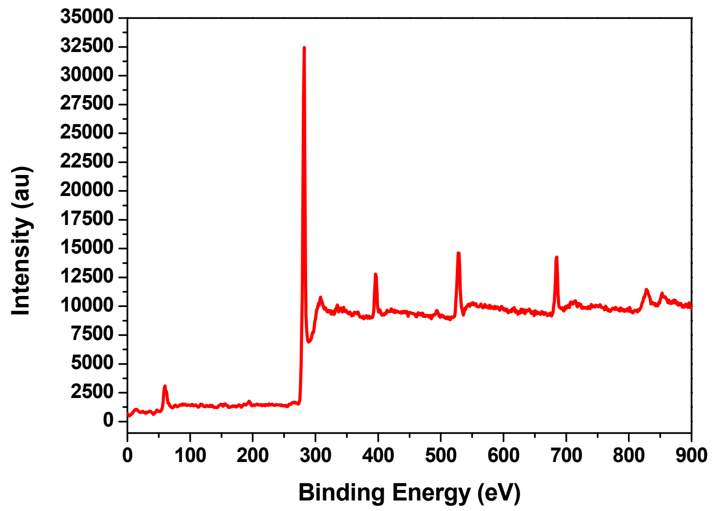

Figure S24: Overview of X-Ray Photoelectron Spectrum of Ir,Ni@Phen-COF.

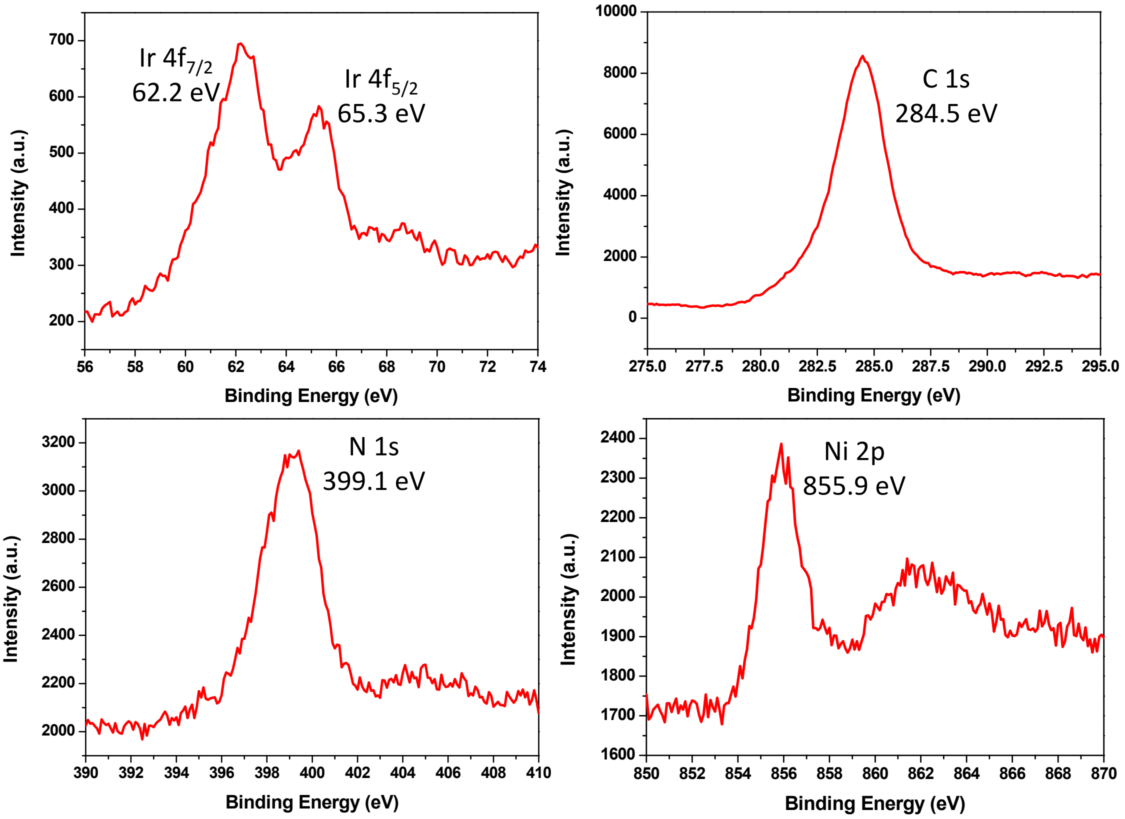

Figure S25: XPS spectra of Ir,Ni@Phen-COF.

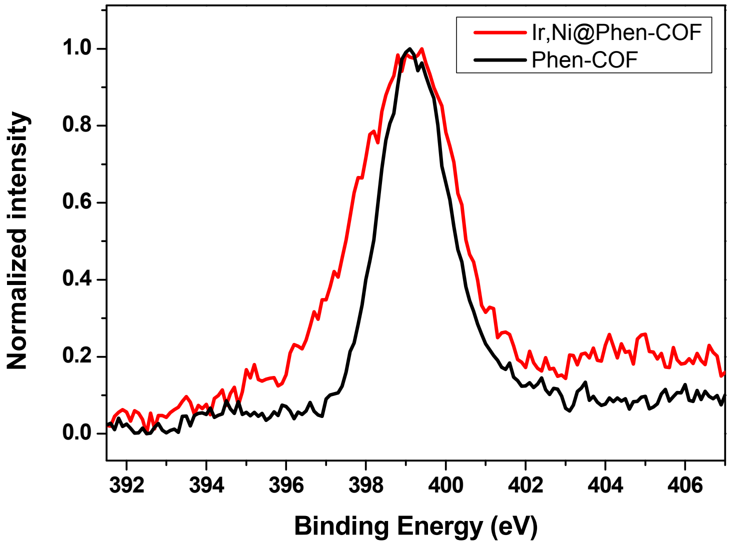

Figure S26: Comparison of N 1s XPS spectra of Phen-COF and Ir,Ni@Phen-COF.

Synthesis of Ni@Phen-COF: in a 100 mL round bottom-flask, 140 mg of **Phen-COF** were suspended on 50 mL of dry acetonitrile and 1 mL of aqueous acetic acid 6 M during 30 minutes with an ultrasound bath. Then, $NiCl_2$·glyme (19.6 mg, 0.089 mmol) was added. The mixture was stirred at 70 ºC for 16 hours, yielding a yellow powder that was isolated by filtration and washed with hot DMF, acetonitrile and THF. The resulting powder was washed with acetone using Soxhlet extraction, then filtered and dried first at room temperature under vacuum for 12 h, and then at 100 ˚C for 2 h to afford an orange powder, **Ni@Phen-COF**. 145 mg obtained.

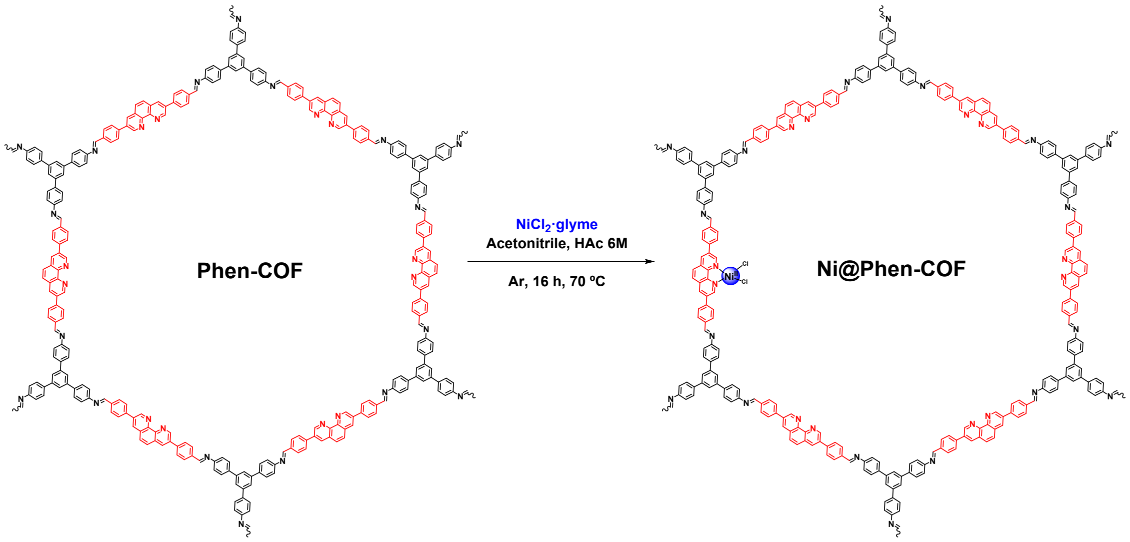

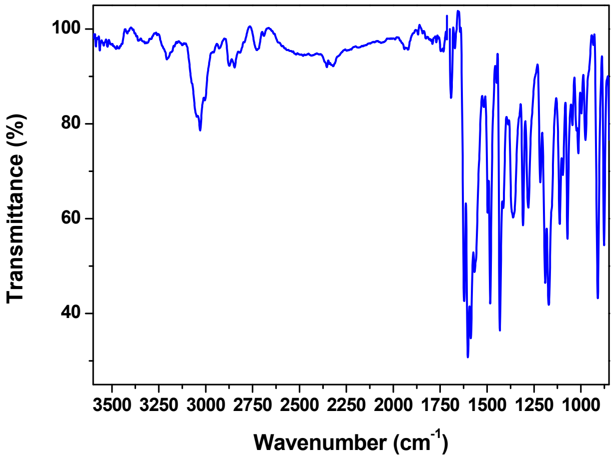

Figure S27: Fourier-Transformed Infrared Spectra of Ni@Phen-COF.

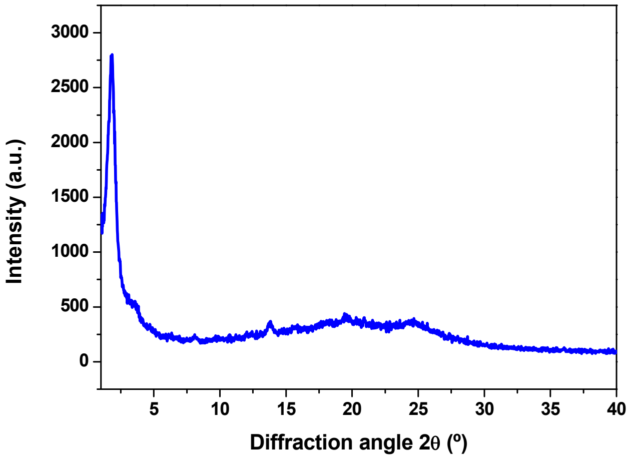

Figure S28: PXRD pattern of Ni@Phen-COF.

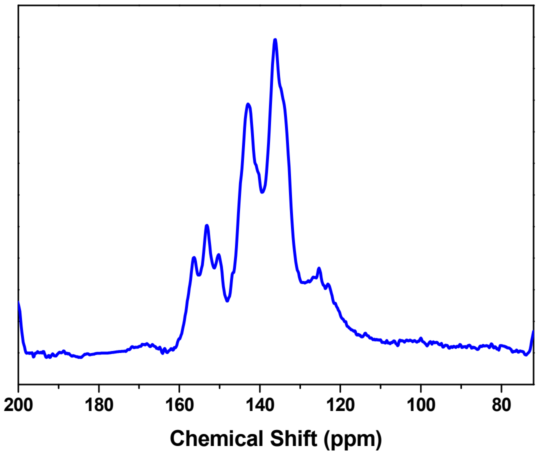

Figure S29: ^{13}C-NMR-CP-MAS spectra of Ni@Phen-COF.

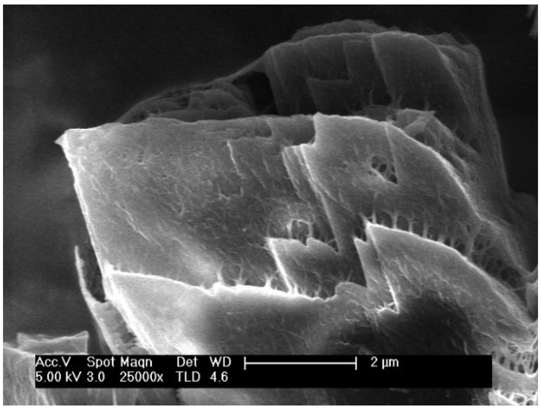

Figure S30: FE-SEM image of Ni@Phen-COF.

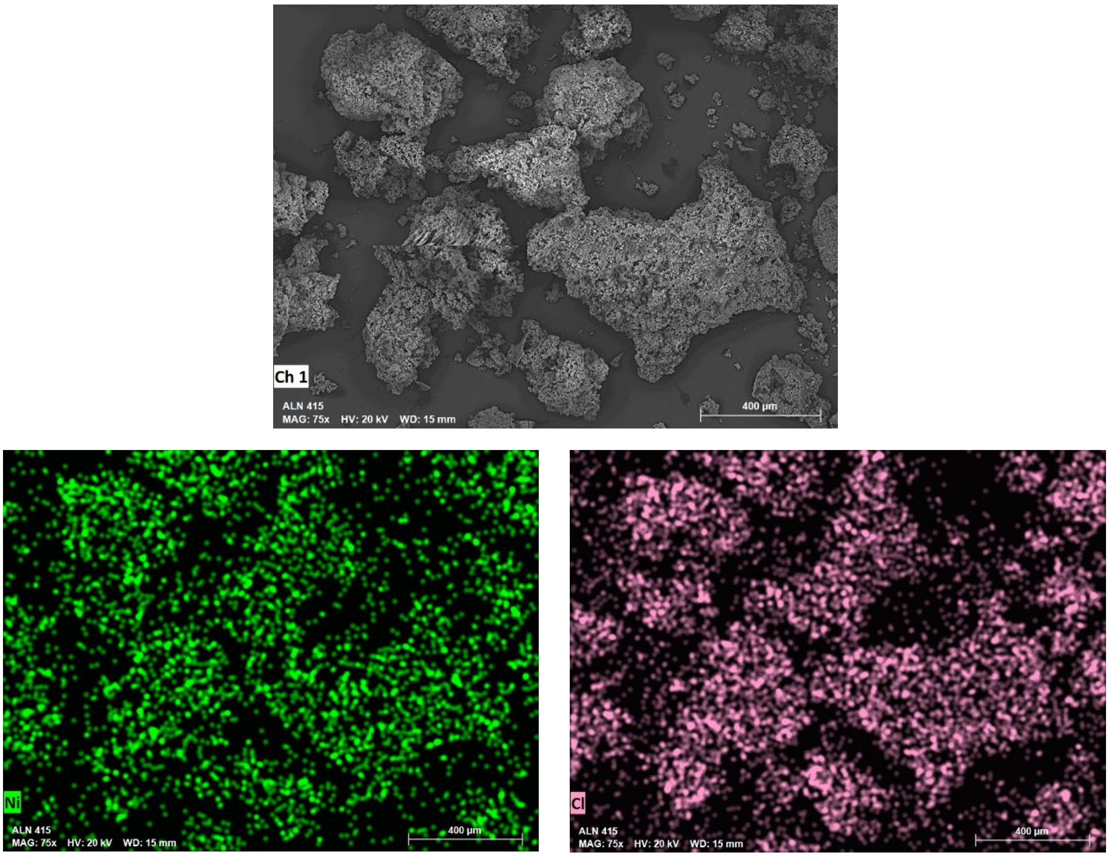

Figure S31: SEM-EDX images of Ni@Phen-COF (Ni-green, Cl-pink).

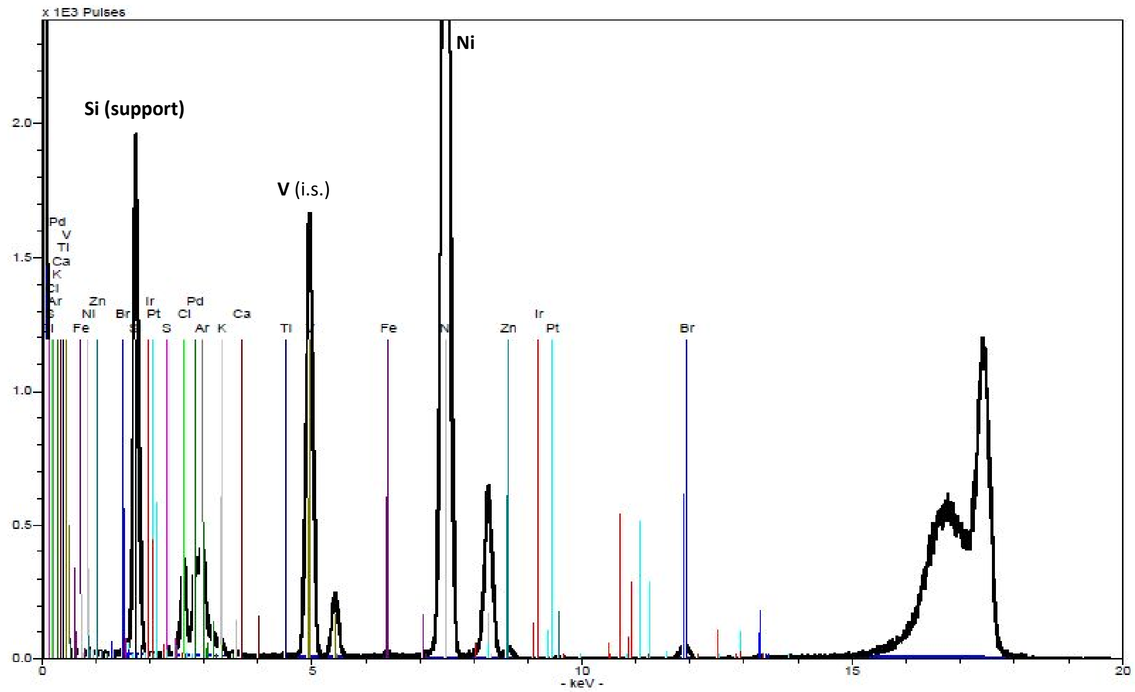

Figure S32: Quantitative TXRF spectra of Ni@Phen-COF (2.9 % of Ni).

Synthesis of Ir@Phen-COF: in a 100 mL round bottom-flask, 140 mg of **Phen-COF** were suspended on 50 mL of dry acetonitrile and 1 mL of aqueous acetic acid 6 M during 30 minutes with an ultrasound bath. Then, $[(dF(CF_3)ppy)_2\text{-Ir-}\mu\text{-Cl}]_2$ (33 mg, 0.022 mmol) was added. The mixture was stirred at 70 ºC for 16 hours, yielding a yellow powder that was isolated by filtration and washed with hot DMF, acetonitrile and THF. The resulting powder was washed with acetone using Soxhlet extraction, then filtered and dried first at room temperature under vacuum for 12 h, and then at 100 ˚C for 2 h to afford an orange powder, **Ir@Phen-COF**. 143 mg obtained.

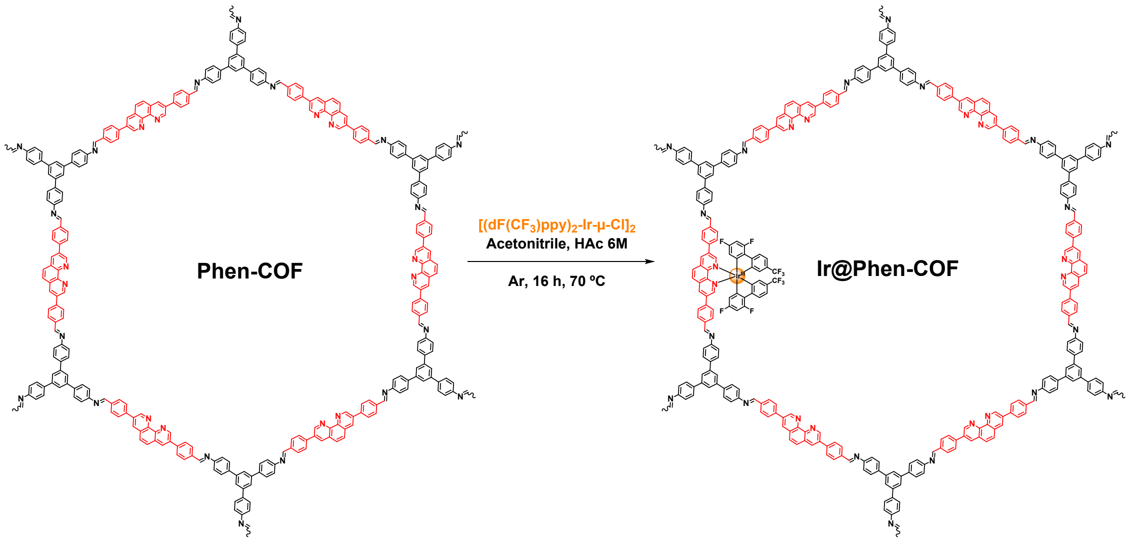

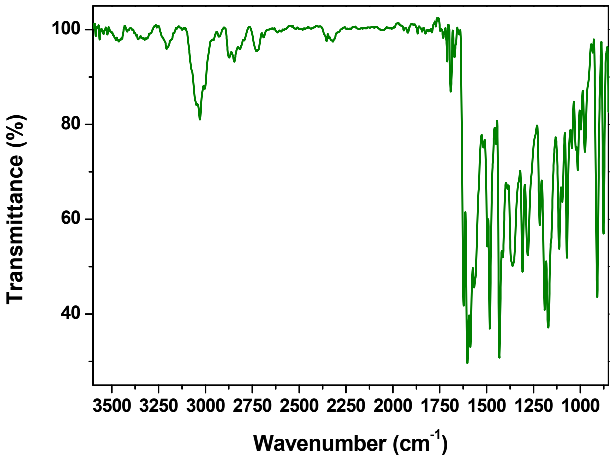

Figure S33: Fourier-Transformed Infrared Spectra of Ir@Phen-COF.

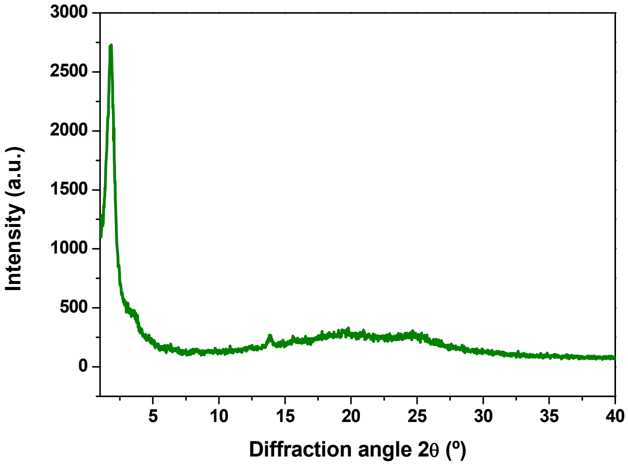

Figure S34: PXRD pattern of Ir@Phen-COF.

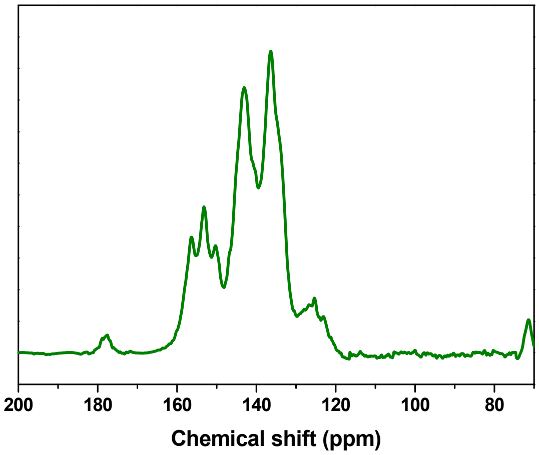

Figure S35: ^{13}C-NMR-CP-MAS spectra of Ir@Phen-COF.

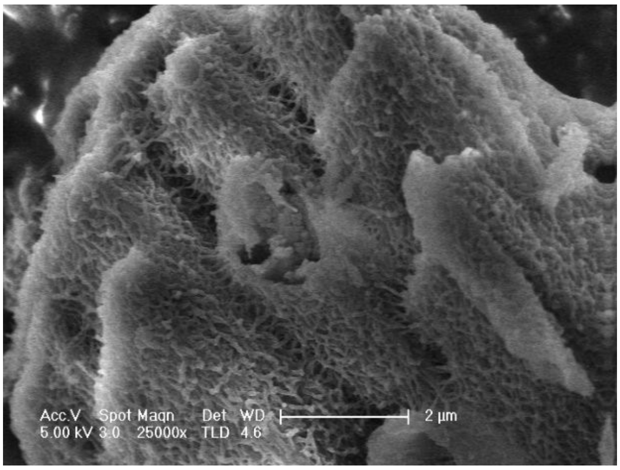

Figure S36: FE-SEM image of Ir@Phen-COF.

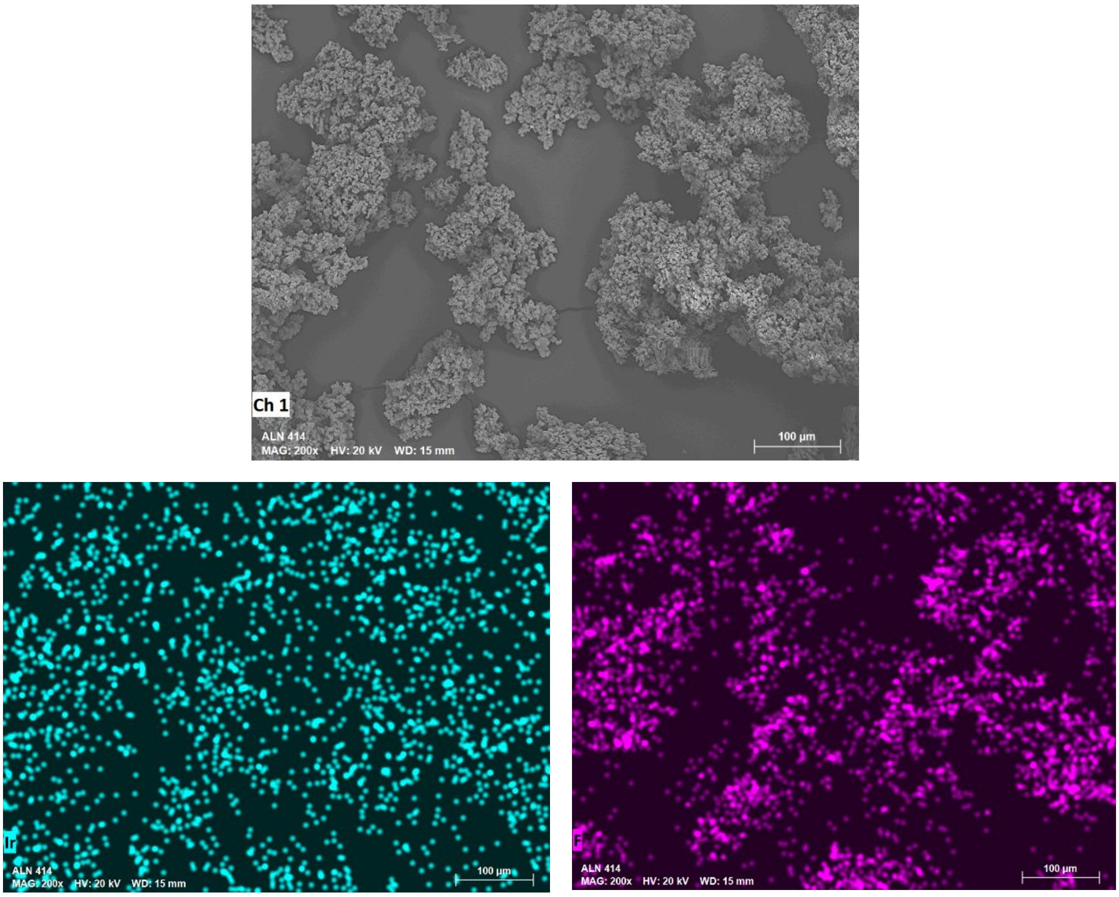

Figure S37: SEM-EDX images of Ir@Phen-COF (Ir-blue, F-purple).

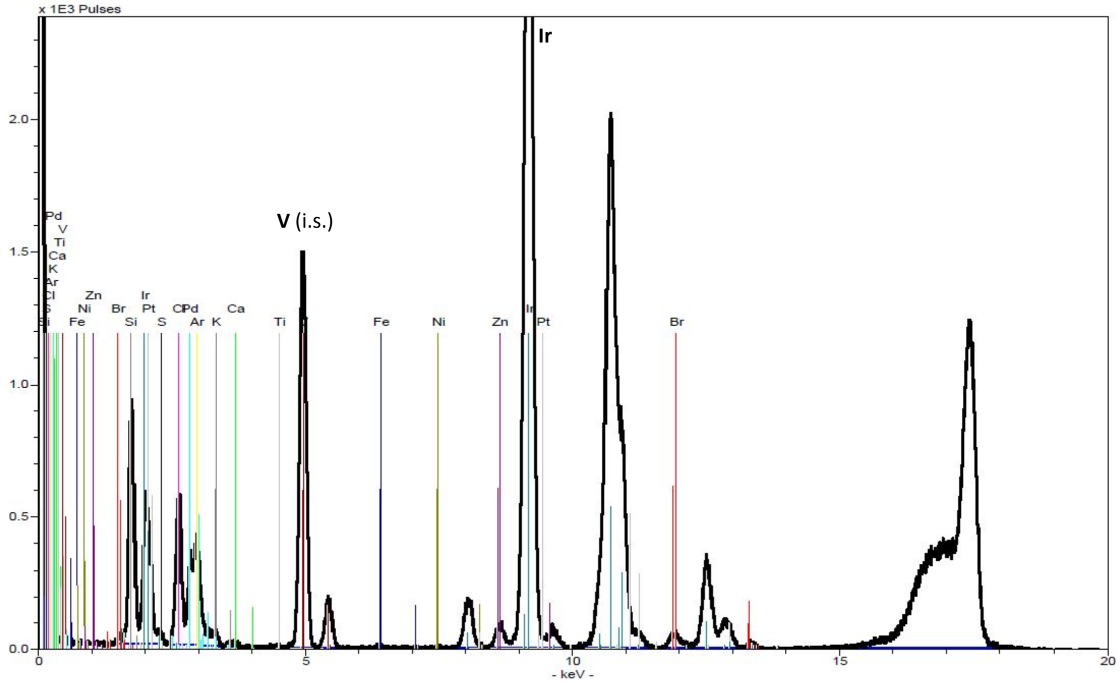

Figure S38: Quantitative TXRF spectra of Ir@Phen-COF (3.9 % of Ir).

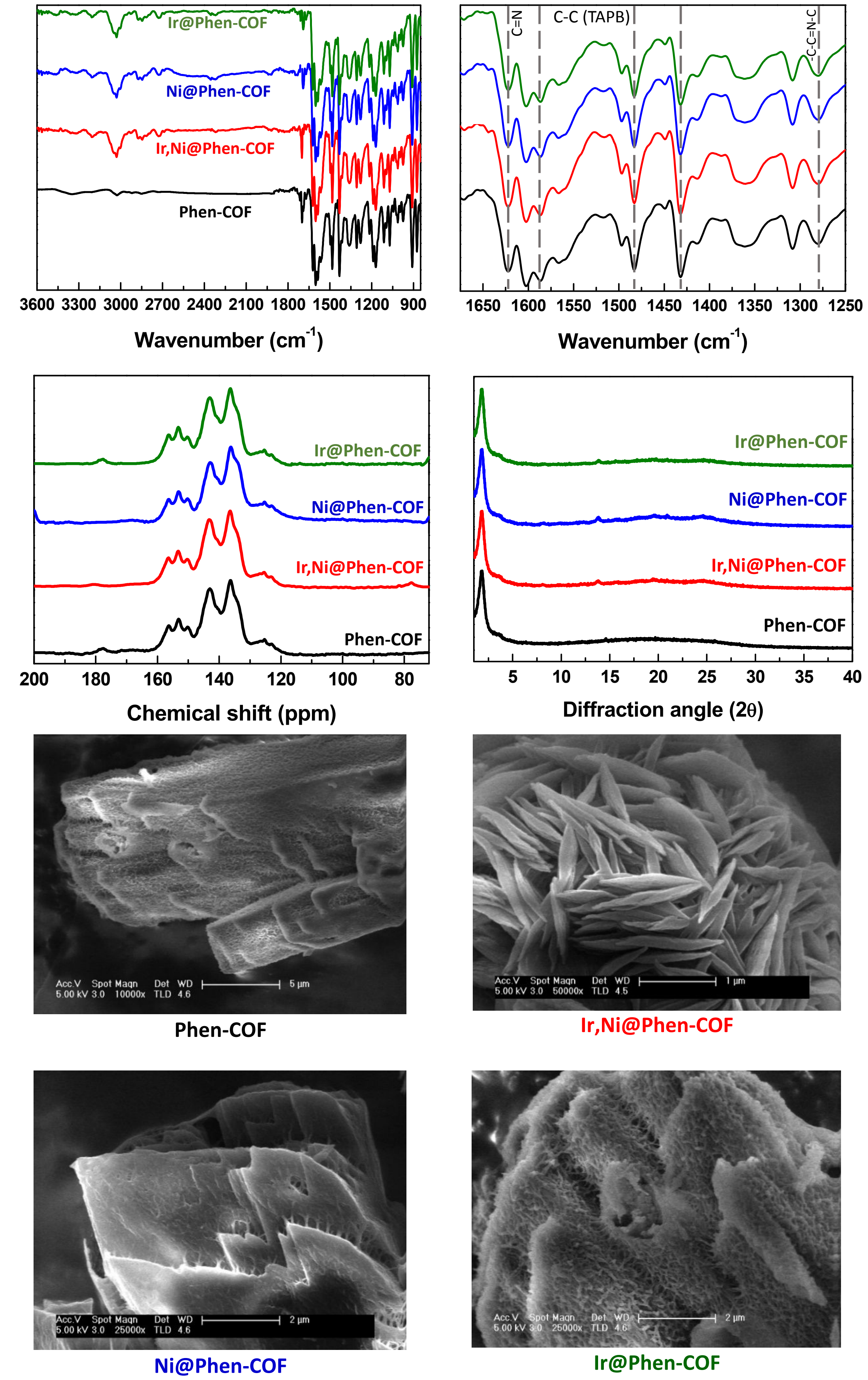

Figure S39: Comparison of FTIR, PXRD, ^{13}C-NMR-CP-MAS and SEM of pristine and functionalized Phen-COFs.

Synthesis of PF-Crystalline Laminar COF

A 45 mL solvothermal reactor was charged with **TAPB** (100.0 mg, 0.285 mmol), BTCA (46.0 mg, 0.285 mmol), 8.9 mL of a mixture of anhydrous dioxane : mesitylene (1:9) and 0.9 mL of 6 M acetic acid in water. The reactor was heated at 120 ˚C for 72 h, yielding a brown solid which was isolated by filtration and washed with DMSO, methanol and THF. The resulting powder was immersed in anhydrous THF for 24 h and dried first at room temperature under vacuum for 12 h, and then at 100 ˚C for 2 h to afford a brown powder. Its characterization agreed well with previous works.[6,7]

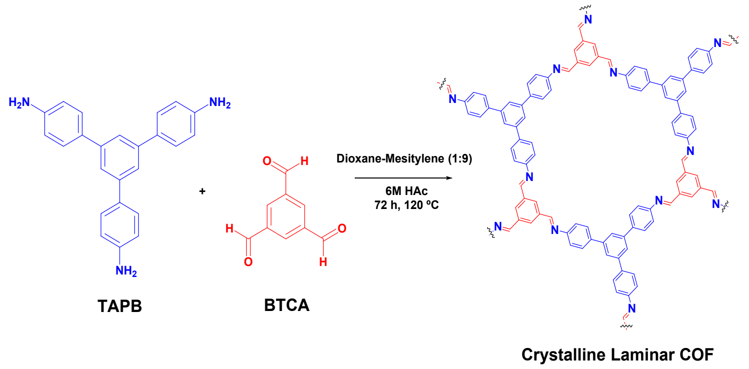

Then, an analogous post-functionalization protocol than the used for the synthesis of **Ir,Ni@Phen-COF** was followed. In a 100 mL round bottom-flask, 140 mg of **Crystalline Laminar COF** were suspended on 50 mL of dry acetonitrile and 1 mL of aqueous acetic acid 6 M during 30 minutes with an ultrasound bath. Then, [(dF(CF$_3$)ppy)$_2$-Ir-µ-Cl]$_2$ (33 mg, 0.022 mmol) and NiCl$_2$·glyme (19.6 mg, 0.089 mmol) were added. The mixture was stirred at 70 ºC for 16 hours, yielding a yellow powder that was isolated by filtration and washed with hot DMF, acetonitrile and THF. The resulting powder was washed with acetone using Soxhlet extraction, then filtered and dried first at room temperature under vacuum for 12 h, and then at 100 ˚C for 2 h to afford an orange powder, **PF-Crystalline Laminar COF**. 138 mg obtained. In order to determine the quantity of Ir and Ni incorporated into the material, quantitative TXRF analyses were performed. Very low quantities of both Ir and Ni can be observed.

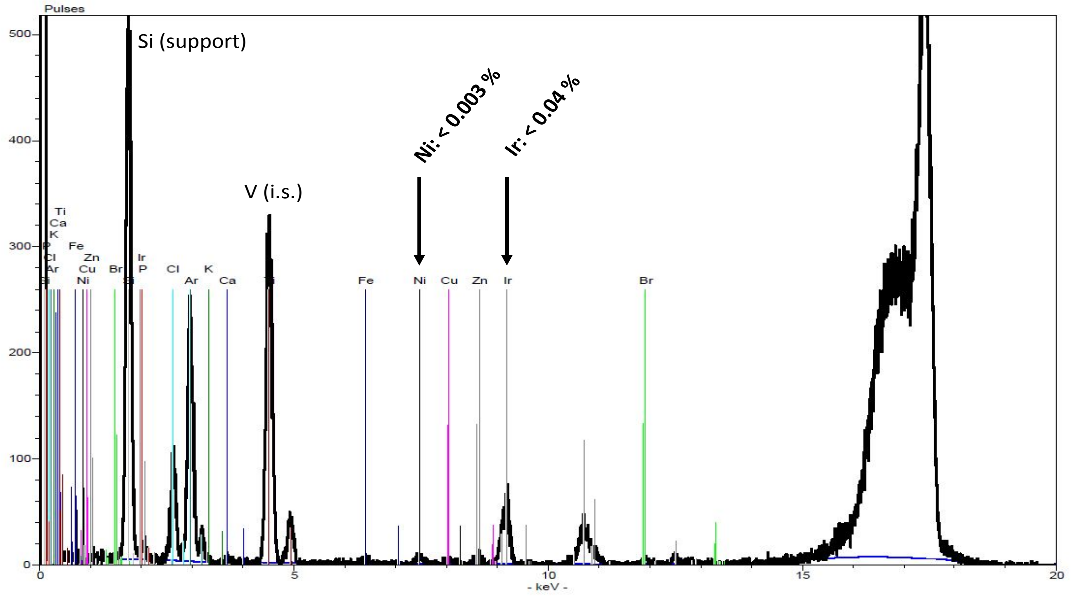

Figure S40: Quantitative TXRF analyses for PF-Crystalline Laminar COF.

General procedure for the light-mediated cross-coupling between potassium benzyltrifluoroborate and aryl bromides

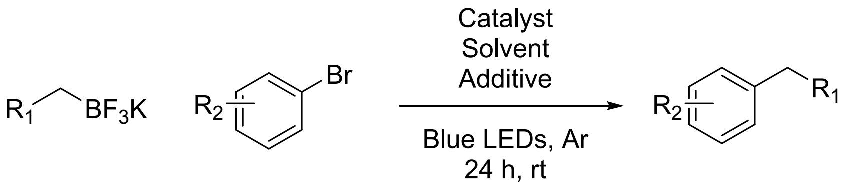

An oven-dried 10 mL vial equipped with a magnetic stir bar was charged with catalyst (1.2 mg), corresponding potassium trifluoroborate salt **2** (0.15 mmol) and the corresponding aryl bromide **3** (0.075 mmol). Then, 950 µL of dry acetone and 50 µL of dry MeOH were added. Finally, 2,6-lutidine (30.5 µL, 0.26 mmol) was added. The vial was closed with a rubber septum and the reaction mixture was degassed by three cycles vacuum / Ar of "freeze-pump-thaw". Then the vial was placed on a blue light LED photoreactor (450 nm) with an Ar balloon and the reaction mixture was stirred at 25 ˚C. The reaction was monitored by TLC and ^{1}H-NMR. The yield was determined by ^{1}H-NMR using nitromethane as internal standard.

Table S2: Screening of the reaction.

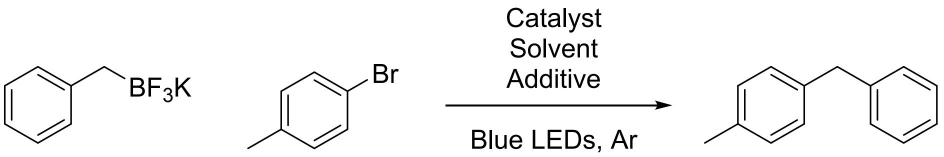

2a **3a** **4a**

Entry	Catalyst (mg)	Solvent	Additive (equiv.)	NMR Yield (%)
1	**Ir,Ni@Phen-COF** (1.2 mg)	Acetone-MeOH 95:5	2,6-lutidine (3.5)	51
2	**Ir,Ni@Phen-COF** (1.2 mg)	THF	2,6-lutidine (3.5)	6
3	**Ir,Ni@Phen-COF** (1.2 mg**)**	Acetonitrile	2,6-lutidine (3.5)	30
4	**Ir,Ni@Phen-COF** (1.2 mg)	DCM	2,6-lutidine (3.5)	0
5	**Ir,Ni@Phen-COF** (1.2 mg)	DMF	2,6-lutidine (3.5)	35
6	**Ir,Ni@Phen-COF** (1.2 mg)	Toluene	2,6-lutidine (3.5)	0
7	**Ir,Ni@Phen-COF** (1.2 mg)	Acetone	2,6-lutidine (3.5)	40
8	**Ir,Ni@Phen-COF** (1.2 mg)	MeOH	2,6-lutidine (3.5)	23
9	**Ir,Ni@Phen-COF** (1.2 mg)	EtOH	2,6-lutidine (3.5)	21
10	**Ir,Ni@Phen-COF** (1.2 mg)	Acetone-MeOH 95:5	-	3
11	**Ir,Ni@Phen-COF** (1.2 mg)	Acetone-MeOH 95:5	2,6-lutidine (3.5)	90 [a]
12	**Ir,Ni@Phen-COF** (1.2 mg)	Acetone-MeOH 95:5	DIPEA (3.5)	76
13	**Ir,Ni@Phen-COF** (1.2 mg)	Acetone-MeOH 95:5	Cs_2CO_3 (3.5)	23
14	**Ir,Ni@Phen-COF** (1.2 mg)	Acetone-MeOH 95:5	K_2CO_3 (3.5)	12
15	**Ir,Ni@Phen-COF** (1.2 mg)	Acetone-MeOH 95:5	Na_3PO_4 (3.5)	10
16	**Ir,Ni@Phen-COF** (1.2 mg)	Acetone-MeOH 95:5	2,6-lutidine (1)	54
17	**Ir,Ni@Phen-COF** (1.2 mg)	Acetone-MeOH 95:5	2,6-lutidine (2)	79
18	**Ir,Ni@Phen-COF** (1.2 mg)	Acetone-MeOH 95:5	2,6-lutidine (5)	70
19	**Ir,Ni@Phen-COF** (1.2 mg)	Acetone-MeOH 95:5	2,6-lutidine (7)	43
20	**Ir,Ni@Phen-COF** (0.6 mg)	Acetone-MeOH 95:5	2,6-lutidine (3.5)	64
21	**Ir,Ni@Phen-COF** (2.4 mg)	Acetone-MeOH 95:5	2,6-lutidine (3.5)	40

22	-	Acetone-MeOH 95:5	2,6-lutidine (3.5)	0
23	**Phen-COF** (1.2 mg)	Acetone-MeOH 95:5	2,6-lutidine (3.5)	0
24	**Ni@Phen-COF** (1.2 mg)	Acetone-MeOH 95:5	2,6-lutidine (3.5)	0
25	**Ir@Phen-COF** (1.2 mg)	Acetone-MeOH 95:5	2,6-lutidine (3.5)	0
26	**Ir@Phen-COF** (0.6 mg) **Ni@Phen-COF** (0.6 mg)	Acetone-MeOH 95:5	2,6-lutidine (3.5)	0
27	**Ir,Ni@Phen-COF** (1.2 mg) **(Dark conditions)**	Acetone-MeOH 95:5	2,6-lutidine (3.5)	0
28	**Crystalline Laminar COF** (1.2 mg)	Acetone-MeOH 95:5	2,6-lutidine (3.5)	0
29	**[(dF(CF$_3$)ppy)$_2$-Ir-μ-Cl]$_2$** (0.3 mol%) **NiCl$_2$·glyme** (0.7 mol%)	Acetone-MeOH 95:5	2,6-lutidine (3.5)	0
30	**[Ir(dF(CF$_3$)ppy)$_2$(bpy)]PF$_6$** (0.3 mol%) **[NiCl$_2$(phen)]** (0.7 mol%)	Acetone-MeOH 95:5	2,6-lutidine (3.5)	23
31	**Amorphous Ir,Ni@Phen-COF** [b]	Acetone-MeOH 95:5	2,6-lutidine (3.5)	87

[a] This result was obtained when the reaction was sealed using a typical rubber septum instead of a PTFE-rubber septum. The reproducibility and the yields were significantly increased. [b] This material was obtained after the functionalization of the Phen-COF synthesized following the procedure presented in entry 14 of Table S1.

Variation of catalytic activity of the material depending on metal loading and Ir/Ni ratio

For the study of the influence of metal loading in the catalytic activity under the optimal conditions, a series of 6 different Ir,Ni@Phen-COF were obtained variating the amount of metal precursors on the post-functionalization stage. The results obtained appear in the following table:

Table S3: Study of the influence of metal loading on the catalytic activity.

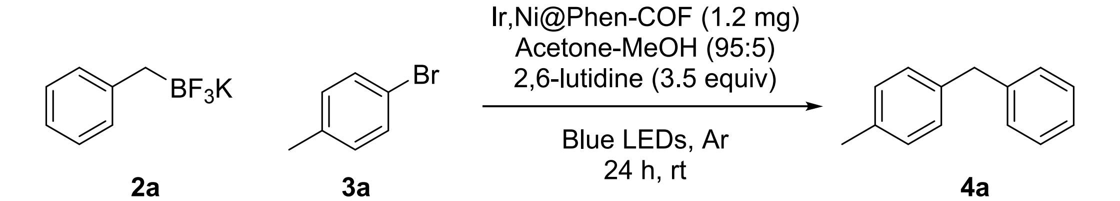

Catalyst	% Ir	% Ni	Molar Ratio Ni/Ir	NMR yield	TON (Ir/Ni)
Ir,Ni@Phen-COF	4.0	2.8	2:1	90	270/117
Ir,Ni@Phen-COF	7.0	4.2	2:1	87	149/76
Ir,Ni@Phen-COF	2.0	1.4	2:1	25	150/65
Ir,Ni@Phen-COF	2.2	2.8	4:1	42	229/55
Ir,Ni@Phen-COF	9.0	2.8	1:1	46	61/60

Substrate Scope

1-benzyl-4-methylbenzene (4a) [8]

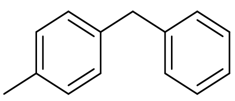

^{1}**H-NMR** (300 MHz, $CDCl_3$) δ 7.29 – 7.08 (m, 8 H), 3.93 (s, 2 H), 2.30 (s, 3 H).

Diphenylmetane (4b) [9]

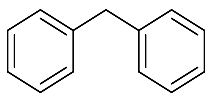

^{1}**H-NMR** (300 MHz, $CDCl_3$) δ 7.32-7.19 (m, 10H), 4.00 (s, 2H) ppm.

1-benzyl-4-(tert-butyl)benzene (4c) [10]

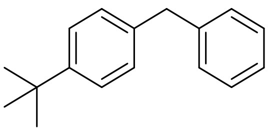

^{1}**H-NMR** (300 MHz, $CDCl_3$) δ 7.31–7.28 (m, 4H), 7.22–7.18 (m, 3H), 7.13–7.10 (m, 2H), 3.95 (s, 2H), 1.29 (s, 9H) ppm.

1-benzyl-4-methoxybenzene (4d) [8]

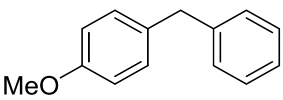

^{1}**H-NMR** (300 MHz, $CDCl_3$) δ 7.35 – 7.09 (m, 8 H), 6.84 – 6.81 (m, 2 H), 3.93 (s, 2 H), 3.77 (s, 3 H).

4-benzylbenzonitrile (4e) [11]

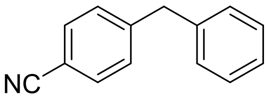

^{1}**H-NMR** (300 MHz, $CDCl_3$) δ 7.58 (d, *J* = 8.3 Hz, 2H), 7.29 (m, 5H), 7.17 (d, *J* = 8.3 Hz, 2H), 4.04 (s, 2H) ppm.

4-benzylbenzaldehyde (4f) [3]

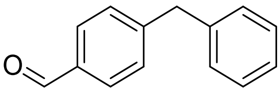

^{1}**H-NMR** (300 MHz, $CDCl_3$) δ 9.98 (s, 1H), 7.81 (d, *J* = 8.0 Hz, 2H), 7.36 (d, *J* = 8.0 Hz, 2H), 7.31 (dd, *J* = 7.5, 7.0 Hz, 2H), 7.24 (t, *J* = 7.0 Hz, 1H), 7.19 (d, *J* = 7.5 Hz, 2H), 4.06 (s, 2H) ppm.

1-benzyl-3,5-bis(trifluoromethyl)benzene (4g) [12]

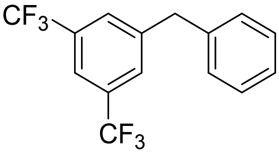

^{1}**H-NMR** (300 MHz, $CDCl_3$) δ 7.72 (s, 1H), 7.63 (s, 2H), 7.35-7.31 (m, 2H), 7.28 – 7.24 (m, 1H), 7.18 – 7.16 (m, 2H), 4.09 (s, 2H) ppm.

1-benzyl-4-vinylbenzene (4h) [13]

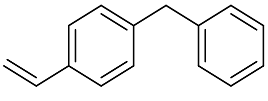

^{1}**H-NMR** (300 MHz, $CDCl_3$) δ 7.33 (d, *J* = 7.8 Hz, 2H), 7.28 (d, *J* = 7.8 Hz, 2H), 7.19-7.14 (m, 5H), 6.69 (dd, *J* = 17.4, 10.8 Hz, 1H), 5.70 (d, *J* = 17.4 Hz, 1H), 5.19 (d, *J* = 10.8 Hz, 1H), 3.97 (s, 2H) ppm.

Recyclability experiment

An oven-dried 10 mL vial equipped with a magnetic stir bar was charged with catalyst (5 mg), corresponding potassium trifluoroborate salt **2a** (0.15 mmol) and the corresponding aryl bromide **3b** (0.075 mmol). Then, 950 μL of dry acetone and 50 μL of dry MeOH were added. Finally, 2,6-lutidine (30.5 μL, 0.26 mmol) was added. The vial was closed with a rubber septum and the reaction mixture was degassed by three cycles vacuum / Ar of "freeze-pump-thaw". Then the vial was placed on a blue light LED photoreactor (450 nm) with an Ar balloon and the reaction mixture was stirred at 25 ˚C. After 26 h of reaction, an aliquot is analyzed by NMR to determine the yield using nitromethane as internal standard. Then, the reaction crude is centrifuged and washed with acetone 3 times. The catalyst is recovered as a yellow powder and was dried under vacuum during 2 hours before the next catalytic run. When the reaction was catalyzed by **Ir,Ni@Phen-COF**, 7 catalytic runs were performed, producing 0.92 mmol of diphenylmethane **4b.**

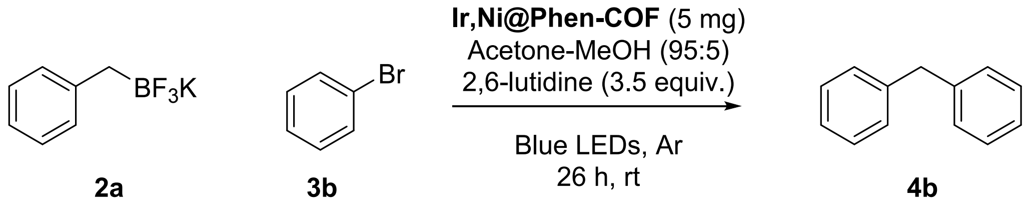

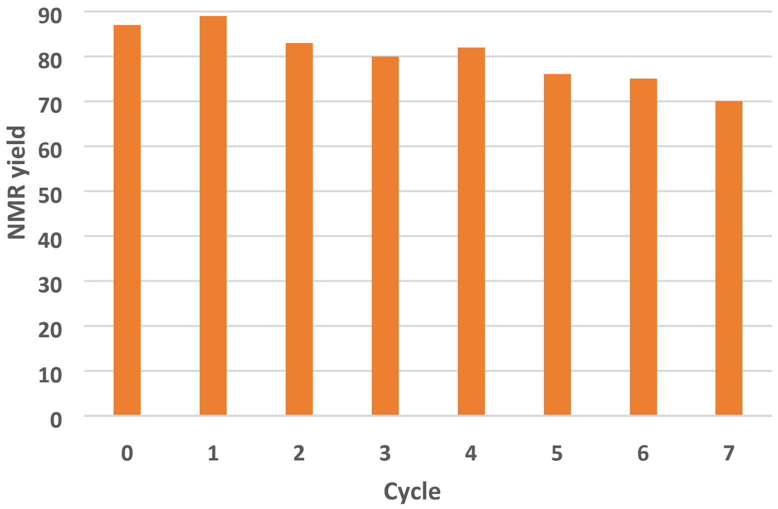

Figure S41: Recyclability of Ir,Ni@Phen-COF.

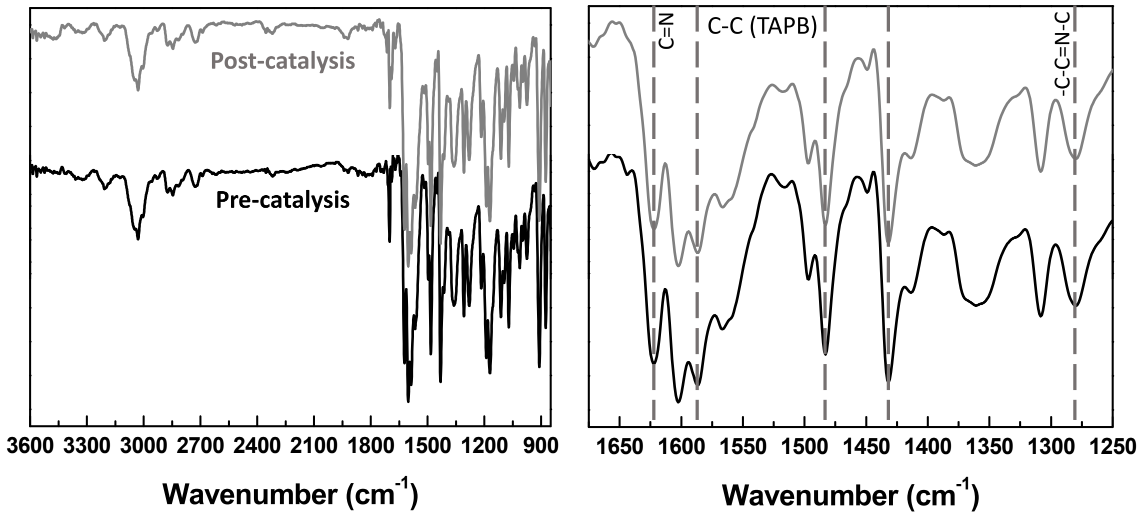

Figure S42: Comparison of FTIR spectra before and after catalysis.

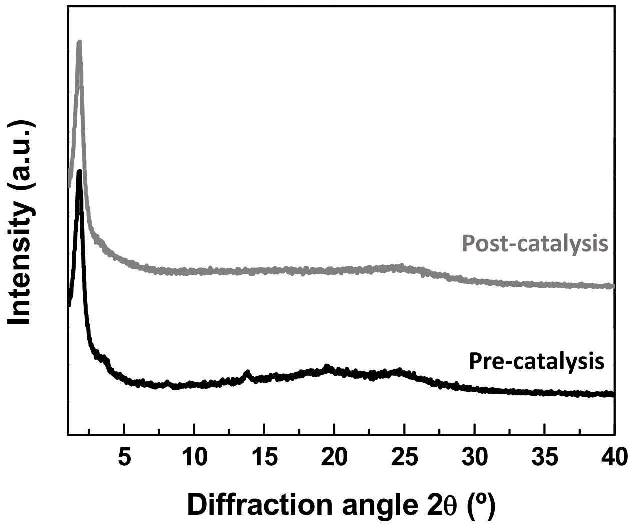

Figure S43: Comparison of PXRD patterns before and after catalysis.

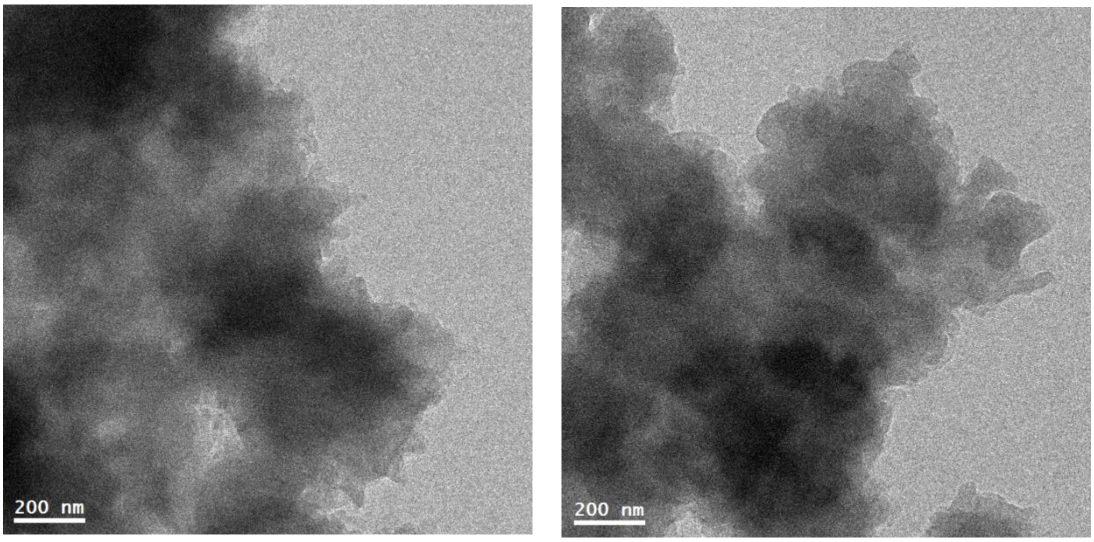

Figure S44: Comparison of TEM images before (left) and after catalysis (right).

Leaching experiments

Once the first catalytic run was completed, the reaction was filtered in order to remove the catalyst. The supernatant was transferred to an oven-dried 10 mL vial equipped with a magnetic stir bar. The vial was closed with a PTFE / rubber septum and the reaction mixture was degassed by three cycles vacuum / oxygen of “freeze-pump-thaw”. Then the vial was placed on a blue light LED photoreactor (450 nm) and the reaction mixture was stirred at 25 ˚C. The reaction was monitored by ^{1}H-NMR, observing that it did not advance further for the next 16 h. In addition, ICP-AES measurements confirmed that no detectable Ir and Ni quantities were observed in the supernatant.

Procedure for the light-mediated cross-coupling between potassium *tert*-butoxymethyltrifluoroborate and aryl bromides.

An oven-dried 10 mL vial equipped with a magnetic stir bar was charged with catalyst (1.2 mg), corresponding potassium (*tert*-butoxymethyl)trifluoroborate **5** (0.15 mmol) and the corresponding aryl bromide **3e** (0.075 mmol). Then, 950 µL of dry acetone and 50 µL of dry MeOH were added. Finally, 2,6-lutidine (30.5 µL, 0.26 mmol) was added. The vial was closed with a rubber septum and the reaction mixture was degassed by three cycles vacuum / Ar of “freeze-pump-thaw”. Then the vial was placed on a blue light LED photoreactor (450 nm) with an Ar balloon and the reaction mixture was stirred at 25 ˚C. The reaction was monitored by TLC and ^{1}H-NMR. The yield was determined by ^{1}H-NMR using nitromethane as internal standard.

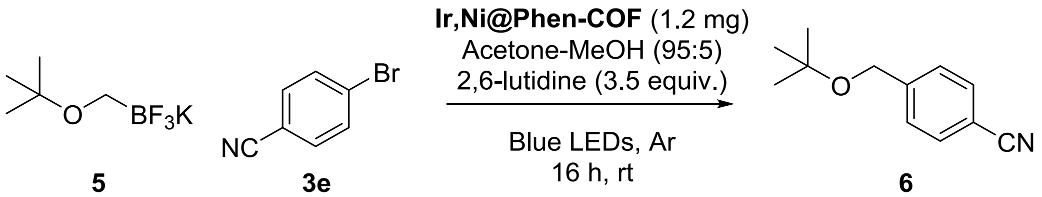

5 **3e** **6**

Entry	Catalyst	Yield (%)
1	**Ir,Ni@Phen-COF** (1.2 mg)	76
2	**Ni@Phen-COF** (1.2 mg)	0
3	**Ir@Phen-COF** (1.2 mg)	0
4	**Ni@Phen-COF** (0.6 mg) and **Ir@Phen-COF** (0.6 mg)	0
5	$[Ir(dF(CF_3)ppy)_2(bpy)]PF_6$ (0.3 mol%)<br>$[NiCl_2(phen)]$ (0.7 mol%)	12
6	**Ir,Ni@Phen-COF** (1.2 mg) (Dark conditions)	0
7	-	0

4-(*tert*-butoxymethyl)benzonitrile (6) [14]

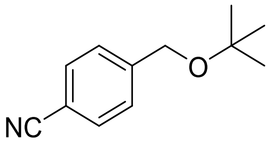

^{1}H-NMR (300 MHz, $CDCl_3$) δ 7.62 (d, *J* = 8.1 Hz 2H), 7.46 (d, *J* = 8.0 Hz, 2H), 4.5 (s, 2H), 1.31 (s, 9H) ppm.

Procedure for the light-mediated cross-coupling between organic silicates and aryl bromides.

An oven-dried 10 mL vial equipped with a magnetic stir bar was charged with catalyst (2.5 mg), radical precursor **7** (102 mg, 0.225 mmol) and the corresponding aryl bromide **3e** (27.3 mg, 0.15 mmol). Then, 3 µL of dry DMF were added. The vial was closed with a rubber septum and the reaction mixture was degassed by three cycles vacuum / Ar of "freeze-pump-thaw". Then the vial was placed on a blue light LED photoreactor (450 nm) with an Ar balloon and the reaction mixture was stirred at 25 °C. The reaction was monitored by TLC and ^{1}H-NMR. When total conversion was observed, the crude was filtered through membrane filter and extracted with 3 x 15 mL of diethyl ether and aqueous saturated solution of $NaHCO_3$. The organic phases were dried over Na_2SO_4, filtered, and evaporated under reduced pressure. The yield was determined by ^{1}H-NMR using nitromethane as internal standard.

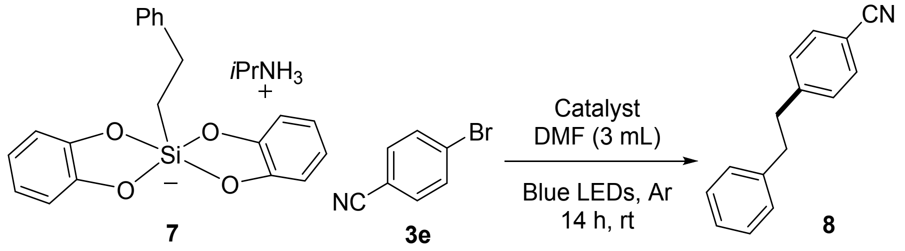

Entry	Catalyst	Conversion (%)
1	**Ir,Ni@Phen-COF** (2.5 mg)	92
2	**Ni@Phen-COF** (2.5 mg)	0
3	**Ir@Phen-COF** (2.5 mg)	0
4	**Ni@Phen-COF** (1.2 mg) and **Ir@Phen-COF** (1.2 mg)	0
5	$[Ir(dF(CF_3)ppy)_2(bpy)]PF_6$ (0.3 mol%)<br>$[NiCl_2(phen)]$ (0.7 mol%)	26
6	**Ir,Ni@Phen-COF** (2.5 mg) (Dark conditions)	0
7	-	0

4-phenethylbenzonitrile (8) [15]

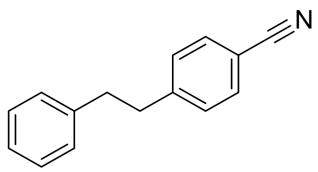

^{1}H-NMR (300 MHz, $CDCl_3$) δ 7.54 (d, *J* = 7.8 Hz, 2H), 7.18-7.29 (m, 5H), 7.12 (d, *J* = 7.8 Hz, 2H), 2.90-2.99 (m, 4H) ppm.

Procedure for the light-mediated decarboxylative arylation of α-aminoacids with aryl bromides.

An oven-dried 10 mL vial equipped with a magnetic stir bar was charged with catalyst (2.5 mg), Cs_2CO_3 (73 mg, 0.225 mmol) radical precursor **9** (48.4 mg, 0.225 mmol) and the corresponding aryl bromide **3e** (27.3 mg, 0.15 mmol). Then, 4 μL of dry DMF were added. The vial was closed with a rubber septum and the reaction mixture was degassed by three cycles vacuum / Ar of "freeze-pump-thaw". Then the vial was placed on a blue light LED photoreactor (450 nm) with an Ar balloon and the reaction mixture was stirred at 25 ˚C. The reaction was monitored by TLC and ^{1}H-NMR. When total conversion was observed, the crude was filtered through membrane filter and extracted with 3 x 15 mL of diethyl ether and aqueous saturated solution of $NaHCO_3$. The organic phases were dried over Na_2SO_4, filtered, and evaporated under reduced pressure. The yield was determined by ^{1}H-NMR using nitromethane as internal standard.

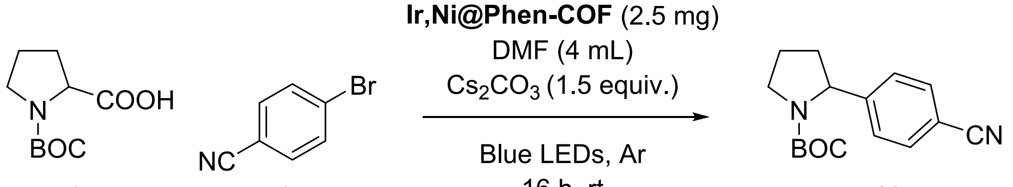

9 **3e** **10**

Entry	Catalyst	Conversion (%)
1	**Ir,Ni@Phen-COF** (1.2 mg)	86
2	**Ni@Phen-COF** (1.2 mg)	0
3	**Ir@Phen-COF** (1.2 mg)	0
4	**Ni@Phen-COF** (0.6 mg) and **Ir@Phen-COF** (0.6 mg)	0
5	$[Ir(dF(CF_3)ppy)_2(bpy)]PF_6$ (0.3 mol%) $[NiCl_2(phen)]$ (0.7 mol%)	58
6	**Ir,Ni@Phen-COF** (1.2 mg) (Dark conditions)	0
7	-	0

***tert*-butyl 2-(4-cyanophenyl)pyrrolidine-1-carboxylate (10)** [16]

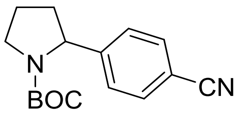

Product **10** gives two sets of NMR signals, due to the presence of rotamers on the tertiary amide moiety.

^{1}H-NMR (300 MHz, $CDCl_3$) δ 7.60 (d, *J* = 8.0 Hz, 2H), 7.28 (d, *J* = 8.0 Hz, 2H), 4.95-4.80 (m, 1H), 3.70-3.61 (m, 2H), 2.40-2.36 (m, 1H), 1.89-1.86 (m, 2H), 1.78-1.75 (m, 1H), 1.44 (s, 3H), 1.18 (s, 6H).

Benchmarking of catalytic results.

Table S4: Comparison of the catalytic outputs (TONs) reached using Ir,Ni@Phen-COF, homogeneous analogues and reported data in literature.

Entry	Reaction	Catalyst	TON [a]	REF
1	**2+3a**	**Ir,Ni@Phen-COF**	129 (424) [b]	This work
2	**2+3a**	$[Ir(dF(CF_3)ppy)_2(bpy)]PF_6$, $[NiCl_2(phen)]$	33	This work
3	**2+3a**	$[Ir(dF(CF_3)ppy)_2(bpy)]PF_6$, $Ni(COD)_2$/dtbpy	30	3
4	**5+3e**	**Ir,Ni@Phen-COF**	109	This work
5	**5+3e**	$[Ir(dF(CF_3)ppy)_2(bpy)]PF_6$, $[NiCl_2(phen)]$	17	This work
6	**5+3e**	$[Ir(dF(CF_3)ppy)_2(bpy)]PF_6$, $NiCl_2$·glyme/dtbpy	30	17
7	**7+3e**	**Ir,Ni@Phen-COF**	131	This work
8	**7+3e**	$[Ir(dF(CF_3)ppy)_2(bpy)]PF_6$, $[NiCl_2(phen)]$	37	This work
9	**7+3e**	$[Ir(dF(CF_3)ppy)_2(bpy)]PF_6$, $Ni(COD)_2$/dtbpy	28	18
10	**9+3e**	**Ir,Ni@Phen-COF**	123	This work
11	**9+3e**	$[Ir(dF(CF_3)ppy)_2(bpy)]PF_6$, $[NiCl_2(phen)]$	82	This work
12	**9+3e**	$[Ir(dF(CF_3)ppy)_2(bpy)]PF_6$, $NiCl_2$·glyme/dtbpy	9.3	19

[a] Calculated based on Ni catalyst loading. [b] After 8 catalytic runs.

PXRD Simulation

Table S5. Atomistic coordinates for simulated PXRD pattern of Phen-COF.

Atom	x/a	y/b	z/c	Symmetry operation
C1	0	0.1734	-0.1048	x, y, z
C2	0	0.1805	-0.083	x, y, z
C3	0	0.2162	0.0613	x, y, z
C4	0	0.2647	0.2067	x, y, z
C5	0	0.3189	0.3325	x, y, z
C6	0	0.3263	0.354	x, y, z
C7	0	0.2026	0.0201	x, y, z
C8	0	0.1938	-0.0414	x, y, z
C9	0	0.1803	-0.1252	x, y, z
C10	0	0.1735	-0.0625	x, y, z
C11	0	0.2026	0.061	x, y, z
C12	0	0.258	0.2274	x, y, z
C13	0	0.3052	0.3327	x, y, z
C14	0	0.2008	-0.0617	x, y, z
C15	0	0.2162	0.0202	x, y, z
C16	0	0.223	0.0406	x, y, z
C17	0	0.1941	-0.0823	x, y, z
C18	0	0.2982	0.3125	x, y, z
C19	0	0.2648	0.2478	x, y, z
C20	0	0.2784	0.2069	x, y, z
C21	0	0.3254	0.3117	x, y, z
C22	0	0.1801	-0.0419	x, y, z
C23	0	0.1958	0.0405	x, y, z
C24	0	0.3048	0.2917	x, y, z
C25	0	0.2784	0.2478	x, y, z
C26	0	0.3184	0.2915	x, y, z
C27	0	0.2852	0.2273	x, y, z
C28	0	0.347	0.3741	x, y, z
C29	0	0.3614	0.3738	x, y, z
C30	0	0.4515	0.3571	x, y, z
C31	0	0.4241	0.3563	x, y, z
C32	0	0.3889	0.3733	x, y, z
C33	0	0.3399	0.3539	x, y, z
C34	0	0.3686	0.3941	x, y, z
C35	0	0.4444	0.3773	x, y, z
C36	0	0.3819	0.3529	x, y, z
C37	0	0.4312	0.3361	x, y, z
C38	0	0.3683	0.3532	x, y, z
C39	0	0.4448	0.3365	x, y, z
C40	0	0.3822	0.3938	x, y, z

C41	0	0.4308	0.3769	x, y, z
C42	0	0.1598	-0.1054	x, y, z
N43	0	0.2009	-0.0205	x, y, z
C44	0	0.1951	-0.001	x, y, z
N45	0	0.2979	0.2707	x, y, z
C46	0	0.2853	0.2695	x, y, z
N47	0	0.4029	0.3736	x, y, z
C48	0	0.4098	0.3553	x, y, z
C49	0	0.2574	0.1852	x, y, z
C50	0	0.264	0.1643	x, y, z
N51	0	0.2572	0.1446	x, y, z
C52	0	0.2439	0.1442	x, y, z
C53	0	0.2369	0.1648	x, y, z
C54	0	0.2437	0.1851	x, y, z
C55	0	0.2233	0.1647	x, y, z
C56	0	0.2165	0.1443	x, y, z
C57	0	0.2233	0.1239	x, y, z
C58	0	0.2371	0.1237	x, y, z
N59	0	0.2435	0.1036	x, y, z
C60	0	0.2371	0.0836	x, y, z
C61	0	0.2234	0.083	x, y, z
C62	0	0.2166	0.1035	x, y, z
C63	0	0.466	0.3575	x, y, z
C64	0	0.4731	0.3374	x, y, z
N65	0	0.4864	0.3377	x, y, z
C66	0	0.4932	0.3575	x, y, z
C67	0	0.4864	0.3782	x, y, z
C68	0	0.4728	0.378	x, y, z
C69	0	0.4932	0.3986	x, y, z
C49	0	0.7426	0.1852	-x, 1-y, z
C50	0	0.736	0.1643	-x, 1-y, z
N51	0	0.7428	0.1446	-x, 1-y, z
C52	0	0.7561	0.1442	-x, 1-y, z
C53	0	0.7631	0.1648	-x, 1-y, z
C54	0	0.7563	0.1851	-x, 1-y, z
C55	0	0.7767	0.1647	-x, 1-y, z
C56	0	0.7835	0.1443	-x, 1-y, z
C57	0	0.7767	0.1239	-x, 1-y, z
C58	0	0.7629	0.1237	-x, 1-y, z
N59	0	0.7565	0.1036	-x, 1-y, z
C60	0	0.763	0.0836	-x, 1-y, z
C61	0	0.7766	0.083	-x, 1-y, z
C62	0	0.7834	0.1035	-x, 1-y, z
C3	0	0.7838	0.0613	-x, 1-y, z
C7	0	0.7974	0.0201	-x, 1-y, z

C11	0	0.7974	0.061	-x, 1-y, z
C15	0	0.7838	0.0202	-x, 1-y, z
C23	0	0.8042	0.0405	-x, 1-y, z
C4	0	0.7353	0.2067	-x, 1-y, z
C12	0	0.742	0.2274	-x, 1-y, z
C20	0	0.7216	0.2069	-x, 1-y, z
N43	0	0.7991	-0.0205	-x, 1-y, z
C44	0	0.8049	-0.001	-x, 1-y, z
C16	0	0.777	0.0406	-x, 1-y, z
C5	0	0.6811	0.3325	-x, 1-y, z
C6	0	0.6737	0.354	-x, 1-y, z
C13	0	0.6948	0.3327	-x, 1-y, z
C21	0	0.6746	0.3117	-x, 1-y, z
C24	0	0.6952	0.2917	-x, 1-y, z
C25	0	0.7216	0.2478	-x, 1-y, z
C26	0	0.6816	0.2915	-x, 1-y, z
C27	0	0.7148	0.2273	-x, 1-y, z
N45	0	0.7021	0.2707	-x, 1-y, z
C46	0	0.7147	0.2695	-x, 1-y, z
C19	0	0.7352	0.2478	-x, 1-y, z
C1	0	0.8266	-0.1048	-x, 1-y, z
C2	0	0.8195	-0.083	-x, 1-y, z
C8	0	0.8062	-0.0414	-x, 1-y, z
C9	0	0.8197	-0.1252	-x, 1-y, z
C10	0	0.8265	-0.0625	-x, 1-y, z
C14	0	0.7992	-0.0617	-x, 1-y, z
C22	0	0.8198	-0.0419	-x, 1-y, z
C28	0	0.653	0.3741	-x, 1-y, z
C29	0	0.6385	0.3738	-x, 1-y, z
C32	0	0.6111	0.3733	-x, 1-y, z
C33	0	0.6601	0.3539	-x, 1-y, z
C34	0	0.6314	0.3941	-x, 1-y, z
C36	0	0.6181	0.3529	-x, 1-y, z
C38	0	0.6318	0.3532	-x, 1-y, z
C40	0	0.6178	0.3938	-x, 1-y, z
C18	0	0.7018	0.3125	-x, 1-y, z
C17	0	0.8059	-0.0823	-x, 1-y, z
C42	0	0.8402	-0.1054	-x, 1-y, z
N47	0	0.5971	0.3736	-x, 1-y, z
C48	0	0.5901	0.3553	-x, 1-y, z
C30	0	0.5485	0.3571	-x, 1-y, z
C31	0	0.5759	0.3563	-x, 1-y, z
C35	0	0.5556	0.3773	-x, 1-y, z
C37	0	0.5688	0.3361	-x, 1-y, z
C39	0	0.5552	0.3365	-x, 1-y, z

C41	0	0.5692	0.3769	-x, 1-y, z
C63	0	0.534	0.3575	-x, 1-y, z
C64	0	0.5269	0.3374	-x, 1-y, z
N65	0	0.5136	0.3377	-x, 1-y, z
C66	0	0.5068	0.3575	-x, 1-y, z
C67	0	0.5136	0.3782	-x, 1-y, z
C68	0	0.5272	0.378	-x, 1-y, z
C69	0	0.5068	0.3986	-x, 1-y, z
C6	0	0.8263	-0.146	x, 1/2+y, -1/2+z
C6	0	0.1737	-0.146	x, 1/2+y, -1/2+z
C9	0	0.6803	0.3748	x, 1/2+y, 1/2+z
C9	0	0.3197	0.3748	-x, 1/2-y, 1/2+z
C28	0	0.847	-0.1259	x, 1/2+y, -1/2+z
C28	0	0.153	-0.1259	-x, 1/2-y, -1/2+z
C42	0	0.6598	0.3946	x, 1/2+y, 1/2+z
C42	0	0.3402	0.3946	-x, 1/2-y, 1/2+z
C1	0	-0.3266	0.3952	x, -1/2+y, 1/2+z
C2	0	-0.3195	0.417	x, -1/2+y, 1/2+z
C3	0	-0.2838	0.5613	x, -1/2+y, 1/2+z
C4	0	-0.2353	0.7067	x, -1/2+y, 1/2+z
C5	0	-0.1811	0.8325	x, -1/2+y, 1/2+z
C6	0	-0.1737	0.854	x, -1/2+y, 1/2+z
C7	0	-0.2974	0.5201	x, -1/2+y, 1/2+z
C8	0	-0.3062	0.4586	x, -1/2+y, 1/2+z
C9	0	-0.3197	0.3748	x, -1/2+y, 1/2+z
C10	0	-0.3265	0.4375	x, -1/2+y, 1/2+z
C11	0	-0.2974	0.561	x, -1/2+y, 1/2+z
C12	0	-0.242	0.7274	x, -1/2+y, 1/2+z
C13	0	-0.1948	0.8327	x, -1/2+y, 1/2+z
C14	0	-0.2992	0.4383	x, -1/2+y, 1/2+z
C15	0	-0.2838	0.5202	x, -1/2+y, 1/2+z
C16	0	-0.277	0.5406	x, -1/2+y, 1/2+z
C17	0	-0.3059	0.4177	x, -1/2+y, 1/2+z
C18	0	-0.2018	0.8125	x, -1/2+y, 1/2+z
C19	0	-0.2352	0.7478	x, -1/2+y, 1/2+z
C20	0	-0.2216	0.7069	x, -1/2+y, 1/2+z
C21	0	-0.1746	0.8117	x, -1/2+y, 1/2+z
C22	0	-0.3199	0.4581	x, -1/2+y, 1/2+z
C23	0	-0.3042	0.5405	x, -1/2+y, 1/2+z
C24	0	-0.1952	0.7917	x, -1/2+y, 1/2+z
C25	0	-0.2216	0.7478	x, -1/2+y, 1/2+z
C26	0	-0.1816	0.7915	x, -1/2+y, 1/2+z
C27	0	-0.2148	0.7273	x, -1/2+y, 1/2+z
C28	0	-0.153	0.8741	x, -1/2+y, 1/2+z
C29	0	-0.1386	0.8738	x, -1/2+y, 1/2+z

C30	0	-0.0485	0.8571	x, -1/2+y, 1/2+z
C31	0	-0.0759	0.8563	x, -1/2+y, 1/2+z
C32	0	-0.1111	0.8733	x, -1/2+y, 1/2+z
C33	0	-0.1601	0.8539	x, -1/2+y, 1/2+z
C34	0	-0.1314	0.8941	x, -1/2+y, 1/2+z
C35	0	-0.0556	0.8773	x, -1/2+y, 1/2+z
C36	0	-0.1181	0.8529	x, -1/2+y, 1/2+z
C37	0	-0.0688	0.8361	x, -1/2+y, 1/2+z
C38	0	-0.1317	0.8532	x, -1/2+y, 1/2+z
C39	0	-0.0552	0.8365	x, -1/2+y, 1/2+z
C40	0	-0.1178	0.8938	x, -1/2+y, 1/2+z
C41	0	-0.0692	0.8769	x, -1/2+y, 1/2+z
C42	0	-0.3402	0.3946	x, -1/2+y, 1/2+z
N43	0	-0.2991	0.4795	x, -1/2+y, 1/2+z
C44	0	-0.3049	0.499	x, -1/2+y, 1/2+z
N45	0	-0.2021	0.7707	x, -1/2+y, 1/2+z
C46	0	-0.2147	0.7695	x, -1/2+y, 1/2+z
N47	0	-0.0971	0.8736	x, -1/2+y, 1/2+z
C48	0	-0.0902	0.8553	x, -1/2+y, 1/2+z
C49	0	-0.2426	0.6852	x, -1/2+y, 1/2+z
C50	0	-0.236	0.6643	x, -1/2+y, 1/2+z
N51	0	-0.2428	0.6446	x, -1/2+y, 1/2+z
C52	0	-0.2561	0.6442	x, -1/2+y, 1/2+z
C53	0	-0.2631	0.6648	x, -1/2+y, 1/2+z
C54	0	-0.2563	0.6851	x, -1/2+y, 1/2+z
C55	0	-0.2767	0.6647	x, -1/2+y, 1/2+z
C56	0	-0.2835	0.6443	x, -1/2+y, 1/2+z
C57	0	-0.2767	0.6239	x, -1/2+y, 1/2+z
C58	0	-0.2629	0.6237	x, -1/2+y, 1/2+z
N59	0	-0.2565	0.6036	x, -1/2+y, 1/2+z
C60	0	-0.263	0.5836	x, -1/2+y, 1/2+z
C61	0	-0.2766	0.583	x, -1/2+y, 1/2+z
C62	0	-0.2834	0.6035	x, -1/2+y, 1/2+z
C63	0	-0.034	0.8575	x, -1/2+y, 1/2+z
C64	0	-0.0269	0.8374	x, -1/2+y, 1/2+z
N65	0	-0.0136	0.8377	x, -1/2+y, 1/2+z
C66	0	-0.0068	0.8575	x, -1/2+y, 1/2+z
C67	0	-0.0136	0.8782	x, -1/2+y, 1/2+z
C68	0	-0.0272	0.878	x, -1/2+y, 1/2+z
C69	0	-0.0068	0.8986	x, -1/2+y, 1/2+z
C49	0	0.2426	0.6852	-x, 1/2-y, 1/2+z
C50	0	0.236	0.6643	-x, 1/2-y, 1/2+z
N51	0	0.2428	0.6446	-x, 1/2-y, 1/2+z
C52	0	0.2561	0.6442	-x, 1/2-y, 1/2+z
C53	0	0.2631	0.6648	-x, 1/2-y, 1/2+z

C54	0	0.2563	0.6851	-x, 1/2-y, 1/2+z
C55	0	0.2767	0.6647	-x, 1/2-y, 1/2+z
C56	0	0.2835	0.6443	-x, 1/2-y, 1/2+z
C57	0	0.2767	0.6239	-x, 1/2-y, 1/2+z
C58	0	0.2629	0.6237	-x, 1/2-y, 1/2+z
N59	0	0.2565	0.6036	-x, 1/2-y, 1/2+z
C60	0	0.263	0.5836	-x, 1/2-y, 1/2+z
C61	0	0.2766	0.583	-x, 1/2-y, 1/2+z
C62	0	0.2834	0.6035	-x, 1/2-y, 1/2+z
C3	0	0.2838	0.5613	-x, 1/2-y, 1/2+z
C7	0	0.2974	0.5201	-x, 1/2-y, 1/2+z
C11	0	0.2974	0.561	-x, 1/2-y, 1/2+z
C15	0	0.2838	0.5202	-x, 1/2-y, 1/2+z
C23	0	0.3042	0.5405	-x, 1/2-y, 1/2+z
C4	0	0.2353	0.7067	-x, 1/2-y, 1/2+z
C12	0	0.242	0.7274	-x, 1/2-y, 1/2+z
C20	0	0.2216	0.7069	-x, 1/2-y, 1/2+z
N43	0	0.2991	0.4795	-x, 1/2-y, 1/2+z
C44	0	0.3049	0.499	-x, 1/2-y, 1/2+z
C16	0	0.277	0.5406	-x, 1/2-y, 1/2+z
C5	0	0.1811	0.8325	-x, 1/2-y, 1/2+z
C6	0	0.1737	0.854	-x, 1/2-y, 1/2+z
C13	0	0.1948	0.8327	-x, 1/2-y, 1/2+z
C21	0	0.1746	0.8117	-x, 1/2-y, 1/2+z
C24	0	0.1952	0.7917	-x, 1/2-y, 1/2+z
C25	0	0.2216	0.7478	-x, 1/2-y, 1/2+z
C26	0	0.1816	0.7915	-x, 1/2-y, 1/2+z
C27	0	0.2148	0.7273	-x, 1/2-y, 1/2+z
N45	0	0.2021	0.7707	-x, 1/2-y, 1/2+z
C46	0	0.2147	0.7695	-x, 1/2-y, 1/2+z
C19	0	0.2352	0.7478	-x, 1/2-y, 1/2+z
C1	0	0.3266	0.3952	-x, 1/2-y, 1/2+z
C2	0	0.3195	0.417	-x, 1/2-y, 1/2+z
C8	0	0.3062	0.4586	-x, 1/2-y, 1/2+z
C10	0	0.3265	0.4375	-x, 1/2-y, 1/2+z
C14	0	0.2992	0.4383	-x, 1/2-y, 1/2+z
C22	0	0.3198	0.4581	-x, 1/2-y, 1/2+z
C28	0	0.153	0.8741	-x, 1/2-y, 1/2+z
C29	0	0.1385	0.8738	-x, 1/2-y, 1/2+z
C32	0	0.1111	0.8733	-x, 1/2-y, 1/2+z
C33	0	0.1601	0.8539	-x, 1/2-y, 1/2+z
C34	0	0.1314	0.8941	-x, 1/2-y, 1/2+z
C36	0	0.1181	0.8529	-x, 1/2-y, 1/2+z
C38	0	0.1318	0.8532	-x, 1/2-y, 1/2+z
C40	0	0.1178	0.8938	-x, 1/2-y, 1/2+z

C18	0	0.2018	0.8125	-x, 1/2-y, 1/2+z
C17	0	0.3059	0.4177	-x, 1/2-y, 1/2+z
N47	0	0.0971	0.8736	-x, 1/2-y, 1/2+z
C48	0	0.0901	0.8553	-x, 1/2-y, 1/2+z
C30	0	0.0485	0.8571	-x, 1/2-y, 1/2+z
C31	0	0.0759	0.8563	-x, 1/2-y, 1/2+z
C35	0	0.0556	0.8773	-x, 1/2-y, 1/2+z
C37	0	0.0688	0.8361	-x, 1/2-y, 1/2+z
C39	0	0.0552	0.8365	-x, 1/2-y, 1/2+z
C41	0	0.0692	0.8769	-x, 1/2-y, 1/2+z
C63	0	0.034	0.8575	-x, 1/2-y, 1/2+z
C64	0	0.0269	0.8374	-x, 1/2-y, 1/2+z
N65	0	0.0136	0.8377	-x, 1/2-y, 1/2+z
C66	0	0.0068	0.8575	-x, 1/2-y, 1/2+z
C67	0	0.0136	0.8782	-x, 1/2-y, 1/2+z
C68	0	0.0272	0.878	-x, 1/2-y, 1/2+z
C69	0	0.0068	0.8986	-x, 1/2-y, 1/2+z
C6	0	-0.3263	0.354	-x, -y, z
C9	0	0.1803	0.8748	x, y, 1+z
C9	0	-0.1803	0.8748	-x, -y, 1+z
C28	0	-0.347	0.3741	-x, -y, z
C42	0	0.1598	0.8946	x, y, 1+z
C42	0	-0.1598	0.8946	-x, -y, 1+z
C1	0	0.6734	0.3952	x, 1/2+y, 1/2+z
C2	0	0.6805	0.417	x, 1/2+y, 1/2+z
C3	0	0.7162	0.5613	x, 1/2+y, 1/2+z
C4	0	0.7647	0.7067	x, 1/2+y, 1/2+z
C5	0	0.8189	0.8325	x, 1/2+y, 1/2+z
C6	0	0.8263	0.854	x, 1/2+y, 1/2+z
C7	0	0.7026	0.5201	x, 1/2+y, 1/2+z
C8	0	0.6938	0.4586	x, 1/2+y, 1/2+z
C10	0	0.6735	0.4375	x, 1/2+y, 1/2+z
C11	0	0.7026	0.561	x, 1/2+y, 1/2+z
C12	0	0.758	0.7274	x, 1/2+y, 1/2+z
C13	0	0.8052	0.8327	x, 1/2+y, 1/2+z
C14	0	0.7008	0.4383	x, 1/2+y, 1/2+z
C15	0	0.7162	0.5202	x, 1/2+y, 1/2+z
C16	0	0.723	0.5406	x, 1/2+y, 1/2+z
C17	0	0.6941	0.4177	x, 1/2+y, 1/2+z
C18	0	0.7982	0.8125	x, 1/2+y, 1/2+z
C19	0	0.7648	0.7478	x, 1/2+y, 1/2+z
C20	0	0.7784	0.7069	x, 1/2+y, 1/2+z
C21	0	0.8254	0.8117	x, 1/2+y, 1/2+z
C22	0	0.6802	0.4581	x, 1/2+y, 1/2+z
C23	0	0.6958	0.5405	x, 1/2+y, 1/2+z

C24	0	0.8048	0.7917	x, 1/2+y, 1/2+z
C25	0	0.7784	0.7478	x, 1/2+y, 1/2+z
C26	0	0.8184	0.7915	x, 1/2+y, 1/2+z
C27	0	0.7852	0.7273	x, 1/2+y, 1/2+z
C28	0	0.847	0.8741	x, 1/2+y, 1/2+z
C29	0	0.8615	0.8738	x, 1/2+y, 1/2+z
C30	0	0.9515	0.8571	x, 1/2+y, 1/2+z
C31	0	0.9241	0.8563	x, 1/2+y, 1/2+z
C32	0	0.8889	0.8733	x, 1/2+y, 1/2+z
C33	0	0.8399	0.8539	x, 1/2+y, 1/2+z
C34	0	0.8686	0.8941	x, 1/2+y, 1/2+z
C35	0	0.9444	0.8773	x, 1/2+y, 1/2+z
C36	0	0.8819	0.8529	x, 1/2+y, 1/2+z
C37	0	0.9312	0.8361	x, 1/2+y, 1/2+z
C38	0	0.8682	0.8532	x, 1/2+y, 1/2+z
C39	0	0.9448	0.8365	x, 1/2+y, 1/2+z
C40	0	0.8822	0.8938	x, 1/2+y, 1/2+z
C41	0	0.9308	0.8769	x, 1/2+y, 1/2+z
N43	0	0.7009	0.4795	x, 1/2+y, 1/2+z
C44	0	0.6951	0.499	x, 1/2+y, 1/2+z
N45	0	0.7979	0.7707	x, 1/2+y, 1/2+z
C46	0	0.7853	0.7695	x, 1/2+y, 1/2+z
N47	0	0.9029	0.8736	x, 1/2+y, 1/2+z
C48	0	0.9099	0.8553	x, 1/2+y, 1/2+z
C49	0	0.7574	0.6852	x, 1/2+y, 1/2+z
C50	0	0.764	0.6643	x, 1/2+y, 1/2+z
N51	0	0.7572	0.6446	x, 1/2+y, 1/2+z
C52	0	0.7439	0.6442	x, 1/2+y, 1/2+z
C53	0	0.7369	0.6648	x, 1/2+y, 1/2+z
C54	0	0.7437	0.6851	x, 1/2+y, 1/2+z
C55	0	0.7233	0.6647	x, 1/2+y, 1/2+z
C56	0	0.7165	0.6443	x, 1/2+y, 1/2+z
C57	0	0.7233	0.6239	x, 1/2+y, 1/2+z
C58	0	0.7371	0.6237	x, 1/2+y, 1/2+z
N59	0	0.7435	0.6036	x, 1/2+y, 1/2+z
C60	0	0.737	0.5836	x, 1/2+y, 1/2+z
C61	0	0.7234	0.583	x, 1/2+y, 1/2+z
C62	0	0.7166	0.6035	x, 1/2+y, 1/2+z
C63	0	0.966	0.8575	x, 1/2+y, 1/2+z
C64	0	0.9731	0.8374	x, 1/2+y, 1/2+z
N65	0	0.9864	0.8377	x, 1/2+y, 1/2+z
C66	0	0.9932	0.8575	x, 1/2+y, 1/2+z
C67	0	0.9864	0.8782	x, 1/2+y, 1/2+z
C68	0	0.9728	0.878	x, 1/2+y, 1/2+z
C69	0	0.9932	0.8986	x, 1/2+y, 1/2+z

C49	0	1.2426	0.6852	-x, 1.5-y, 1/2+z
C50	0	1.2361	0.6643	-x, 1.5-y, 1/2+z
N51	0	1.2428	0.6446	-x, 1.5-y, 1/2+z
C52	0	1.2561	0.6442	-x, 1.5-y, 1/2+z
C53	0	1.2631	0.6648	-x, 1.5-y, 1/2+z
C54	0	1.2563	0.6851	-x, 1.5-y, 1/2+z
C55	0	1.2767	0.6647	-x, 1.5-y, 1/2+z
C56	0	1.2835	0.6443	-x, 1.5-y, 1/2+z
C57	0	1.2767	0.6239	-x, 1.5-y, 1/2+z
C58	0	1.2629	0.6237	-x, 1.5-y, 1/2+z
N59	0	1.2565	0.6036	-x, 1.5-y, 1/2+z
C60	0	1.263	0.5836	-x, 1.5-y, 1/2+z
C61	0	1.2766	0.583	-x, 1.5-y, 1/2+z
C62	0	1.2834	0.6035	-x, 1.5-y, 1/2+z
C3	0	1.2838	0.5613	-x, 1.5-y, 1/2+z
C7	0	1.2974	0.5201	-x, 1.5-y, 1/2+z
C11	0	1.2974	0.561	-x, 1.5-y, 1/2+z
C15	0	1.2838	0.5202	-x, 1.5-y, 1/2+z
C23	0	1.3042	0.5405	-x, 1.5-y, 1/2+z
C4	0	1.2353	0.7067	-x, 1.5-y, 1/2+z
C12	0	1.242	0.7274	-x, 1.5-y, 1/2+z
C20	0	1.2216	0.7069	-x, 1.5-y, 1/2+z
N43	0	1.2991	0.4795	-x, 1.5-y, 1/2+z
C44	0	1.3049	0.499	-x, 1.5-y, 1/2+z
C16	0	1.277	0.5406	-x, 1.5-y, 1/2+z
C5	0	1.1811	0.8325	-x, 1.5-y, 1/2+z
C6	0	1.1738	0.854	-x, 1.5-y, 1/2+z
C13	0	1.1948	0.8327	-x, 1.5-y, 1/2+z
C21	0	1.1746	0.8117	-x, 1.5-y, 1/2+z
C24	0	1.1952	0.7917	-x, 1.5-y, 1/2+z
C25	0	1.2216	0.7478	-x, 1.5-y, 1/2+z
C26	0	1.1816	0.7915	-x, 1.5-y, 1/2+z
C27	0	1.2148	0.7273	-x, 1.5-y, 1/2+z
N45	0	1.2021	0.7707	-x, 1.5-y, 1/2+z
C46	0	1.2147	0.7695	-x, 1.5-y, 1/2+z
C19	0	1.2352	0.7478	-x, 1.5-y, 1/2+z
C1	0	1.3266	0.3952	-x, 1.5-y, 1/2+z
C2	0	1.3195	0.417	-x, 1.5-y, 1/2+z
C8	0	1.3062	0.4586	-x, 1.5-y, 1/2+z
C9	0	1.3197	0.3748	-x, 1.5-y, 1/2+z
C10	0	1.3264	0.4375	-x, 1.5-y, 1/2+z
C14	0	1.2992	0.4383	-x, 1.5-y, 1/2+z
C22	0	1.3198	0.4581	-x, 1.5-y, 1/2+z
C28	0	1.153	0.8741	-x, 1.5-y, 1/2+z
C29	0	1.1385	0.8738	-x, 1.5-y, 1/2+z

C32	0	1.1111	0.8733	-x, 1.5-y, 1/2+z
C33	0	1.1601	0.8539	-x, 1.5-y, 1/2+z
C34	0	1.1314	0.8941	-x, 1.5-y, 1/2+z
C36	0	1.1181	0.8529	-x, 1.5-y, 1/2+z
C38	0	1.1318	0.8532	-x, 1.5-y, 1/2+z
C40	0	1.1178	0.8938	-x, 1.5-y, 1/2+z
C18	0	1.2018	0.8125	-x, 1.5-y, 1/2+z
C17	0	1.3059	0.4177	-x, 1.5-y, 1/2+z
C42	0	1.3402	0.3946	-x, 1.5-y, 1/2+z
N47	0	1.0971	0.8736	-x, 1.5-y, 1/2+z
C48	0	1.0901	0.8553	-x, 1.5-y, 1/2+z
C30	0	1.0485	0.8571	-x, 1.5-y, 1/2+z
C31	0	1.0759	0.8563	-x, 1.5-y, 1/2+z
C35	0	1.0556	0.8773	-x, 1.5-y, 1/2+z
C37	0	1.0688	0.8361	-x, 1.5-y, 1/2+z
C39	0	1.0552	0.8365	-x, 1.5-y, 1/2+z
C41	0	1.0692	0.8769	-x, 1.5-y, 1/2+z
C63	0	1.034	0.8575	-x, 1.5-y, 1/2+z
C64	0	1.0269	0.8374	-x, 1.5-y, 1/2+z
N65	0	1.0136	0.8377	-x, 1.5-y, 1/2+z
C66	0	1.0068	0.8575	-x, 1.5-y, 1/2+z
C67	0	1.0136	0.8782	-x, 1.5-y, 1/2+z
C68	0	1.0272	0.878	-x, 1.5-y, 1/2+z
C69	0	1.0068	0.8986	-x, 1.5-y, 1/2+z
C6	0	1.3262	0.354	x, 1+y, z
C9	0	1.1803	0.8748	x, 1+y, 1+z
C9	0	0.8197	0.8748	-x, 1-y, 1+z
C28	0	1.347	0.3741	x, 1+y, z
C42	0	1.1598	0.8946	x, 1+y, 1+z
C42	0	0.8402	0.8946	-x, 1-y, 1+z
C1	1	0.1734	-0.1048	1+x, y, z
C2	1	0.1805	-0.083	1+x, y, z
C3	1	0.2162	0.0613	1+x, y, z
C4	1	0.2647	0.2067	1+x, y, z
C5	1	0.3189	0.3325	1+x, y, z
C6	1	0.3263	0.354	1+x, y, z
C7	1	0.2026	0.0201	1+x, y, z
C8	1	0.1938	-0.0414	1+x, y, z
C9	1	0.1803	-0.1252	1+x, y, z
C10	1	0.1735	-0.0625	1+x, y, z
C11	1	0.2026	0.061	1+x, y, z
C12	1	0.258	0.2274	1+x, y, z
C13	1	0.3052	0.3327	1+x, y, z
C14	1	0.2008	-0.0617	1+x, y, z
C15	1	0.2162	0.0202	1+x, y, z

C16	1	0.223	0.0406	1+x, y, z
C17	1	0.1941	-0.0823	1+x, y, z
C18	1	0.2982	0.3125	1+x, y, z
C19	1	0.2648	0.2478	1+x, y, z
C20	1	0.2784	0.2069	1+x, y, z
C21	1	0.3254	0.3117	1+x, y, z
C22	1	0.1801	-0.0419	1+x, y, z
C23	1	0.1958	0.0405	1+x, y, z
C24	1	0.3048	0.2917	1+x, y, z
C25	1	0.2784	0.2478	1+x, y, z
C26	1	0.3184	0.2915	1+x, y, z
C27	1	0.2852	0.2273	1+x, y, z
C28	1	0.347	0.3741	1+x, y, z
C29	1	0.3614	0.3738	1+x, y, z
C30	1	0.4515	0.3571	1+x, y, z
C31	1	0.4241	0.3563	1+x, y, z
C32	1	0.3889	0.3733	1+x, y, z
C33	1	0.3399	0.3539	1+x, y, z
C34	1	0.3686	0.3941	1+x, y, z
C35	1	0.4444	0.3773	1+x, y, z
C36	1	0.3819	0.3529	1+x, y, z
C37	1	0.4312	0.3361	1+x, y, z
C38	1	0.3683	0.3532	1+x, y, z
C39	1	0.4448	0.3365	1+x, y, z
C40	1	0.3822	0.3938	1+x, y, z
C41	1	0.4308	0.3769	1+x, y, z
C42	1	0.1598	-0.1054	1+x, y, z
N43	1	0.2009	-0.0205	1+x, y, z
C44	1	0.1951	-0.001	1+x, y, z
N45	1	0.2979	0.2707	1+x, y, z
C46	1	0.2853	0.2695	1+x, y, z
N47	1	0.4029	0.3736	1+x, y, z
C48	1	0.4098	0.3553	1+x, y, z
C49	1	0.2574	0.1852	1+x, y, z
C50	1	0.264	0.1643	1+x, y, z
N51	1	0.2572	0.1446	1+x, y, z
C52	1	0.2439	0.1442	1+x, y, z
C53	1	0.2369	0.1648	1+x, y, z
C54	1	0.2437	0.1851	1+x, y, z
C55	1	0.2233	0.1647	1+x, y, z
C56	1	0.2165	0.1443	1+x, y, z
C57	1	0.2233	0.1239	1+x, y, z
C58	1	0.2371	0.1237	1+x, y, z
N59	1	0.2435	0.1036	1+x, y, z
C60	1	0.2371	0.0836	1+x, y, z

C61	1	0.2234	0.083	1+x, y, z
C62	1	0.2166	0.1035	1+x, y, z
C63	1	0.466	0.3575	1+x, y, z
C64	1	0.4731	0.3374	1+x, y, z
N65	1	0.4864	0.3377	1+x, y, z
C66	1	0.4932	0.3575	1+x, y, z
C67	1	0.4864	0.3782	1+x, y, z
C68	1	0.4728	0.378	1+x, y, z
C69	1	0.4932	0.3986	1+x, y, z
C49	1	0.7426	0.1852	1-x, 1-y, z
C50	1	0.736	0.1643	1-x, 1-y, z
N51	1	0.7428	0.1446	1-x, 1-y, z
C52	1	0.7561	0.1442	1-x, 1-y, z
C53	1	0.7631	0.1648	1-x, 1-y, z
C54	1	0.7563	0.1851	1-x, 1-y, z
C55	1	0.7767	0.1647	1-x, 1-y, z
C56	1	0.7835	0.1443	1-x, 1-y, z
C57	1	0.7767	0.1239	1-x, 1-y, z
C58	1	0.7629	0.1237	1-x, 1-y, z
N59	1	0.7565	0.1036	1-x, 1-y, z
C60	1	0.763	0.0836	1-x, 1-y, z
C61	1	0.7766	0.083	1-x, 1-y, z
C62	1	0.7834	0.1035	1-x, 1-y, z
C3	1	0.7838	0.0613	1-x, 1-y, z
C7	1	0.7974	0.0201	1-x, 1-y, z
C11	1	0.7974	0.061	1-x, 1-y, z
C15	1	0.7838	0.0202	1-x, 1-y, z
C23	1	0.8042	0.0405	1-x, 1-y, z
C4	1	0.7353	0.2067	1-x, 1-y, z
C12	1	0.742	0.2274	1-x, 1-y, z
C20	1	0.7216	0.2069	1-x, 1-y, z
N43	1	0.7991	-0.0205	1-x, 1-y, z
C44	1	0.8049	-0.001	1-x, 1-y, z
C16	1	0.777	0.0406	1-x, 1-y, z
C5	1	0.6811	0.3325	1-x, 1-y, z
C6	1	0.6737	0.354	1-x, 1-y, z
C13	1	0.6948	0.3327	1-x, 1-y, z
C21	1	0.6746	0.3117	1-x, 1-y, z
C24	1	0.6952	0.2917	1-x, 1-y, z
C25	1	0.7216	0.2478	1-x, 1-y, z
C26	1	0.6816	0.2915	1-x, 1-y, z
C27	1	0.7148	0.2273	1-x, 1-y, z
N45	1	0.7021	0.2707	1-x, 1-y, z
C46	1	0.7147	0.2695	1-x, 1-y, z
C19	1	0.7352	0.2478	1-x, 1-y, z

C1	1	0.8266	-0.1048	1-x, 1-y, z
C2	1	0.8195	-0.083	1-x, 1-y, z
C8	1	0.8062	-0.0414	1-x, 1-y, z
C9	1	0.8197	-0.1252	1-x, 1-y, z
C10	1	0.8265	-0.0625	1-x, 1-y, z
C14	1	0.7992	-0.0617	1-x, 1-y, z
C22	1	0.8198	-0.0419	1-x, 1-y, z
C28	1	0.653	0.3741	1-x, 1-y, z
C29	1	0.6385	0.3738	1-x, 1-y, z
C32	1	0.6111	0.3733	1-x, 1-y, z
C33	1	0.6601	0.3539	1-x, 1-y, z
C34	1	0.6314	0.3941	1-x, 1-y, z
C36	1	0.6181	0.3529	1-x, 1-y, z
C38	1	0.6318	0.3532	1-x, 1-y, z
C40	1	0.6178	0.3938	1-x, 1-y, z
C18	1	0.7018	0.3125	1-x, 1-y, z
C17	1	0.8059	-0.0823	1-x, 1-y, z
C42	1	0.8402	-0.1054	1-x, 1-y, z
N47	1	0.5971	0.3736	1-x, 1-y, z
C48	1	0.5901	0.3553	1-x, 1-y, z
C30	1	0.5485	0.3571	1-x, 1-y, z
C31	1	0.5759	0.3563	1-x, 1-y, z
C35	1	0.5556	0.3773	1-x, 1-y, z
C37	1	0.5688	0.3361	1-x, 1-y, z
C39	1	0.5552	0.3365	1-x, 1-y, z
C41	1	0.5692	0.3769	1-x, 1-y, z
C63	1	0.534	0.3575	1-x, 1-y, z
C64	1	0.5269	0.3374	1-x, 1-y, z
N65	1	0.5136	0.3377	1-x, 1-y, z
C66	1	0.5068	0.3575	1-x, 1-y, z
C67	1	0.5136	0.3782	1-x, 1-y, z
C68	1	0.5272	0.378	1-x, 1-y, z
C69	1	0.5068	0.3986	1-x, 1-y, z
C6	1	0.8263	-0.146	1+x, 1/2+y, -1/2+z
C6	1	0.1737	-0.146	1-x, 1/2-y, -1/2+z
C9	1	0.6803	0.3748	1+x, 1/2+y, 1/2+z
C9	1	0.3197	0.3748	1-x, 1/2-y, 1/2+z
C28	1	0.847	-0.1259	1+x, 1/2+y, -1/2+z
C28	1	0.153	-0.1259	1-x, 1/2-y, -1/2+z
C42	1	0.6598	0.3946	1+x, 1/2+y, 1/2+z
C42	1	0.3402	0.3946	1-x, 1/2-y, 1/2+z
C1	1	-0.3266	0.3952	1+x, -1/2+y, 1/2+z
C2	1	-0.3195	0.417	1+x, -1/2+y, 1/2+z
C3	1	-0.2838	0.5613	1+x, -1/2+y, 1/2+z
C4	1	-0.2353	0.7067	1+x, -1/2+y, 1/2+z

C5	1	-0.1811	0.8325	1+x, -1/2+y, 1/2+z
C6	1	-0.1737	0.854	1+x, -1/2+y, 1/2+z
C7	1	-0.2974	0.5201	1+x, -1/2+y, 1/2+z
C8	1	-0.3062	0.4586	1+x, -1/2+y, 1/2+z
C9	1	-0.3197	0.3748	1+x, -1/2+y, 1/2+z
C10	1	-0.3265	0.4375	1+x, -1/2+y, 1/2+z
C11	1	-0.2974	0.561	1+x, -1/2+y, 1/2+z
C12	1	-0.242	0.7274	1+x, -1/2+y, 1/2+z
C13	1	-0.1948	0.8327	1+x, -1/2+y, 1/2+z
C14	1	-0.2992	0.4383	1+x, -1/2+y, 1/2+z
C15	1	-0.2838	0.5202	1+x, -1/2+y, 1/2+z
C16	1	-0.277	0.5406	1+x, -1/2+y, 1/2+z
C17	1	-0.3059	0.4177	1+x, -1/2+y, 1/2+z
C18	1	-0.2018	0.8125	1+x, -1/2+y, 1/2+z
C19	1	-0.2352	0.7478	1+x, -1/2+y, 1/2+z
C20	1	-0.2216	0.7069	1+x, -1/2+y, 1/2+z
C21	1	-0.1746	0.8117	1+x, -1/2+y, 1/2+z
C22	1	-0.3199	0.4581	1+x, -1/2+y, 1/2+z
C23	1	-0.3042	0.5405	1+x, -1/2+y, 1/2+z
C24	1	-0.1952	0.7917	1+x, -1/2+y, 1/2+z
C25	1	-0.2216	0.7478	1+x, -1/2+y, 1/2+z
C26	1	-0.1816	0.7915	1+x, -1/2+y, 1/2+z
C27	1	-0.2148	0.7273	1+x, -1/2+y, 1/2+z
C28	1	-0.153	0.8741	1+x, -1/2+y, 1/2+z
C29	1	-0.1386	0.8738	1+x, -1/2+y, 1/2+z
C30	1	-0.0485	0.8571	1+x, -1/2+y, 1/2+z
C31	1	-0.0759	0.8563	1+x, -1/2+y, 1/2+z
C32	1	-0.1111	0.8733	1+x, -1/2+y, 1/2+z
C33	1	-0.1601	0.8539	1+x, -1/2+y, 1/2+z
C34	1	-0.1314	0.8941	1+x, -1/2+y, 1/2+z
C35	1	-0.0556	0.8773	1+x, -1/2+y, 1/2+z
C36	1	-0.1181	0.8529	1+x, -1/2+y, 1/2+z
C37	1	-0.0688	0.8361	1+x, -1/2+y, 1/2+z
C38	1	-0.1317	0.8532	1+x, -1/2+y, 1/2+z
C39	1	-0.0552	0.8365	1+x, -1/2+y, 1/2+z
C40	1	-0.1178	0.8938	1+x, -1/2+y, 1/2+z
C41	1	-0.0692	0.8769	1+x, -1/2+y, 1/2+z
C42	1	-0.3402	0.3946	1+x, -1/2+y, 1/2+z
N43	1	-0.2991	0.4795	1+x, -1/2+y, 1/2+z
C44	1	-0.3049	0.499	1+x, -1/2+y, 1/2+z
N45	1	-0.2021	0.7707	1+x, -1/2+y, 1/2+z
C46	1	-0.2147	0.7695	1+x, -1/2+y, 1/2+z
N47	1	-0.0971	0.8736	1+x, -1/2+y, 1/2+z
C48	1	-0.0902	0.8553	1+x, -1/2+y, 1/2+z
C49	1	-0.2426	0.6852	1+x, -1/2+y, 1/2+z

C50	1	-0.236	0.6643	1+x, -1/2+y, 1/2+z
N51	1	-0.2428	0.6446	1+x, -1/2+y, 1/2+z
C52	1	-0.2561	0.6442	1+x, -1/2+y, 1/2+z
C53	1	-0.2631	0.6648	1+x, -1/2+y, 1/2+z
C54	1	-0.2563	0.6851	1+x, -1/2+y, 1/2+z
C55	1	-0.2767	0.6647	1+x, -1/2+y, 1/2+z
C56	1	-0.2835	0.6443	1+x, -1/2+y, 1/2+z
C57	1	-0.2767	0.6239	1+x, -1/2+y, 1/2+z
C58	1	-0.2629	0.6237	1+x, -1/2+y, 1/2+z
N59	1	-0.2565	0.6036	1+x, -1/2+y, 1/2+z
C60	1	-0.263	0.5836	1+x, -1/2+y, 1/2+z
C61	1	-0.2766	0.583	1+x, -1/2+y, 1/2+z
C62	1	-0.2834	0.6035	1+x, -1/2+y, 1/2+z
C63	1	-0.034	0.8575	1+x, -1/2+y, 1/2+z
C64	1	-0.0269	0.8374	1+x, -1/2+y, 1/2+z
N65	1	-0.0136	0.8377	1+x, -1/2+y, 1/2+z
C66	1	-0.0068	0.8575	1+x, -1/2+y, 1/2+z
C67	1	-0.0136	0.8782	1+x, -1/2+y, 1/2+z
C68	1	-0.0272	0.878	1+x, -1/2+y, 1/2+z
C69	1	-0.0068	0.8986	1+x, -1/2+y, 1/2+z
C49	1	0.2426	0.6852	1-x, 1/2-y, 1/2+z
C50	1	0.236	0.6643	1-x, 1/2-y, 1/2+z
N51	1	0.2428	0.6446	1-x, 1/2-y, 1/2+z
C52	1	0.2561	0.6442	1-x, 1/2-y, 1/2+z
C53	1	0.2631	0.6648	1-x, 1/2-y, 1/2+z
C54	1	0.2563	0.6851	1-x, 1/2-y, 1/2+z
C55	1	0.2767	0.6647	1-x, 1/2-y, 1/2+z
C56	1	0.2835	0.6443	1-x, 1/2-y, 1/2+z
C57	1	0.2767	0.6239	1-x, 1/2-y, 1/2+z
C58	1	0.2629	0.6237	1-x, 1/2-y, 1/2+z
N59	1	0.2565	0.6036	1-x, 1/2-y, 1/2+z
C60	1	0.263	0.5836	1-x, 1/2-y, 1/2+z
C61	1	0.2766	0.583	1-x, 1/2-y, 1/2+z
C62	1	0.2834	0.6035	1-x, 1/2-y, 1/2+z
C3	1	0.2838	0.5613	1-x, 1/2-y, 1/2+z
C7	1	0.2974	0.5201	1-x, 1/2-y, 1/2+z
C11	1	0.2974	0.561	1-x, 1/2-y, 1/2+z
C15	1	0.2838	0.5202	1-x, 1/2-y, 1/2+z
C23	1	0.3042	0.5405	1-x, 1/2-y, 1/2+z
C4	1	0.2353	0.7067	1-x, 1/2-y, 1/2+z
C12	1	0.242	0.7274	1-x, 1/2-y, 1/2+z
C20	1	0.2216	0.7069	1-x, 1/2-y, 1/2+z
N43	1	0.2991	0.4795	1-x, 1/2-y, 1/2+z
C44	1	0.3049	0.499	1-x, 1/2-y, 1/2+z
C16	1	0.277	0.5406	1-x, 1/2-y, 1/2+z

C5	1	0.1811	0.8325	1-x, 1/2-y, 1/2+z
C6	1	0.1737	0.854	1-x, 1/2-y, 1/2+z
C13	1	0.1948	0.8327	1-x, 1/2-y, 1/2+z
C21	1	0.1746	0.8117	1-x, 1/2-y, 1/2+z
C24	1	0.1952	0.7917	1-x, 1/2-y, 1/2+z
C25	1	0.2216	0.7478	1-x, 1/2-y, 1/2+z
C26	1	0.1816	0.7915	1-x, 1/2-y, 1/2+z
C27	1	0.2148	0.7273	1-x, 1/2-y, 1/2+z
N45	1	0.2021	0.7707	1-x, 1/2-y, 1/2+z
C46	1	0.2147	0.7695	1-x, 1/2-y, 1/2+z
C19	1	0.2352	0.7478	1-x, 1/2-y, 1/2+z
C1	1	0.3266	0.3952	1-x, 1/2-y, 1/2+z
C2	1	0.3195	0.417	1-x, 1/2-y, 1/2+z
C8	1	0.3062	0.4586	1-x, 1/2-y, 1/2+z
C10	1	0.3265	0.4375	1-x, 1/2-y, 1/2+z
C14	1	0.2992	0.4383	1-x, 1/2-y, 1/2+z
C22	1	0.3198	0.4581	1-x, 1/2-y, 1/2+z
C28	1	0.153	0.8741	1-x, 1/2-y, 1/2+z
C29	1	0.1385	0.8738	1-x, 1/2-y, 1/2+z
C32	1	0.1111	0.8733	1-x, 1/2-y, 1/2+z
C33	1	0.1601	0.8539	1-x, 1/2-y, 1/2+z
C34	1	0.1314	0.8941	1-x, 1/2-y, 1/2+z
C36	1	0.1181	0.8529	1-x, 1/2-y, 1/2+z
C38	1	0.1318	0.8532	1-x, 1/2-y, 1/2+z
C40	1	0.1178	0.8938	1-x, 1/2-y, 1/2+z
C18	1	0.2018	0.8125	1-x, 1/2-y, 1/2+z
C17	1	0.3059	0.4177	1-x, 1/2-y, 1/2+z
N47	1	0.0971	0.8736	1-x, 1/2-y, 1/2+z
C48	1	0.0901	0.8553	1-x, 1/2-y, 1/2+z
C30	1	0.0485	0.8571	1-x, 1/2-y, 1/2+z
C31	1	0.0759	0.8563	1-x, 1/2-y, 1/2+z
C35	1	0.0556	0.8773	1-x, 1/2-y, 1/2+z
C37	1	0.0688	0.8361	1-x, 1/2-y, 1/2+z
C39	1	0.0552	0.8365	1-x, 1/2-y, 1/2+z
C41	1	0.0692	0.8769	1-x, 1/2-y, 1/2+z
C63	1	0.034	0.8575	1-x, 1/2-y, 1/2+z
C64	1	0.0269	0.8374	1-x, 1/2-y, 1/2+z
N65	1	0.0136	0.8377	1-x, 1/2-y, 1/2+z
C66	1	0.0068	0.8575	1-x, 1/2-y, 1/2+z
C67	1	0.0136	0.8782	1-x, 1/2-y, 1/2+z
C68	1	0.0272	0.878	1-x, 1/2-y, 1/2+z
C69	1	0.0068	0.8986	1-x, 1/2-y, 1/2+z
C6	1	-0.3263	0.354	1-x, -y, z
C9	1	0.1803	0.8748	1+x, y, 1+z
C9	1	-0.1803	0.8748	1-x, -y, 1+z

C28	1	-0.347	0.3741	1-x, -y, z
C42	1	0.1598	0.8946	1+x, y, 1+z
C42	1	-0.1598	0.8946	1-x, -y, 1+z
C1	1	0.6734	0.3952	1+x, 1/2+y, 1/2+z
C2	1	0.6805	0.417	1+x, 1/2+y, 1/2+z
C3	1	0.7162	0.5613	1+x, 1/2+y, 1/2+z
C4	1	0.7647	0.7067	1+x, 1/2+y, 1/2+z
C5	1	0.8189	0.8325	1+x, 1/2+y, 1/2+z
C6	1	0.8263	0.854	1+x, 1/2+y, 1/2+z
C7	1	0.7026	0.5201	1+x, 1/2+y, 1/2+z
C8	1	0.6938	0.4586	1+x, 1/2+y, 1/2+z
C10	1	0.6735	0.4375	1+x, 1/2+y, 1/2+z
C11	1	0.7026	0.561	1+x, 1/2+y, 1/2+z
C12	1	0.758	0.7274	1+x, 1/2+y, 1/2+z
C13	1	0.8052	0.8327	1+x, 1/2+y, 1/2+z
C14	1	0.7008	0.4383	1+x, 1/2+y, 1/2+z
C15	1	0.7162	0.5202	1+x, 1/2+y, 1/2+z
C16	1	0.723	0.5406	1+x, 1/2+y, 1/2+z
C17	1	0.6941	0.4177	1+x, 1/2+y, 1/2+z
C18	1	0.7982	0.8125	1+x, 1/2+y, 1/2+z
C19	1	0.7648	0.7478	1+x, 1/2+y, 1/2+z
C20	1	0.7784	0.7069	1+x, 1/2+y, 1/2+z
C21	1	0.8254	0.8117	1+x, 1/2+y, 1/2+z
C22	1	0.6802	0.4581	1+x, 1/2+y, 1/2+z
C23	1	0.6958	0.5405	1+x, 1/2+y, 1/2+z
C24	1	0.8048	0.7917	1+x, 1/2+y, 1/2+z
C25	1	0.7784	0.7478	1+x, 1/2+y, 1/2+z
C26	1	0.8184	0.7915	1+x, 1/2+y, 1/2+z
C27	1	0.7852	0.7273	1+x, 1/2+y, 1/2+z
C28	1	0.847	0.8741	1+x, 1/2+y, 1/2+z
C29	1	0.8615	0.8738	1+x, 1/2+y, 1/2+z
C30	1	0.9515	0.8571	1+x, 1/2+y, 1/2+z
C31	1	0.9241	0.8563	1+x, 1/2+y, 1/2+z
C32	1	0.8889	0.8733	1+x, 1/2+y, 1/2+z
C33	1	0.8399	0.8539	1+x, 1/2+y, 1/2+z
C34	1	0.8686	0.8941	1+x, 1/2+y, 1/2+z
C35	1	0.9444	0.8773	1+x, 1/2+y, 1/2+z
C36	1	0.8819	0.8529	1+x, 1/2+y, 1/2+z
C37	1	0.9312	0.8361	1+x, 1/2+y, 1/2+z
C38	1	0.8682	0.8532	1+x, 1/2+y, 1/2+z
C39	1	0.9448	0.8365	1+x, 1/2+y, 1/2+z
C40	1	0.8822	0.8938	1+x, 1/2+y, 1/2+z
C41	1	0.9308	0.8769	1+x, 1/2+y, 1/2+z
N43	1	0.7009	0.4795	1+x, 1/2+y, 1/2+z
C44	1	0.6951	0.499	1+x, 1/2+y, 1/2+z

N45	1	0.7979	0.7707	1+x, 1/2+y, 1/2+z
C46	1	0.7853	0.7695	1+x, 1/2+y, 1/2+z
N47	1	0.9029	0.8736	1+x, 1/2+y, 1/2+z
C48	1	0.9099	0.8553	1+x, 1/2+y, 1/2+z
C49	1	0.7574	0.6852	1+x, 1/2+y, 1/2+z
C50	1	0.764	0.6643	1+x, 1/2+y, 1/2+z
N51	1	0.7572	0.6446	1+x, 1/2+y, 1/2+z
C52	1	0.7439	0.6442	1+x, 1/2+y, 1/2+z
C53	1	0.7369	0.6648	1+x, 1/2+y, 1/2+z
C54	1	0.7437	0.6851	1+x, 1/2+y, 1/2+z
C55	1	0.7233	0.6647	1+x, 1/2+y, 1/2+z
C56	1	0.7165	0.6443	1+x, 1/2+y, 1/2+z
C57	1	0.7233	0.6239	1+x, 1/2+y, 1/2+z
C58	1	0.7371	0.6237	1+x, 1/2+y, 1/2+z
N59	1	0.7435	0.6036	1+x, 1/2+y, 1/2+z
C60	1	0.737	0.5836	1+x, 1/2+y, 1/2+z
C61	1	0.7234	0.583	1+x, 1/2+y, 1/2+z
C62	1	0.7166	0.6035	1+x, 1/2+y, 1/2+z
C63	1	0.966	0.8575	1+x, 1/2+y, 1/2+z
C64	1	0.9731	0.8374	1+x, 1/2+y, 1/2+z
N65	1	0.9864	0.8377	1+x, 1/2+y, 1/2+z
C66	1	0.9932	0.8575	1+x, 1/2+y, 1/2+z
C67	1	0.9864	0.8782	1+x, 1/2+y, 1/2+z
C68	1	0.9728	0.878	1+x, 1/2+y, 1/2+z
C69	1	0.9932	0.8986	1+x, 1/2+y, 1/2+z
C49	1	1.2426	0.6852	1-x, 1.5-y, 1/2+z
C50	1	1.2361	0.6643	1-x, 1.5-y, 1/2+z
N51	1	1.2428	0.6446	1-x, 1.5-y, 1/2+z
C52	1	1.2561	0.6442	1-x, 1.5-y, 1/2+z
C53	1	1.2631	0.6648	1-x, 1.5-y, 1/2+z
C54	1	1.2563	0.6851	1-x, 1.5-y, 1/2+z
C55	1	1.2767	0.6647	1-x, 1.5-y, 1/2+z
C56	1	1.2835	0.6443	1-x, 1.5-y, 1/2+z
C57	1	1.2767	0.6239	1-x, 1.5-y, 1/2+z
C58	1	1.2629	0.6237	1-x, 1.5-y, 1/2+z
N59	1	1.2565	0.6036	1-x, 1.5-y, 1/2+z
C60	1	1.263	0.5836	1-x, 1.5-y, 1/2+z
C61	1	1.2766	0.583	1-x, 1.5-y, 1/2+z
C62	1	1.2834	0.6035	1-x, 1.5-y, 1/2+z
C3	1	1.2838	0.5613	1-x, 1.5-y, 1/2+z
C7	1	1.2974	0.5201	1-x, 1.5-y, 1/2+z
C11	1	1.2974	0.561	1-x, 1.5-y, 1/2+z
C15	1	1.2838	0.5202	1-x, 1.5-y, 1/2+z
C23	1	1.3042	0.5405	1-x, 1.5-y, 1/2+z
C4	1	1.2353	0.7067	1-x, 1.5-y, 1/2+z

C12	1	1.242	0.7274	1-x, 1.5-y, 1/2+z
C20	1	1.2216	0.7069	1-x, 1.5-y, 1/2+z
N43	1	1.2991	0.4795	1-x, 1.5-y, 1/2+z
C44	1	1.3049	0.499	1-x, 1.5-y, 1/2+z
C16	1	1.277	0.5406	1-x, 1.5-y, 1/2+z
C5	1	1.1811	0.8325	1-x, 1.5-y, 1/2+z
C6	1	1.1738	0.854	1-x, 1.5-y, 1/2+z
C13	1	1.1948	0.8327	1-x, 1.5-y, 1/2+z
C21	1	1.1746	0.8117	1-x, 1.5-y, 1/2+z
C24	1	1.1952	0.7917	1-x, 1.5-y, 1/2+z
C25	1	1.2216	0.7478	1-x, 1.5-y, 1/2+z
C26	1	1.1816	0.7915	1-x, 1.5-y, 1/2+z
C27	1	1.2148	0.7273	1-x, 1.5-y, 1/2+z
N45	1	1.2021	0.7707	1-x, 1.5-y, 1/2+z
C46	1	1.2147	0.7695	1-x, 1.5-y, 1/2+z
C19	1	1.2352	0.7478	1-x, 1.5-y, 1/2+z
C1	1	1.3266	0.3952	1-x, 1.5-y, 1/2+z
C2	1	1.3195	0.417	1-x, 1.5-y, 1/2+z
C8	1	1.3062	0.4586	1-x, 1.5-y, 1/2+z
C9	1	1.3197	0.3748	1-x, 1.5-y, 1/2+z
C10	1	1.3264	0.4375	1-x, 1.5-y, 1/2+z
C14	1	1.2992	0.4383	1-x, 1.5-y, 1/2+z
C22	1	1.3198	0.4581	1-x, 1.5-y, 1/2+z
C28	1	1.153	0.8741	1-x, 1.5-y, 1/2+z
C29	1	1.1385	0.8738	1-x, 1.5-y, 1/2+z
C32	1	1.1111	0.8733	1-x, 1.5-y, 1/2+z
C33	1	1.1601	0.8539	1-x, 1.5-y, 1/2+z
C34	1	1.1314	0.8941	1-x, 1.5-y, 1/2+z
C36	1	1.1181	0.8529	1-x, 1.5-y, 1/2+z
C38	1	1.1318	0.8532	1-x, 1.5-y, 1/2+z
C40	1	1.1178	0.8938	1-x, 1.5-y, 1/2+z
C18	1	1.2018	0.8125	1-x, 1.5-y, 1/2+z
C17	1	1.3059	0.4177	1-x, 1.5-y, 1/2+z
C42	1	1.3402	0.3946	1-x, 1.5-y, 1/2+z
N47	1	1.0971	0.8736	1-x, 1.5-y, 1/2+z
C48	1	1.0901	0.8553	1-x, 1.5-y, 1/2+z
C30	1	1.0485	0.8571	1-x, 1.5-y, 1/2+z
C31	1	1.0759	0.8563	1-x, 1.5-y, 1/2+z
C35	1	1.0556	0.8773	1-x, 1.5-y, 1/2+z
C37	1	1.0688	0.8361	1-x, 1.5-y, 1/2+z
C39	1	1.0552	0.8365	1-x, 1.5-y, 1/2+z
C41	1	1.0692	0.8769	1-x, 1.5-y, 1/2+z
C63	1	1.034	0.8575	1-x, 1.5-y, 1/2+z
C64	1	1.0269	0.8374	1-x, 1.5-y, 1/2+z
N65	1	1.0136	0.8377	1-x, 1.5-y, 1/2+z

C66	1	1.0068	0.8575	1-x, 1.5-y, 1/2+z
C67	1	1.0136	0.8782	1-x, 1.5-y, 1/2+z
C68	1	1.0272	0.878	1-x, 1.5-y, 1/2+z
C69	1	1.0068	0.8986	1-x, 1.5-y, 1/2+z
C6	1	1.3262	0.354	1+x, 1+y, z
C9	1	1.1803	0.8748	1+x, 1+y, 1+z
C9	1	0.8197	0.8748	1-x, 1-y, 1+z
C28	1	1.347	0.3741	1+x, 1+y, z
C42	1	1.1598	0.8946	1+x, 1+y, 1+z
C42	1	0.8402	0.8946	1-x, 1-y, 1+z

References

(1) Fernández-Ruiz, R.; Redrejo, M. J.; Friedrich, E. J.; Ramos, M.; Fernández, T. Evaluation of Bioaccumulation Kinetics of Gold Nanorods in Vital Mammalian Organs by Means of Total Reflection X-Ray Fluorescence Spectrometry. *Anal. Chem.* **2014**, *86* (15), 7383–7390. https://doi.org/10.1021/ac5006475.

(2) Fernández-Ruiz, R.; von Bohlen, A.; Friedrich K, E. J.; Redrejo, M. J. Analysis of Coke Beverages by Total-Reflection X-Ray Fluorescence. *Spectrochim. Acta Part B At. Spectrosc.* **2018**, *145*, 99–106. https://doi.org/https://doi.org/10.1016/j.sab.2018.04.013.

(3) Tellis, J. C.; Primer, D. N.; Molander, G. A. Single-Electron Transmetalation in Organoboron Cross-Coupling by Photoredox/Nickel Dual Catalysis. *Science* **2014**, *345* (6195), 433-436. https://doi.org/10.1126/science.1253647.

(4) Lowry, M. S.; Goldsmith, J. I.; Slinker, J. D.; Rohl, R.; Pascal, R. A.; Malliaras, G. G.; Bernhard, S. Single-Layer Electroluminescent Devices and Photoinduced Hydrogen Production from an Ionic Iridium(III) Complex. *Chem. Mater.* **2005**, *17* (23), 5712–5719. https://doi.org/10.1021/cm051312+.

(5) Rodríguez, A. A.; Garduño, J. A.; García, J. J. Nickel(Ii) and Nickel(0) Complexes as Precursors of Nickel Nanoparticles for the Catalytic Hydrogenation of Benzonitrile. *New J. Chem.* **2020**, *44* (3), 1082–1089. https://doi.org/10.1039/C9NJ05221F.

(6) Jiménez-Almarza, A.; López-Magano, A.; Marzo, L.; Cabrera, S.; Mas-Ballesté, R.; Alemán, J. Imine-Based Covalent Organic Frameworks as Photocatalysts for Metal Free Oxidation Processes under Visible Light Conditions. *ChemCatChem* **2019**, *11* (19), 4916-4922. https://doi.org/10.1002/cctc.201901061.

(7) López-Magano, A.; Platero-Prats, A. E.; Cabrera, S.; Mas-Ballesté, R.; Alemán, J. Incorporation of Photocatalytic Pt(II) Complexes into Imine-Based Layered Covalent Organic Frameworks (COFs) through Monomer Truncation Strategy. *Appl. Catal. B Environ.* **2020**, *272*, 119027. https://doi.org/https://doi.org/10.1016/j.apcatb.2020.119027.

(8) Sun, G.; Wang, Z. Molecular Iodine-Catalyzed Benzylation of Arenes with Benzyl Alcohols. *Tetrahedron Lett.* **2008**, *49* (33), 4929–4932. https://doi.org/https://doi.org/10.1016/j.tetlet.2008.05.146.

(9) Peña-López, M.; Ayán-Varela, M.; Sarandeses, L. A.; Pérez Sestelo, J. Palladium-Catalyzed Cross-Coupling Reactions of Organogold(I) Reagents with Organic Electrophiles. *Chem. – A Eur. J.* **2010**, *16* (32), 9905–9909. https://doi.org/10.1002/chem.201000726.

(10) Chen, C.-R.; Zhou, S.; Biradar, D. B.; Gau, H.-M. Extremely Efficient Cross-Coupling of Benzylic Halides with Aryltitanium Tris(Isopropoxide) Catalyzed by Low Loadings of a Simple Palladium(II) Acetate/Tris(p-Tolyl)Phosphine System. *Adv. Synth. Catal.* **2010**, *352* (10), 1718–1727. https://doi.org/10.1002/adsc.201000311.

(11) Amatore, M.; Gosmini, C. Synthesis of Functionalised Diarylmethanes via a Cobalt-Catalysed Cross-Coupling of Arylzinc Species with Benzyl Chlorides. *Chem. Commun.* **2008**, *40*, 5019–5021. https://doi.org/10.1039/B809626K.

(12) Dewanji, A.; Krach, P. E.; Rueping, M. The Dual Role of Benzophenone in Visible-

Light/Nickel Photoredox-Catalyzed C−H Arylations: Hydrogen-Atom Transfer and Energy Transfer. *Angew. Chemie Int. Ed.* **2019**, *58* (11), 3566–3570. https://doi.org/10.1002/anie.201901327.

(13) Nambo, M.; Keske, E. C.; Rygus, J. P. G.; Yim, J. C.-H.; Crudden, C. M. Development of Versatile Sulfone Electrophiles for Suzuki–Miyaura Cross-Coupling Reactions. *ACS Catal.* **2017**, *7* (2), 1108–1112. https://doi.org/10.1021/acscatal.6b03434.

(14) Molander, G. A.; Canturk, B. Preparation of Potassium Alkoxymethyltrifluoroborates and Their Cross-Coupling with Aryl Chlorides. *Org. Lett.* **2008**, *10* (11), 2135–2138. https://doi.org/10.1021/ol800532p.

(15) Shen, Z.-L.; Goh, K. K. K.; Yang, Y.-S.; Lai, Y.-C.; Wong, C. H. A.; Cheong, H.-L.; Loh, T.-P. Direct Synthesis of Water-Tolerant Alkyl Indium Reagents and Their Application in Palladium-Catalyzed Couplings with Aryl Halides. *Angew. Chemie Int. Ed.* **2011**, *50* (2), 511–514. https://doi.org/https://doi.org/10.1002/anie.201005798.

(16) Chen, Y.; Lu, P.; Wang, Y. 3-Amino-Fluorene-2,4-Dicarbonitriles (AFDCs) as Photocatalysts for the Decarboxylative Arylation of α-Amino Acids and α-Oxy Acids with Arylnitriles. *Org. Lett.* **2019**, *21* (7), 2130–2133. https://doi.org/10.1021/acs.orglett.9b00443.

(17) Karakaya, I.; Primer, D. N.; Molander, G. A. Photoredox Cross-Coupling: Ir/Ni Dual Catalysis for the Synthesis of Benzylic Ethers. *Org. Lett.* **2015**, *17* (13), 3294–3297. https://doi.org/10.1021/acs.orglett.5b01463.

(18) Lévêque, C.; Chenneberg, L.; Corcé, V.; Goddard, J.-P.; Ollivier, C.; Fensterbank, L. Primary Alkyl Bis-Catecholato Silicates in Dual Photoredox/Nickel Catalysis: Aryl- and Heteroaryl-Alkyl Cross Coupling Reactions. *Org. Chem. Front.* **2016**, *3* (4), 462–465. https://doi.org/10.1039/C6QO00014B.

(19) Zuo, Z.; Ahneman, D. T.; Chu, L.; Terrett, J. A.; Doyle, A. G.; MacMillan, D. W. C. Merging Photoredox with Nickel Catalysis: Coupling of α-Carboxyl Sp3-Carbons with Aryl Halides. *Science* **2014**, *345* (6195), 437-440. https://doi.org/10.1126/science.1255525.
